# Physical and functional interactome atlas of human receptor tyrosine kinases

**DOI:** 10.15252/embr.202154041

**Published:** 2022-04-05

**Authors:** Kari Salokas, Xiaonan Liu, Tiina Öhman, Iftekhar Chowdhury, Lisa Gawriyski, Salla Keskitalo, Markku Varjosalo

**Affiliations:** ^1^ Institute of Biotechnology HiLIFE University of Helsinki Helsinki Finland

**Keywords:** RTK, interaction proteomics, systems biology, receptor tyrosine kinase, phosphoproteomics, Proteomics, Signal Transduction

## Abstract

Much cell‐to‐cell communication is facilitated by cell surface receptor tyrosine kinases (RTKs). These proteins phosphorylate their downstream cytoplasmic substrates in response to stimuli such as growth factors. Despite their central roles, the functions of many RTKs are still poorly understood. To resolve the lack of systematic knowledge, we apply three complementary methods to map the molecular context and substrate profiles of RTKs. We use affinity purification coupled to mass spectrometry (AP‐MS) to characterize stable binding partners and RTK–protein complexes, proximity‐dependent biotin identification (BioID) to identify transient and proximal interactions, and an *in vitro* kinase assay to identify RTK substrates. To identify how kinase interactions depend on kinase activity, we also use kinase‐deficient mutants. Our data represent a comprehensive, systemic mapping of RTK interactions and substrates. This resource adds information regarding well‐studied RTKs, offers insights into the functions of less well‐studied RTKs, and highlights RTK‐RTK interactions and shared signaling pathways.

## Graphical abstract

A comprehensive, systemic map of human receptor tyrosine kinase interactions, substrates and functions, based on a combination of interaction proteomics methods and mass spectrometry‐based substrate mapping.

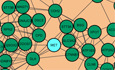

## Introduction

Protein phosphorylation reversibly controls the activity or localization of many proteins and is dynamically regulated by protein kinases and protein phosphatases, which phosphorylate and dephosphorylate proteins, respectively. Protein kinases catalyze the transfer of a phosphate group from ATP to threonine, serine, and tyrosine amino acids of specific target proteins. Currently, 571 human protein kinases have been identified. Of these, 137 are tyrosine kinases. Receptor tyrosine kinases (RTKs) are a subclass of tyrosine kinases that act as initiators, amplifiers, and central nodes in a plethora of complex biological functions and are mainly associated with intercellular communication. RTKs regulate key properties of their substrate proteins, which are essential for the coordinated actions of biological pathways and processes. Similar to other kinases, RTKs are strongly associated with a multitude of human diseases, such as cancer and a variety of multifactorial diseases and developmental disorders (McDonell *et al*, 2015).

In the human genome, 58 RTKs have been identified (Robinson *et al*, 2000). These RTKs are classified into 20 different subfamilies containing between 1 and 14 members. The Ephrin receptor subfamily is the largest, with 14 members (Pasquale, 2005; Liang *et al*, 2019), followed by the PDGF subfamily, which includes 5 RTKs (Demoulin & Essaghir, 2014; Kazlauskas, 2017), and the ErbB (Warren & Landgraf, 2006; Hynes & MacDonald, 2009) and FGF groups (Turner & Grose, 2010; Goetz & Mohammadi, 2013), each with 4 members. The other subfamilies have three or fewer members. While some RTKs, such as EGFR or ERBB2 (also known as HER2), have been extensively studied, most RTKs have been less well studied and have few known interactors; consequently, our understanding of their substrates or protein–protein interaction (PPI) partners is quite limited.

RTKs are thought to exist on the cell membrane as monomers, dimers, and oligomers. While dimerization or oligomerization is required for activation (Lemmon & Schlessinger, 2010), not all dimers or oligomers actively signal (Gadella & Jovin, 1995; Clayton *et al*, 2005; Ward *et al*, 2007). Once oligomerization has occurred, the intracellular domains can transphosphorylate one or more tyrosine in neighboring RTKs. In addition to canonical cell surface signaling, nuclear signaling activity has also been identified for multiple RTKs (Song *et al*, 2013). The phosphorylated receptor serves as a platform for the assembly and activation of intracellular signaling intermediaries. An inactive kinase is in an autoinhibitory conformation, and this conformation is released by the phosphorylation of an activation loop, after which signaling can proceed. Protein kinases are kept inactive by phosphatases. Protein tyrosine phosphatases (PTPs), in addition to deactivating RTKs when appropriate, also function to maintain RTKs in an inactive state. Indeed, inducing the activation of RTKs is possible in one of two ways: ligand binding or inhibition of cellular phosphatases (Ostman & Böhmer, 2001; Reynolds *et al*, 2003; Tonks, 2006). PTPs, in turn, can be inhibited *in vitro* with vanadate or pervanadate, leading to tyrosine kinase activation (Zhao *et al*, 1996; Huyer *et al*, 1997; Boersema *et al*, 2010).

RTKs exert changes, via interactions with other proteins and by phosphorylating their substrate proteins. The interactions can be stable, as in the case of stable protein complexes, or they can be short‐lived transient associations. Therefore, to understand the role of RTKs in cellular signaling networks, it is vital to map their PPI networks. This goal, however, is hindered because a large number of RTKs have few known interactors. Two well‐established and reliable methods for mapping PPIs by mass spectrometry are affinity purification coupled to mass spectrometry (AP‐MS) and proximity‐dependent biotin identification (BioID). AP‐MS captures stable interactions and can quantitatively capture other complex components in addition to direct interactors. BioID, in contrast, does not require a stable interaction but can also capture transient interactions within an ~ 10 nm radius. Multiple prey proteins may be identified with multiple baits, which suggests that these proteins participate in the same process or protein complex (Drew *et al*, 2017; Knight *et al*, 2017; Youn *et al*, 2018).

In this study, we performed systematic AP‐MS and BioID analyses of ~ 90% of human RTKs in their activated state. This set of 52 RTKs included 7 RTKs with fewer than 20 previously identified interactors. The generated interactome network included > 6,000 unique high‐confidence RTK–protein interactions. Furthermore, to detect interactions that depended on the corresponding kinase activity, we used kinase activity‐deficient (KD) mutants for 11 RTKs. Additionally, we used a phosphoproteomic approach to identify substrates for 45 RTKs. The results represent a comprehensive RTK interaction network and reveal central pathways through which RTKs may exert their effects, as well as networks of probable associations between interactor proteins and RTK‐specific functional enrichment.

## Results

### Defining the RTK interaction landscape

To comprehensively identify RTK‐interacting proteins, we used two complementary methods, AP‐MS and BioID MS. First, 52 human RTKs were cloned into the MAC‐tagged expression vector (Liu *et al*, 2018) and inducibly expressed in 52 stable cell lines, generated from the HEK293‐Flp‐In T‐REx cell line. The HEK293 cell line was chosen due to the ready availability of Flp‐In T‐Rex system, and the cell line’s extensive utilization in both large‐scale proteomic investigations, as well as RTK studies in particular (Yao *et al*, 2017; Buljan *et al*, 2020; Go *et al*, 2021).

The HEK293 cell line is one of the cell lines expressing the highest number of RTKs, according to the human protein atlas project (Thul & Lindskog, 2018). When we investigated which cell lines had any detectable expression of the RTKs included in this study, only three cell lines had more RTKs expressed than HEK293: SCLC‐21H, NTERA‐2, and U‐2 OS with 50, 48, and 46 RTKs expressed, respectively, compared to the 43 of HEK293. After filtering the values with normalized transcript expression value (nTPM) of 1, 34 RTKs passed the filter in HEK293 cells, while U‐2 OS had 36 RTKs, and SCLC‐21H and NTERA‐2 had 31 and 34, respectively. We next investigated the data of the human cell map project for protein‐level RTK expression detection. The project utilized the HEK293 Flp‐In T‐Rex cell line to generate a proximity biotinylation map of the human cell (Go *et al*, 2021). While the project makes no attempt to characterize the expression levels of any protein family, in their data, we found 26 RTKs as preys (signifying endogenous expression), of which 3 (INSRR, TEK, EphA1) were not detected in the protein atlas HEK293 data at all, and further 3 (RON, RET, DDR2) were detected with nTPM values smaller than 1. Taken together the expression data from the protein atlas and protein‐level evidence from the human cell map, HEK293 is one of the more RTK‐rich cell lines available, and perhaps the richest, if only considering cell lines for which inducible, isogenic expression systems are available.

**Figure EV1:**
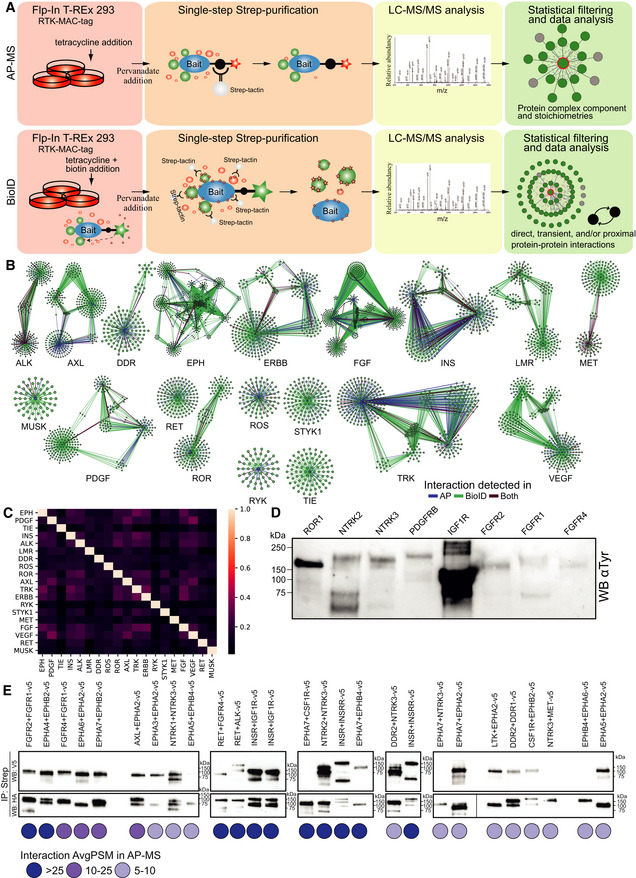
Detailed workflows used in this study and overall data assessment Workflows used in this study for affinity purification coupled with mass spectrometry (AP‐MS) and BioID approaches. AP‐MS enables the capture of protein complexes and stoichiometries, whereas the complementary BioID method enables capture of direct, transient/proximal interactions.Network topologies of RTK subfamilies. Blue nodes indicate the bait protein used in the experiment and green nodes the detected HCI proteins. Interactions detected in AP‐MS only are marked in blue, BioID only in green, and interactions detected with both approaches are shown in burgundy.Proportion of HCIs shared between RTK subfamilies. Values are calculated based on the number of shared HCIs and the number of HCIs in each subfamily.Anti‐phosphotyrosine blot for 8 RTKs, showing bait RTK phosphorylation.Co‐IP validation of RTK–RTK interactions detected via AP‐MS method in the study. Spectral count values for the interactions are shown underneath. Source data are available online for this figure.

For all RTKs, a C‐terminal MAC‐tag was used, in order to ensure tagging of intracellular interactors in BioID experiments. The tag consists of two Strep‐Tag II sequences, followed by HA, and finally the BirA* enzyme with linker sequences in between. While it is possible that the C‐terminal tag might affect protein binding with some partners, this likely only affects the AP‐MS experiments, and moving the tag to the N‐terminus would pose major problems to the BioID experiments because the N‐terminus of RTKs is extracellular. Each of these cell lines had the corresponding MAC‐tagged RTK incorporated in a single genomic locus, from which expression could be induced with tetracycline. AP‐MS allows the capture of stable interactions and the derivation of complex stoichiometry, while BioID can also detect proximal and transient interactions (Liu *et al*, 2018) (Fig EV1A). To capture the interactions of active RTKs, cellular PTPs were inhibited with pervanadate prior to sample collection. Pervanadate irreversibly inhibits PTPs by modifying the catalytic cysteine of the PTPs (Huyer *et al*, 1997).

**Figure 1:**
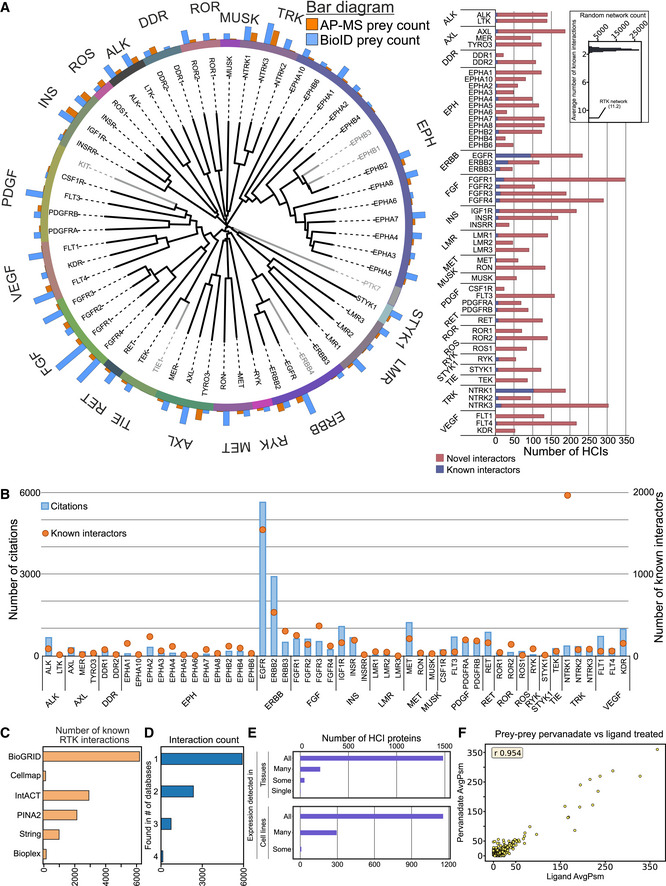
General assessment of study scope and interaction data landscape Left: Sequence alignment tree of the receptor tyrosine kinase (RTK) family. Members of the 20 different receptor tyrosine kinase subfamilies are grouped according to their sequence (kinase domain) homology to their respective subfamilies, indicated by the unique colors. Gray color indicates RTKs not included in this study. Number of high‐confidence interactor (HCI) proteins identified in AP‐MS (orange) and BioID (blue) experiments are indicated above the circle. Right: Comparison of the detected interactions to existing knowledge. The number of HCIs detected in this study are divided to reported interactions reported in at least one of the databases used for mapping known interactions (blue), and novel interactions (red). Inset: Average number of known protein–protein interactions in 100,000 randomly generated networks of identical topology, as the RTK network generated during this study. The average number of known interactions per bait for the RTK network is annotated with a pointer. Interactions reported here represent both AP‐MS and BioID results. For both of these methods, two biological replicates were analyzed.Number of citations and known interactors per RTK, which are grouped into their respective subfamilies. Citations are shown in blue bars and plotted against the Y‐axis on the left, while known interactors are shown with orange bubbles, and the right axis. Citations were mapped from NCBI gene2pubmed data.Number of known RTK interactors from each of the databases used for the known set. For all known interaction analyses of this study, the six different databases were merged into one dataset.Known RTK interactors grouped based on how many of the six used databases they were seen in. No interactors were seen in five or more databases, and most were only seen in one.Expression of identified HCI proteins in tissues (top) and cell lines (bottom) from human protein atlas (Uhlén *et al*, 2015). Detected in all: expression detected in all available tissues or cell lines; detected in many; detected in at least a third of the tissues/cell lines; detected in some; detected in more than one, but fewer than a third of the tissues/cell lines.Average peptide spectrum match (AvgPsm) comparison between pervanadate (Y‐axis) and ligand (X‐axis) treated samples for 8 RTKs. Correlation coefficient was calculated using Pearson r method of the SciPy stats package.

The 52 RTKs (> 90% of all human RTKs) studied here include all RTK subfamilies (Fig 1A) (Lemmon & Schlessinger, 2010). Although the data includes four pseudokinases (ERBB3, EphB6, EphA10, STYK1) and others, which are suspected to be pseudokinases (ROR1, ROR2, RYK), they were included in the study for data completeness. For the same reason, we have included the LMR family in the analysis. Although LMRs have historically been associated with the RTK family (Lemmon & Schlessinger, 2010; Butti *et al*, 2018), they have recently been removed from the receptor family (Trenker & Jura, 2020; Wendler *et al*, 2021), and classified as serine/threonine kinases.

The RTKs that were not studied in detail (EphB1, EphB3, ErbB4, KIT, PTK7, and TIE1) consist of RTKs for which we could not generate a MAC‐tagged expression clone. After stringent statistical filtering, we identified 6,050 unique high‐confidence interactors (HCIs) (Dataset EV1A). A total of 1,145 interactions were identified with AP‐MS, 4,497 with BioID, and 408 with both methods. The interactors consisted of 1,521 unique proteins. The number of identified interactors varied significantly between individual kinases, but many RTK subfamilies showed similar numbers of interactors. The number of known interactions identified was significantly higher than what would be expected from random interaction networks with the same topology as the RTK network (Fig 1A, inset). The information gathered in this study therefore supplements the scarce interaction data available for many less well‐studied RTKs.

Fifteen RTKs had more than 150 identified interactors, and the remaining 37 had fewer interactors (Fig 1A). While some RTKs have been well‐studied with many known interactions, most have only a few reported interactions (Fig 1B), highlighting the need for a systematic study. The number of known interactors for RTKs generally follows the number of citations for each RTK (Fig 1B), and indeed 19 RTKs had fewer than 100 publications associated with them in the NCBI publication database. For known interactions, we utilized a database combining six databases of interactions (Fig 1C).

We next decided to characterize the distribution of the known interactors across the six (6) databases from which they were taken. BioGRID, IntACT, and PINA2 contributed the highest number, followed by String, and finally bioplex and human cell map. To characterize how commonly seen the known interactors were, we next analyzed how many databases each interaction was featured in (Fig 1D). While most interactions were only seen in one database, roughly a third of the interactions were shared between two or more. The largest proportion were seen at least two databases as expected, considering the complementary nature of BioGRID, IntAct, and PINA2.

Many RTKs share interactions with members of their own subfamily (Fig EV1B). While most subfamilies have a high degree of interconnected interactors, each RTK in this study has identified HCIs, which were not shown to interact with other members of their respective subfamilies. For example, the Eph subfamily has many shared interactions, while the ERBB, INS, and LMR subfamilies have fewer shared interactions, which may indicate similar functions within the Eph family. A second source of variability is the interaction types themselves. BioID interactions represent a higher proportion of all interactions in all subfamilies, except for ROS. However, in different subfamilies, the proportion of BioID interactions varied from 87% with VEGF to 40% with ROS. Shared interactors between receptors in the same subfamily were often identified with both methods (e.g., the shared cluster in the ERBB subfamily): 27% of the interactions shared were detected with both methods, whereas 15% of interactions overall were detected with both methods. The higher percentage may suggest the presence of proteins that are instrumental to the overlapping functions of the receptors in the subfamily. Interactors were widely shared across subfamily boundaries as well. We detected 675 interactors shared within subfamilies and 728 shared with receptors in another subfamily (Fig EV1C, Dataset EV1A). Common HCIs may suggest potential RTK functional overlap and crosstalk, while unique HCIs may indicate receptor‐specific functions and RTK‐specific variations in possible shared pathways.

**Figure EV2:**
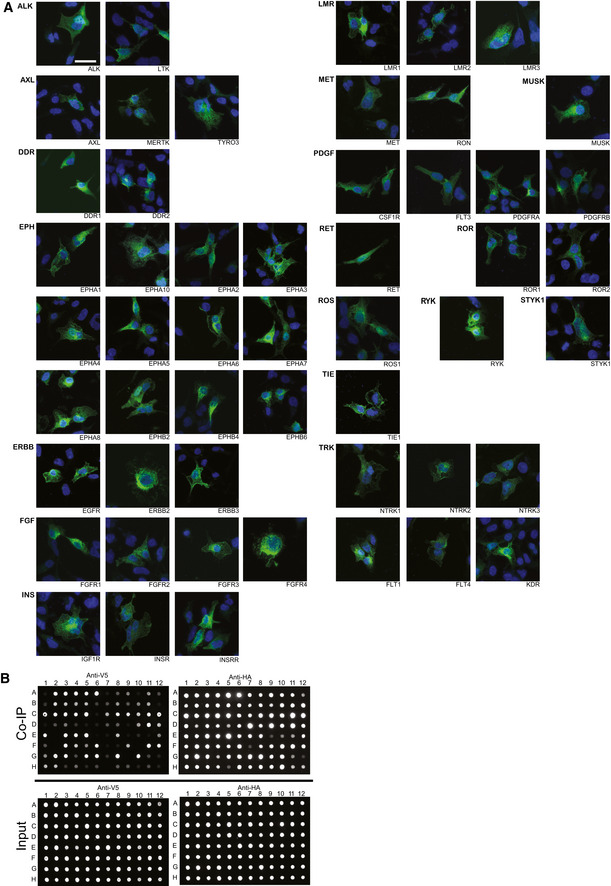
RTK localization and AP‐MS validation via CO‐IP Immunofluorescence microscopy images of each bait RTK. Images are divided based on the RTK subfamily. Green: Anti‐HA, Blue: DAPI. Scale bar 10 µm.Verification of several AP‐MS high‐confidence interactions with Co‐IP and dot blot. 83 interactions were tested, of which 69 were detected via Co‐IP. The prey proteins were tagged with Strep‐HA, and bait proteins with V5 and coexpressed in HEK293 cells. For negative controls, Strep‐HA‐tagged GFP and V5‐tagged RTKs were used. The Strep‐HA tagged proteins were immunoprecipitated with Strep‐Tactin sepharose. The immunoprecipitated protein complexes were then dot blotted with anti‐V5 and anti‐HA antibodies.

To determine whether we could identify indications of the active state of the bait RTKs, we analyzed the AP‐MS data for known autophosphorylation site(s) for each RTK. For the majority of RTKs, we identified known tyrosine autophosphorylation site(s) as phosphorylated site(s) (Dataset EV2). In order to further validate the phosphorylation status of the bait RTKs, we performed an anti‐phosphotyrosine western blot (WB) analysis of a subset of the RTKs (Fig EV1D) and detected phosphorylation in all of the 8 RTKs analyzed. To ensure that MAC‐tagged RTKs localize to plasma membrane, we carried out immunofluorescence confocal microscopy imaging for all of the baits included in the study (Fig EV2A). In the images, we detected signal from the cell membrane, as expected, as well as some signals from other cellular compartments. These included ER and endosomal signals, which may suggest RTKs to localize in some extent to various membrane compartments. Endosomal and ER localization may be due to both physiological activity (identified for many RTKs; for reviews, see Miaczynska, 2013; Fraser *et al*, 2017; and Farhan, 2020) and some unspecific antibody staining.

Given the varied expression of RTKs across tissues and cell types, we also decided to analyze whether the interactions detected could be cell‐line specific, or proteins that are expressed in a variety of tissues. For this purpose, we mapped expression level data from the human protein atlas (Uhlén *et al*, 2015) project (Fig 1E). We next divided the identified interactors based on annotations of the database into proteins that were detected in all, many (≥ 33%), some (> 1), or one cell line or tissue type. The majority of our unique interactors were seen across all tissues and cell lines included in the atlas, while fewer than 300 were seen in many, and fewer than 100 in some or only one. As validation of BioID‐detected interactions is difficult, we instead chose to validate tens of AP‐MS interactions with an orthogonal method, co‐immunoprecipitation (Fig EV2B, Dataset EV1C). Out of the 83 interactions tested, the Co‐IP experiment detected 69. The unconfirmed interactions may still represent interactions mediated by a third protein between the RTK and the interactor (e.g., in protein complexes).

We next utilized a subset of RTKs to investigate the effect of pervanadate treatment in comparison to ligand‐induced activation. We performed side‐by‐side AP‐MS and BioID experiments with pervanadate‐treated and ligand‐treated cell lines of 8 RTKs (EGFR, FGFR1, FGFR4, IGF1R, INSR, INSRR, PDGFRB, and RET). For these RTKs, the main ligand was known, and they were available as recombinant proteins with validated activity. From these experiments, we identified in total 1,132 high‐confidence interactions, consisting of 595 unique proteins. Of these, ~ 80% (872) of the HCIs were seen in both pervanadate‐ and ligand‐treated samples. The majority of the prey proteins were seen with similar spectral count values in both experiments (correlation value 0.954, Fig 1F). Of the interactions seen only in ligand‐treated samples, 83 were detected with an average spectral count of over 5. Of these, 61 were seen only in AP‐MS experiments, 18 in BioID, and 4 in both (Dataset EV1D). Likewise, 25 HCIs were seen only in pervanadate‐treated samples (14 AP‐MS only, 10 BioID, and 1 in both). On the functional level, however, the proteins which were seen only in either pervanadate‐ or ligand‐treated experiments fell into the same functional groups with proteins that were identified in both experiments (Dataset EV1E). 17.1% of all interactions identified in the ligand experiments were previously reported, while 17.3% of the interactions only detected with the ligand treatment were previously known. Of the interactions only seen with pervanadate treatment and not ligand treatment, 20.6% were previously known. Together, this suggests that our pervanadate treatment does capture functionally relevant interactions, and results from both treatment strategies fit existing knowledge roughly equally well, although the specific details may differ, as illustrated by the pervanadate‐only and ligand‐only interactions. Considering specific ligands are not available or even known for all RTKs and the advantages of having similar experimental background for all receptors, we therefore considered our pervanadate‐mediated activation of RTKs adequate and able to functionally replicate the interactomic aspects of RTK activation. However, the results do not reflect complete interactomes of the RTKs studied. Our approach does not identify interactors requiring specific molecular context to exist in and around the cell (e.g., presence of different combinations of ligands, or the activation or inactivation of other signaling networks), and the AP‐MS and BioID methods likewise do not identify 100% of the proteins in any given sample.

**Figure EV3:**
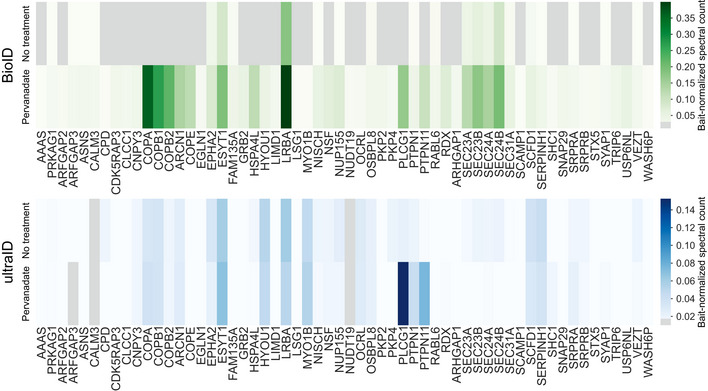
15‐min pervanadate treatment compared to no treatment with NTRK3 BioID and ultraID experiments HCI proteins of pervanadate‐treated NTRK3 detected by proximity labeling approaches (BioID or UltraID) are shown to highlight what interactions are treatment‐dependent. With ultraID (preprint: Zhao *et al*, 2021), 10‐min biotinylation time was used for identifying interactions at the time of activation. For visualization purposes, only proteins with significant changes (bait‐normalized average spectral count difference > 2 fold, or exclusively identified in pervanadate treatment) were selected to be presented here.

With AP‐MS, the activation of RTKs immediately prior to harvesting is a sound strategy. However, with BioID, the 24‐h biotin treatment enables each RTK to label potential interactors over the lifetime of the receptor, instead of only at the moment of activation. We therefore investigated which interactors can be expected to require pervanadate‐induced RTK activation. For this purpose, we used NTRK3 as a pilot experiment to compare pervanadate treated and untreated samples with the BioID method. We repeated this experiment using ultraID (preprint: Zhao *et al*, 2021) instead of BioID, and compared the results based on the BioID‐identified HCIs. UltraID is the latest development of proximity labeling approaches, offering superior labeling efficiency compared to BioID, BioID2, and APEX (preprint: Zhao *et al*, 2021). It is currently the smallest proximity‐dependent biotinylation enzyme and can efficiently label proximal interactors even in 10 min. We could therefore utilize ultraID to detect interactors only at the time of pervanadate‐induced activation of NTRK3, instead of over the preceding 24 h. We next focused only on proteins, which were only seen in pervanadate‐treated samples, or whose bait‐normalized spectral count value was less than half of pervanadate‐treated samples (Fig EV3). This group includes several proteins whose functions can be seen as pivotal to activated RTKs (for example, phosphatases PTPN1 and PTPN11, and RTK‐activated signaling proteins PLCG1 and GRB2). Although the BioID and ultraID results are in agreement in regards to PLCG1, PTPN1, and PTPN11, in ultraID samples, GRB2 was equally present in both treated and untreated results. Taken together, the results suggest that while we do identify important RTK interactors without pervanadate as well, the treatment enhances identification of typical RTK‐dependent interactors such as PLCG1. The data suggest that with pervanadate‐induced activation of RTKs, we may gain a more complete set of interactors of active RTKs.

### Kinase–kinase interactions between RTKs

**Figure 2:**
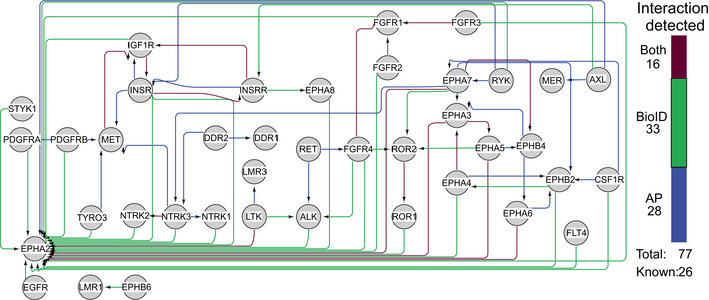
RTK bait–bait interactions High‐confidence bait–bait interactions were detected between the RTKs. Connections are colored based on whether they were detected in AP‐MS (blue), BioID (green), or both (burgundy). In total, 77 RTK–RTK interactions were identified, of which 26 were previously known, 28 of the interactions were seen only in AP‐MS data, 33 in BioID, and 16 with both methods.

To investigate whether RTK heterodimers or ‐oligomers contributed to the number of identified shared HCIs, we next investigated the presence of RTK–RTK interactions in detail (Fig 2). In total, we identified 77 RTK–RTK interactions, of which 33 were between receptors in the same subfamily. The majority of these subfamily interactions (27) were detected either with AP‐MS or both AP‐MS and BioID. In contrast, 27 of the 44 interactions between receptors in different subfamilies were detected via BioID only. The identifications derived from BioID alone could more specifically indicate membrane areas and structures commonly shared between the RTKs than identifications derived by other methods. However, the 16 RTK–RTK interactions that were detected by both methods and 28 detected via AP‐MS alone suggest the formation of a wide variety of stable RTK–RTK heterodimers. While heterodimerization is a well‐documented phenomenon in RTKs, many of the specific interactions here have not been documented previously. Eighteen of the 77 (23%) were previously known, leaving 59 (77%) novel interactions. To validate the RTK–RTK interactions, we performed co‐IP analysis of 27 RTK–RTK interactions that were seen in AP‐MS data, and detected the interactions with all but 4 of them (Fig EV1E), possibly indicating that these 4 interactions are not direct but mediated by another protein in the same complex. In the ligand‐activation AP‐MS experiments discussed previously, we identified four interactions (EGFR‐MET (known), EGFR‐INSR, FGFR1‐IGF1R, and FGFR1‐MET), which were not seen with pervanadate treatment. Likewise, in our pervanadate experiments, we saw two RTK–RTK interactions (FGFR1‐FGFR2 (known) and EGFR‐EphA2), which were not in the ligand data. Based on these results, we can expect that the pervanadate treatment does not seem to produce RTK–RTK interactions that would not be seen in normal cellular conditions. The EGFR‐EphA2 interaction, while not in our combined database of known interaction, has also been reported previously (Swidergall *et al*, 2021). Here, as with interactions with non‐RTK proteins, it is quite likely that not all interactions can be induced in cell culture conditions, with pervanadate treatment, or be captured with the AP‐MS and BioID workflows.

Interestingly, EphA2 was seen with a majority of RTKs (30 in total), although it was previously known to form complexes only with EGFR, ErbB2, EphA7, DDR1, and NTRK3 (Larsen *et al*, 2007; Brantley‐Sieders *et al*, 2008; Zhuang *et al*, 2010; Oricchio *et al*, 2011; Lemeer *et al*, 2012; De Robertis *et al*, 2017; Huttlin *et al*, 2017). Through AP‐MS only or both methods, we detected six interactions between EphA2 and another RTK (AXL, EphA3, EphA5, EphA6, EphA7, and LTK). Of these, only EphA7 is a previously known interactor. To our knowledge, EphA2 is not highly expressed in HEK‐293 cells (Dataset EV1B); hence, its wide identification is unlikely to be due to expression levels. It may therefore be possible that the identified AP‐MS interactions of RTKs with EphA2 represent heterocomplexes, while proximal or transient interactions may be due to localization with similar membrane and internalization compartments.

### RTK interactors participate in complexes in a wide variety of cellular compartments

**Figure EV4:**
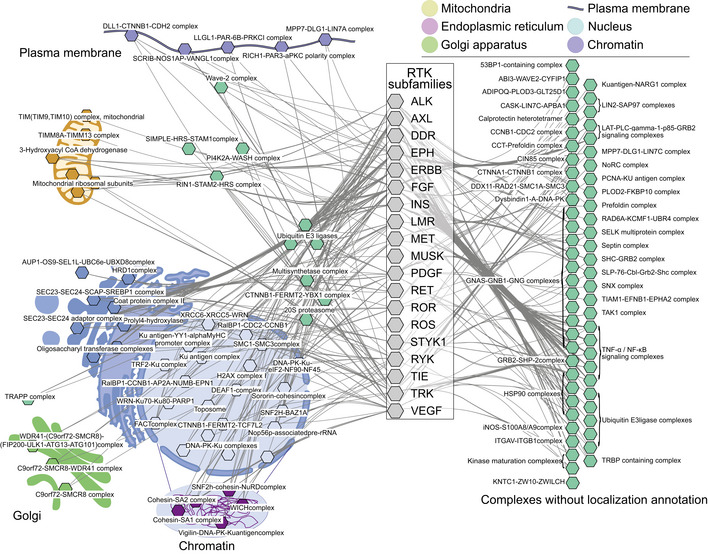
Enriched complexes in the RTK interactome data Significantly (q < 0.05, calculated with Fisher exact test followed by Benjamini–Hochberg multiple‐testing correction) enriched CORUM complexes in the interactomes of the RTK subfamilies. The cellular localization was assigned to each complex with available GO cellular component in CORUM. Connections from subfamilies to complexes denote significant enrichment of the complex with one or more members of the subfamily. On the right side, complexes without localization information are grouped based on their protein composition.

In the interaction data gathered thus far, we wanted to investigate the presence of protein complexes, which may be connected to RTK signaling in the cell. To this end, we performed enrichment analysis of CORUM (Giurgiu *et al*, 2019) complexes for each RTK and then grouped the results based on the gene ontology cellular component (GOCC) annotations, if available in CORUM. Although many of the complexes had no localization annotations available, very thorough coverage of the cell was seen in the complexes that were able to be assigned to a locale (Fig EV4). Curiously few strictly plasma membrane complexes were seen in the data. However, this may be in part due to imperfect coverage of GOCC annotations in CORUM and in part due to strict filtering applied to the data.

In total, 208 unique complexes were enriched in the data (Dataset EV3), and we were able to assign probable localizations to 59 of these based on CORUM annotations. These assignments included 5 plasma membrane and 8 ER complexes (two of which were specific ER‐membrane complexes), 5 chromosomal complexes, and 21 other nuclear complexes. Other complexes enriched in the RTK interactor sets were two kinase maturation complexes and five different TNF‐alpha/NF‐kappa B signaling complexes. The most commonly enriched complex was the LTC‐PLC‐gamma‐1‐p85‐GRB2‐SOS signaling complex, which was enriched in 27 RTKs. The first of many ER protein complexes, coat protein complex II (COPII), was the second most common and was enriched with 21 RTKs. This complex shares many components with the two SEC23 complexes, which were also enriched in 21 RTKs.

Additionally, 26 nuclear complexes were identified. Based on the existing knowledge and GO annotations, some of these complex components identified in this study do appear to shuttle between cytoplasm and nucleus, and even to the plasma membrane. However, the majority of the components in these complexes are strictly nuclear. Nuclear signaling is a well‐documented, noncanonical mode of signaling for many RTKs (Carpenter, 2003; Krolewski, 2005; Massie & Mills, 2006; Schlessinger & Lemmon, 2006; Song *et al*, 2013). In our HCI data, we detected 93 exclusive nuclear proteins with 40 different RTKs and 909 proteins with some activity in the nucleus according to GOCC classifications. Among these 40 RTKs, MER and FLT3 had the most interactions (22 interactions). Every RTK had interactors to some extent with connection to the nucleus: DDR1, a collagen receptor, had the fewest (12, none of which were strictly nuclear). FGFR1 had the most (172), reflecting its important role in signaling functions in the nucleus (Stachowiak *et al*, 1996; Myers *et al*, 2003). Nuclear interactors identified during the course of the study may stem from valid interactions, or some may be experimental artifacts. While it is possible that some nuclear interactions detected via AP‐MS could stem from binding post‐lysis in ice‐cold conditions (though unlikely), and some interactions detected via either method can be proteins encountered only during mitosis after nuclear breakdown, the data may also offer some additional context for possible connections between RTKs and nuclear signaling pathways.

The 4 identified HSP90‐related complexes, which were significantly enriched with 47 different RTK baits, are of interest for the regulation of kinase activity. Considering the role of HSP90 in fostering and promoting proper protein folding and function, we next examined this link in detail. Of the 29 RTK baits that have previously been studied as potential interactors for the HSP90 complex (Taipale *et al*, 2012), 15 were strong interactors, 10 were weak interactors, and 4 were not interactors. Of the 3 HSP90 proteins of interest, CDC37, HSP90AA1, and HSP90AB1 were all identified with 11 RTKs, of which FGFR4 was not included in the Taipale *et al* (2012) study, and TYRO3 was classified as a weak interactor (Dataset EV4). The nine others were strong interactors. CDC37 and HSPAA1 were identified as HCIs with LMR1. CDC37 alone was identified with all but 5 baits (Dataset EV1A). Therefore, our findings were consistent with those of Taipale *et al* (2012). All three components were identified as HCIs for nine strong HSP90 interactors (Datasets EV1 and EV4). These interactions were detected mainly via AP‐MS, suggesting stable interactions. The only weak interactor that was detected with all three components, TYRO3, has since been linked to two HSP90 core interactor proteins (Li *et al*, 2018). FGFR4, which was not included in the Taipale *et al* (2012) study, was identified with all three components by AP‐MS, indicating that FGFR4 is a potential HSP90 interactor kinase.

### Enriched protein domains and functions of RTK interactors

**Figure 3:**
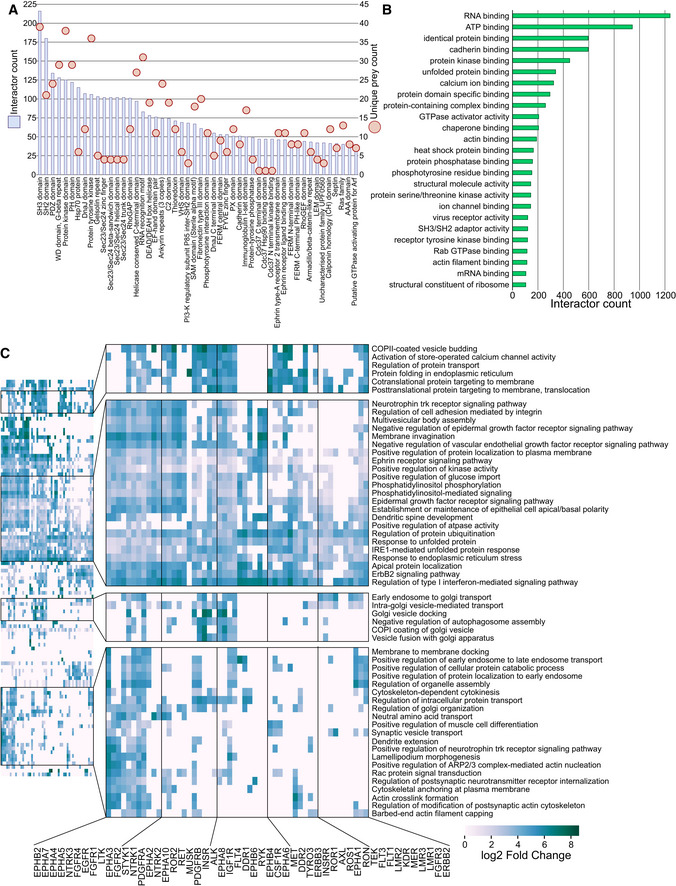
Characterization of RTK interactor proteins Identified protein domains of the RTK interactors mapped from Pfam. Blue bars (left Y‐axis) denote the cumulative count of the corresponding domain, while light red circles (right Y‐axis) denote the count of unique prey proteins with the domain (i.e., SH3 domain was encountered 216 times in the data, but in 39 unique proteins, while SH2 domain was identified 180 in 21 unique HCIs).Significantly enriched (q < 0.05, calculated with Fisher exact test and Benjamini–Hochberg multiple‐testing correction) GO “molecular function” annotations in the RTK interactors.Significantly enriched signaling pathways (reactome) identified in each RTK interactome. Fold change values were calculated using the human UniProt as the reference. Values are shown in log2 scale, and negative values were filtered out. A q‐value cutoff of 0.05 was used to identify significant fold changes (calculated using Fisher exact test with Benjamini–Hochberg correction)

Considering the enriched protein complexes identified, we next proceeded to investigate the domain composition of the individual HCI proteins (HCIPs). The top two domains identified by absolute counts were SH3 and SH2 (Fig 3A). When considering only unique HCIPs, SH3, the protein kinase domain and the protein tyrosine kinase domain were the most common. All of these domains play prominent roles in kinase signaling (Mayer, 2001; Xin *et al*, 2013). The SH3 domain was identified 216 times in 39 unique HCIPs, whereas the SH2 domain was identified 180 times in 21 unique HCIPs.

Twenty‐eight percent of all human proteins annotated with the protein tyrosine kinase domain were identified among the HCIPs, compared to 10% of proteins annotated with the protein kinase domain. SH2 domains suggest potential target proteins, since RTK activation via autophosphorylation induces the formation of SH2 domain binding sites (Lemmon & Schlessinger, 2010). Indeed, 43% of HCIPs with SH2 domains were previously known interactors of RTKs. To identify the specific functions these HCIPs participate in, we next examined GO molecular function terms associated with the identified HCIs. Similar to domains, the most common molecular functions associated with the HCIPs were related to protein kinase activities either directly (ATP binding), indirectly (protein kinase binding), or in a supporting role (heat shock protein binding) (Fig 3B).

To investigate functional similarities and differences between RTKs based on their interactions, we next performed a GO biological process (BP) analysis and highlighted the most enriched (log2‐fold change > 5) terms (Fig 3C). We identified four groups of terms containing processes related to RTK functions. These included terms enriched in most RTKs, such as multiple signaling pathways, and groups of more specialized terms, such as processes related to vesicle trafficking between the Golgi apparatus and the endosomal system.

Many of these processes are interlinked with known RTK functions. The ERBB2 signaling pathway, for example, was significantly enriched in almost all RTKs. Similarly, the type I interferon signaling pathway was seen in all but three RTKs. As a further example, the Ephrin receptor pathway also contains the majority of RTKs. Given that among the pathways enriched with the highest fold change values, few are limited to individual receptors. The functional enrichment results further indicate that RTKs share many pathways through which signaling may occur depending on cellular conditions, possibly including crosstalk between the receptors.

We next examined how the enriched GOBP terms were represented among all previously known RTK interactors (Appendix Fig S1A). In the analysis, some of the most common GOBP terms detected in our results, such as signal transduction, protein phosphorylation, and various signaling pathways (Appendix Fig S3A, upper panel), were prominently featured in the database of known RTK interactors as well (Appendix Fig S1A, lower panel). However, missing from the known interactors for many receptors were proteins connected to COPII vesicle coating and cargo loading, as well as PI3K activity regulation, all of which were common functions among the identified HCIs, possibly illustrating a gap in the previous knowledge concerning such interactors. For example, COPII vesicle coating, budding, and cargo loading related proteins are missing from the known interactors of both RET and PDGFRB, but are found in our dataset in both pervanadate‐ and ligand‐treated samples (Appendix Fig S1A, Dataset EV1C).

### RTK interactors form protein clusters with distinctive functions

**Figure 4:**
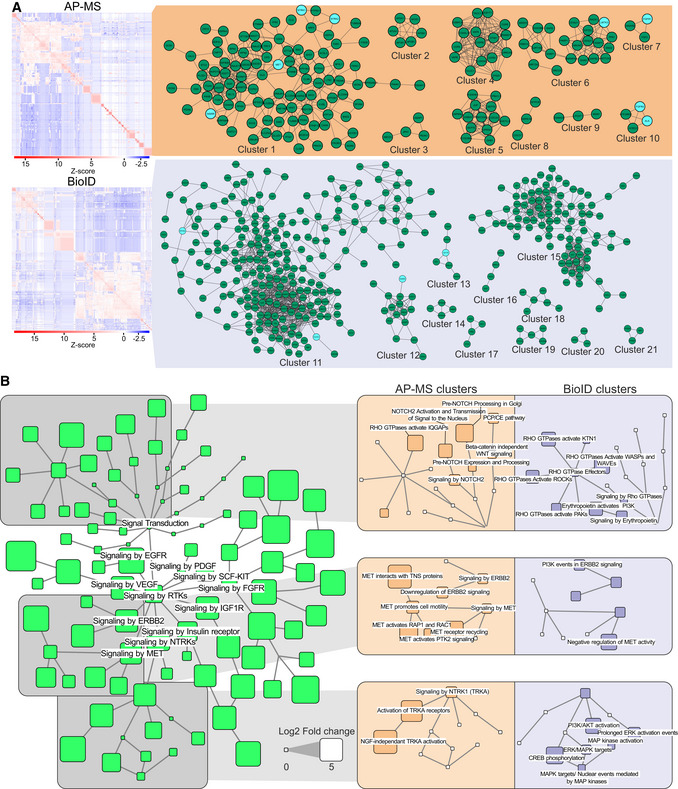
Functional clusters extracted from HCI cross‐correlation analysis HCI–HCI association clusters identified via cross‐correlation analysis of the identified RTK interactors. Clusters represent proteins, which are often co‐purified in our experiments. Clusters were identified separately from the AP‐MS or the BioID cross‐correlation data. RTKs, if any, in the clusters are shaded light blue.Enriched (log2FC > 5, q < 0.05, calculated with Fisher exact test and Benjamini–Hochberg multiple‐testing correction using the human UniProt as reference) reactome pathways in the identified association cluster. Nodes downstream from the signal transduction root node are shown. Node size corresponds to log2 fold change value of the pathway. Three pathway groups where AP‐MS and BioID clusters had the most prominent differences in enrichment are further highlighted in the boxes with orange (AP‐MS) and blue (BioID) background on the right.

Previously, protein copurification was investigated in large‐scale interaction studies to identify possible interactions between HCIPs. Affinity purification experiments showed that two proteins that purify together may indicate an interaction between them, such as a protein complex (Yu *et al*, 2009; Mehta & Trinkle‐Mulcahy, 2016; Buljan *et al*, 2020). Therefore, to understand how the RTK HCIs detected in our study might interact with one another, we performed a cross‐correlation analysis of both AP‐MS (Fig 4A, upper) and BioID (Fig 4A, lower) data. In total, 2,020 unique protein pair associations were detected through the two approaches (Dataset EV5). A total of 105 of these were previously known interactions, and 130 protein‐protein pairs were in the same reactome pathways. The analysis of random networks showed that this network was highly enriched in both known protein interaction pairs (Appendix Fig S1B, top) and proteins in the same reactome pathways (Appendix Fig S1B, bottom).

From the dataset, 21 clusters with 3 or more proteins were identified (Fig 4A). Of these, 10 were detected in AP‐MS data and 11 in BioID. In total, 7 of the clusters featured one or more RTKs as well. Reactome pathway enrichment analysis was performed for each protein cluster to identify what functions each could participate in. The proteins of the largest cluster detected in AP‐MS data, cluster 1 (Appendix Fig S1C, top left), functioned mainly in pathways such as small molecule transport, protein phosphorylation, and platelet signaling. The largest BioID cluster (11, Appendix Fig S1C, bottom right) featured proteins in particular from multiple signaling pathways, as well as vesicle trafficking and endocytosis in particular (Dataset EV6). From the clusters, we also identified CORUM protein complexes (Appendix Fig S1D). We filtered out all complexes from which less than 60% of the components were identified, and removed overlapping complexes, keeping the more complete ones. This resulted in 30 protein complexes identified from the cross‐correlation network.

We next linked the significantly enriched reactome pathway terms to the reactome hierarchy and extracted pathways linked to signal transduction (Fig 4B). Several signaling pathways were enriched, particularly with AP‐MS or BioID clusters. For example, RHO GTPase effector‐related pathways were enriched in BioID clusters, while Notch and WNT signaling were enriched in AP‐MS clusters. In the RTK pathways, we observed clear differences, particularly in the MET, ERBB2, and NTRK1 signaling pathway groups. These results suggest proximal RTK associations with functional protein networks related to RHO GTPase signaling, as well as MAPK and PI3K/AKT signaling. In contrast, the pathways enriched in the AP‐MS clusters may indicate a more direct role for RTKs in protein clusters related to Notch and WNT signaling. The presence of core RTK pathways, such as TRKA receptor activation or MET signaling in the AP‐MS clusters, strengthens the idea that RTKs have a more direct role in the pathways detected in AP‐MS clusters.

Ephrin receptors A5, A6, A7, and A8 are some of the less well‐studied RTKs (Fig 1B). We therefore analyzed their interactomes and the interplay between these receptors. To focus on the common HCIs, we removed interactors seen with only one of these receptors (Appendix Fig S2A). We identified the largest group of shared HCIs between EphA5 and EphA7, and there were 46 shared HCIs. In this group, we identified many other kinases, such as MAP4Ks and EphB4, and phosphatases, such as PTPN11 and PTPN13. We also identified 9 HCIs shared between all 4 of the Ephrin receptors and 16 shared between EphA4, A7, and A8. The shared groups included multiple proteins that are integral to the function of RTKs, such as SEC23B, SEC24A, and SEC24B, which participate in coat protein complex II, which may indicate the use of COPII‐coated vesicles in some portion of RTK membrane trafficking. When analyzing the interactions of enriched reactome pathways (Appendix Fig S2B, left side), we indeed observed multiple transport pathways, including endosome‐to‐Golgi and Golgi‐to‐ER pathways. The interaction data therefore indicate possible RTK paths through the cell. When examining the enriched CORUM complexes in detail (Appendix Fig S2B, right side), we identified the WAVE2 complex and other actin dynamics‐related factors, as well as oligosaccharyltransferase complexes responsible for co‐ and posttranslational glycosylation of proteins in the ER lumen. Thus, the interactomics data may be used to identify core RTK interactors shared between subgroups of receptors and possible avenues for cooperative RTK actions.

### Potential substrates define RTK kinase activity

**Figure 5:**
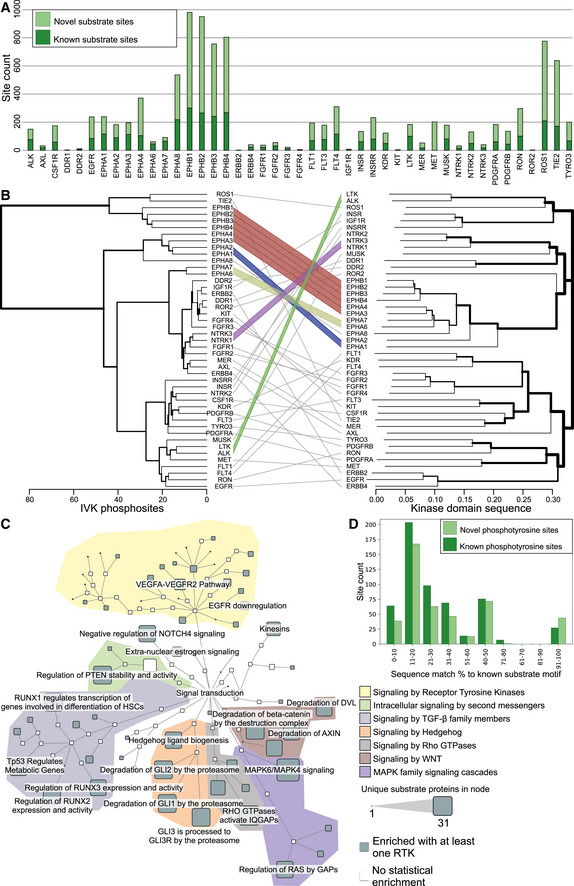
Characterization of RTK‐specific phosphotyrosine sites Number of phosphotyrosine sites identified in the IVK assay after filtering. Deeper shade of green corresponds to previously identified kinase‐substrate relationships.Dendrograms of RTK clustering based on phosphosite identifications (left) compared to Clustal Omega clustering based on protein kinase domain sequence of the same RTKs (right). Colored lines denote baits in the same order in both clustering approaches. Clustering based on phosphosites was performed using the ward.D2 method from the R stats package.Statistically enriched (q < 0.05, calculated with Fisher exact test and Benjamini–Hochberg multiple‐testing correction) reactome terms in the identified RTK substrates. Size of the node corresponds to the number of unique substrates in the node, and nodes without significant enrichment are shaded white. Only subnodes of the signal transduction root node are shown. Colored areas denote different signaling pathway trees.Substrate site amino acid sequence compared to known phosphorylation motifs from human protein reference database (Peri *et al*, 2003). Data presented represent only the receptors, for which motifs were available in the database.

A heavy‐labeled ^18^O‐ATP‐based *in vitro* kinase assay combined with LC‐MS/MS (IVK, Appendix Fig S3A) was used to characterize potential direct substrates of RTKs (Zhou *et al*, 2013; Müller *et al*, 2016). It is important to note that the kinases used in this method have access to not just their physiological molecular context but also proteins they may not normally encounter. Another important consideration is that the recombinant kinases available do not include the extracellular domains of RTKs. In total, 45 recombinant RTKs were used for experiments that included all RTK subfamilies. Of these, four kinases were missing one or more amino acids from the end of their kinase domain: NTRK3 was missing 14, FGFR1 was missing 36, DDR1 29, and EphB1 was missing one. Any phosphosites with a localization probability of under 0.75 (as assigned by MaxQuant) were filtered out, as were sites seen in any of the control experiments, where recombinant kinase was not added. This resulted in a total of 2,254 unique phosphorylated tyrosine sites, resulting in 7,758 unique kinase‐substrate interactions, or 10,194 kinase‐substrate phosphorylation site relations (Fig 5A, Dataset EV7). Of these 10,194, 6,639 were novel, and 3,555 were identified in a prior publication (Sugiyama *et al*, 2019), phosphoSitePlus, or phosphoELM. The number of identified sites varied widely between individual kinases (Fig 5B), from nearly a thousand phosphotyrosine sites (982 substrate sites for EphB1) to fewer than five sites (Fig 5C). A total of 1,027 sites were detected with only one kinase, while others had up to 37 kinases (Appendix Fig S3B). In contrast, in the control experiments without added kinase, a maximum of five phosphotyrosine sites were identified (Appendix Fig S3B inset). Based on the PhosphoSitePlus database (Hornbeck *et al*, 2015), 1,478 of the identified phosphorylation sites were previously reported, and the kinase responsible for phosphorylation was known for 124 of these sites. In 30 cases, we observed exactly the same kinase–substrate site interaction as was reported in PhosphoSitePlus (Dataset EV7).

We performed clustering analysis of the detected phosphorylation sites to obtain an overall view of the RTK substrate profile, and the result was compared with the kinase domain sequence alignment tree produced by Clustal omega (Madeira *et al*, 2019) (Fig 5B). Several kinase groups, the Ephrin receptor subfamily in particular, clustered together based on phosphosites, and most were close to their position in the kinase domain sequence‐based tree. The main difference between the two dendrograms was the Ephrin receptor subfamily in the IVK analysis, which was divided into two: one group of four receptors and one of five receptors. Substrate site‐based clustering indicated a distinction between the EphB1‐4 group and EphA1‐A8 group, while the subfamily according to the kinase domain sequence is in one well‐defined branch. The IVK analysis results were also compared with clustering results from the AP‐MS and BioID data (Appendix Fig S3C), and no strict similarity in the interactor profiles of receptors in the Ephrin subfamily was observed. This may be due to two factors. First, the number of identified phosphosites or HCIs per RTK varies, and when a few are identified, the clustering algorithm does not work. Second, substrates may also vary significantly within receptor families. However, when all three approaches (AP‐MS, BioID, and IVK) produced similarly unorganized clusters, it seems plausible that RTK substrate and interactor profiles may vary as much within subfamilies as between them. On the other hand, similarities detected between RTK substrates suggest a similarity among some functions. One such case is KDR and PDGFRB, and similarities in their IVK substrate profiles may indicate functional similarities. Indeed, the two proteins share 90 previously known interactors (Dataset EV1A) and 53 phosphosites detected in our IVK experiments, indicating a strong basis for overlapping functions.

A reactome enrichment analysis was used to link the identified RTK substrate proteins to functional networks. We focused on pathways linked to signal transduction to study the possible significance of the kinase–substrate relationships in cellular signaling networks (Fig 5C, Dataset EV8A). While signaling by RTKs was very prominent, the signaling pathways with the highest number of identified proteins were “MAPK6/MAPK4 signaling” (31 substrate proteins) and “RHO GTPases Activate Formins” (27 proteins). The most commonly enriched pathway was the “VEGFA‐VEGFR2 pathway,” which was seen with 38 of the 45 kinases used, but there were only 15 unique substrate proteins. In particular, the enrichment of the Wnt, TGF‐β. and MAPK signaling pathways may be due to a previously known link between RTKs and regulation of these three signaling pathways (Billiard *et al*, 2005; Katz *et al*, 2007; Krejci *et al*, 2012; Heldin & Moustakas, 2016; Shi & Chen, 2017). When examining the identified substrates in detail, out of the seven pathway groups emphasized in Fig 5C, TGF‐β had the highest number of substrates (Appendix Fig S3D). Our data may therefore provide further information about these pathway links.

Of the 10,194 RTK–substrate site relationships identified, 3,566 were found in one or more of the three databases used to identify known phosphorylation sites of these kinases (PhosphoSitePlus, phosphoELM; Sugiyama *et al*, 2019). A further 5 sites had identical surrounding ± 7 amino acids as in a previously identified substrate site. To further query whether the novel sites shared similarity with the previously identified, we next compared the known and novel substrate sites with known phosphorylation motifs from the human reference protein database. The motif match percentage profile between known and novel phosphorylation sites is generally of the same shape; however, we identified more perfect matches to the annotated motifs in the set of novel substrate sites (Fig 5D).

### Kinase activity‐deficient mutants reveal activity‐dependent functions

**Figure 6:**
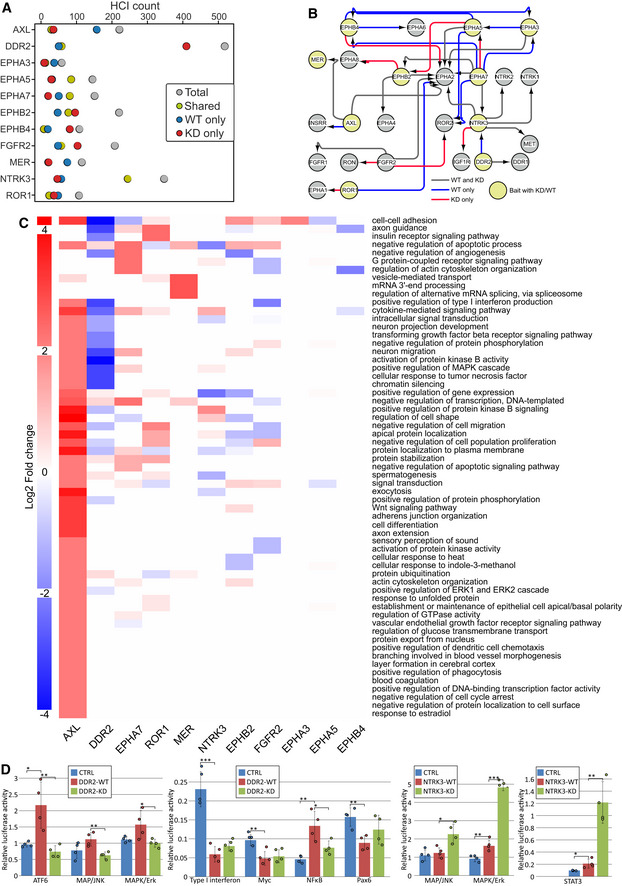
Assessment of differences in wild‐type (WT) and kinase dead RTK mutants HCI counts per WT / KD pair. Total HCI number is shown in gray, while number of shared HCI proteins is in yellow, WT only HCIs in blue, and KD only HCIs in red.Bait–bait interactions of the WT / KD baits. Shown are all RTKs found in WT/KD HCI data, but interactions are shown only for those with WT and KD constructs. Gray arrows depict preserved interactions, while blue ones are interactions that are lost in KD data, and red denotes interactions only seen in KD data.GO biological process change in KD data. Values are log2 fold change in KD compared to WT, where positive values reflect higher representation in KD data.Comparison of effects of DDR2 and NTRK3 WT and KD on activity of cellular signaling pathways. Luciferase assays were used with either WT or KD RTKs to identify transcription‐level changes caused by the lack of kinase activity of the KD mutant. *P < 0.05, **P < 0.01, ***P < 0.001; *P*‐values were calculated using t‐test. Error bars denote standard deviation, and each data point (*n* = 4, biological replicates) is shown as a separate dot.

Kinase activity‐deficient RTK mutants were used to understand which interactions might be dependent on RTK protein kinase activity. We performed AP‐MS and BioID experiments with KD mutants and compared the results to the wild‐type (WT) RTK results. The kinase domain in the mutants was deactivated with a point mutation that introduced bulk into the ATP binding pocket. The number of HCIs we identified varied widely depending on the receptor (Fig 6A, Dataset EV1A). Some WT RTKs, such as AXL, EphA7, and MER, had more HCIs than their KD counterparts, whereas in others, DDR2 in particular, the KD mutant had more HCIs. We also included one pseudokinase, ROR1. With the pseudokinase, we expected to see less differences between the KD and WT experiments. Indeed, together with EphA3, ROR1 WT and KD results were the most similar.

Considering the prominent role of RTK‐RTK interactions in the WT data (Fig 2), we first identified whether these interactions were gained or lost with the KD mutant (Fig 6B). While many interactions were lost, a similar number was also gained, suggesting that the ability of KD mutants to associate with other RTKs in general is not significantly impeded by the inability to bind ATP. However, individual RTKs such as EphA3, A5, A7, and EphB4 seem to lose many interactions with other members of the Eph subfamily. Three of these interactions were detected only by AP‐MS, two only by BioID, and two by both methods. This finding may indicate a reduced capacity of these RTKs to form heterodimers.

We then decided to sum up the lost or gained interactions by characterizing them via GOBP terms (Fig 6C). To isolate pathways that may be lost or gained by the KD mutants, we calculated fold change values for the KD experiments using WT experiments as background. The results determined which terms were proportionally better represented in KD mutant HCIs (such as cell‐cell adhesion in AXL KD) and in WT HCIs (such as cell‐cell adhesion in DDR2). These results show that although the WT AXL has more HCIs than the KD counterpart, the different proteins do not concentrate heavily on any specific GOBP annotation; hence, fewer GOBP terms are overrepresented in the WT data than in the KD HCI set.

Likewise, although the DDR2 KD mutant had a much higher number of interactors than the WT counterpart, very few pathways had a positive fold change. DDR2 is a part of the DDR subfamily of collagen receptors. The loss of cell–cell adhesion pathways in the KD mutant (Fig 6C) therefore suggests the loss of this core function. This finding together with fewer enriched pathways in general and the exceptional number of HCIs identified in BioID experiments for the DDR2 KD mutant (Dataset EV1A) indicates a proximity to a wider variety of proteins, possibly stemming from irregular cellular localization for the KD mutant.

To identify if the KD mutation had an identifiable effect on a transcription level, we next performed a luciferase assay panel measuring pathway specific activity as a response to the transfected kinase (Fig 6D, Dataset EV8B). With DDR2, where we saw the largest difference between WT and KD interactomes, we also detected significant changes in pathway activity. ATF6, MAP/JNK, MAPK/Erk, and NFKB pathways showed a significantly different response between the KD mutant and the WT kinase. In all cases, the response of the KD‐transfected cells was lower than that of WT. In contrast, with NTRK3, we saw significantly different responses in MAP/JNK, MAPK/Erk, and STAT3 pathways. However, in these cases, the WT elicited a weaker response. Together, the data from the performed luciferase assay suggests that WT DDR2 and NTRK3 may produce opposing effects on MAP/JNK and MAPK/Erk signaling pathways.

### Known roles of EGFR identified via interactome analysis

**Figure EV5:**
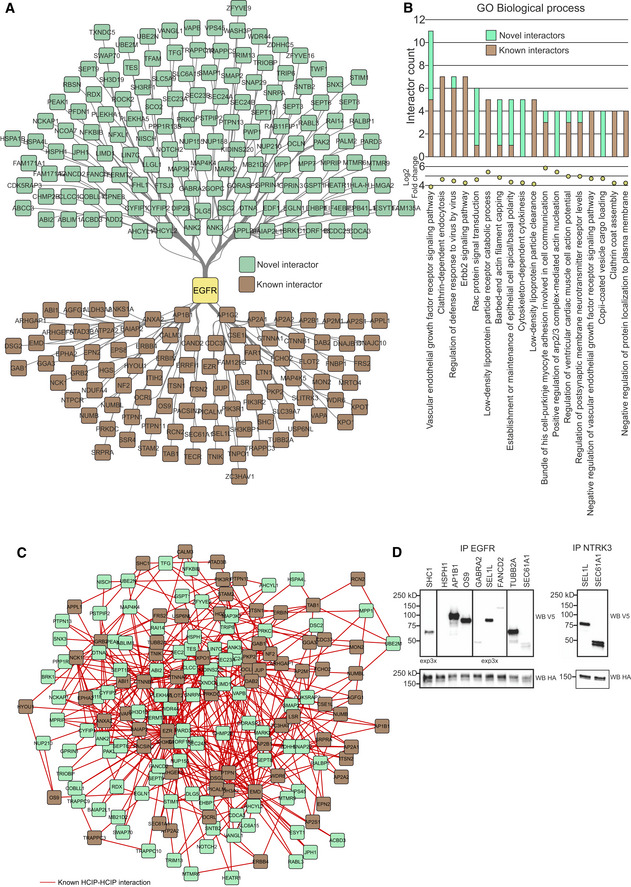
Analysis of EGFR interactome generated in this study EGFR HCIs identified in the study. Previously known interactors are denoted in brown, and novel in green.Most common significantly (q < 0.05, calculated with Fisher exact test and Benjamini–Hochberg multiple‐testing correction) enriched GO Biological process annotation terms in the EGFR HCIs. The top chart shows unique protein counts, while the lower chart depicts log2 fold change over expected value calculated from the background set.Known interactions between previously known HCIs and novel HCIs.Co‐IP validation of a subgroup of EGFR (left) and NTRK3 (right) interactors. Source data are available online for this figure.

After assessing the data produced in this study as a whole, the interactomes of singular receptors were focused on. To validate our results, we first focused on the well‐known receptor EGFR (Fig EV5). Among the EGFR HCIs, we identified 94 previously known interactors, including other kinases (e.g., EphA2 and ERBB4) and phosphatases, such as PTPN1 and PTPN11. In addition to known interactors, we identified 137 novel interactors (Fig EV5A). GOBP enrichment analysis was used to discover which processes were driven by known and novel interactors. In this set of enriched GOBP terms, the most commonly identified ones were often driven by a mixture of known and novel interactions (Fig EV5B). To see how the novel interactors relate to the known ones, we next identified the previously known interactions between the known and novel HCIPs (Fig EV5C). From these data, we could see that the novel interactors often act as bridges or network hubs between different known interactors, such as MAP3K7, LTN1, or XPO1. Furthermore, some of the novel interactors are closely related to the known ones. For example, although interaction with ABI1 is included in the combined database of previously known interactions, ABI2 was not. Similarly, VAPA is in the known interaction database, whereas VAPB is not. To validate interactions identified by our approach, we chose nine AP‐MS‐detected HCIPs at random for CO‐IP analysis. Of these, only one failed to show a clear interaction in the resulting blot (Fig EV5D, left). As two of the proteins chosen were also detected in NTRK3 AP‐MS data (SEL1L and SEC61A), we chose to further ensure the reliability of the method by performing a CO‐IP experiment targeting these two as well (Fig EV5D, right).

In the enriched biological processes (Fig EV5B), we identified terms driven only by known interactors, such as clathrin‐dependent endocytosis, terms driven by both, such as the VEGFR signaling pathway, and functions related to novel interaction partners, such as the positive regulation of ARP2/3 complex‐mediated actin nucleation. Clathrin‐mediated endocytosis of EGFR is a major active pathway of receptor internalization (Sigismund *et al*, 2008). After endocytosis, EGFR may be either recycled back to the membrane or degraded, depending on ubiquitinylation. In addition to the enriched clathrin‐dependent endocytosis identified by GOBP analysis of the EGFR interactome, we also detected multiple ubiquitinylation proteins. Six of these (CTNNB1, OS4, PRKDC, UBE2M, UBE2N, and SH3RF1) were previously documented EGFR interactors, while another four (CAND2, CDCA3, LTN1, and TRIM13) were novel interactors. Our data therefore provide additional support for the previously known interactors and molecular processes of EGFR. Furthermore, the interactome provides an additional molecular context for EGFR actions and dynamics with possible connections to novel functions.

### Characterization of the novel EphA7 interactome and phosphorylome

**Figure 7:**
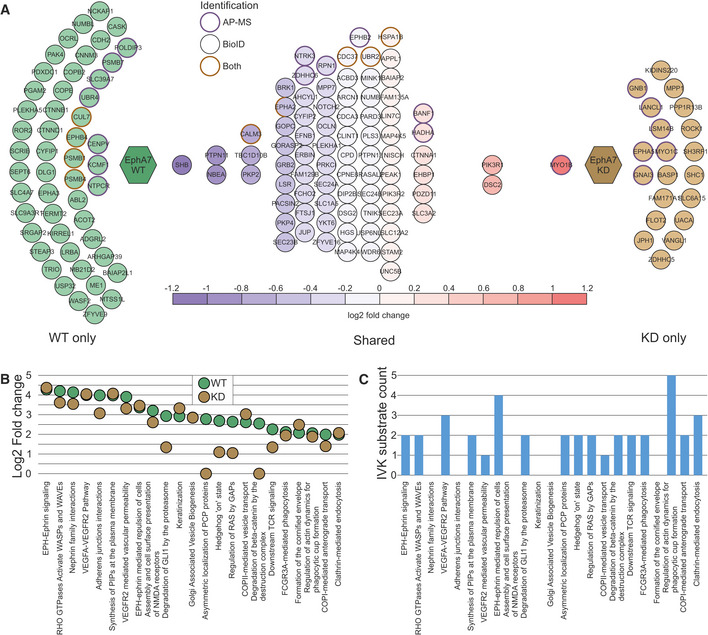
EphA7 interactome and phosphorylome analysis EphA7 WT (left) and KD (right) HCIs. Shared HCIs are in the middle arranged according to log2 fold change values. HCIs identified in AP‐MS are marked with a violet rim, BioID with black rim, and orange rim marks HCIs detected with both approaches. For the shared interactors, a bait‐normalized fold change value was calculated. Three HCIs, CDC37, UBR2, and HSPA1B, were identified in both WT and KD experiments with both AP‐MS and BioID methods. For these, the fold change values in the different experimental approaches were within 0.1 of each other, and thus the value used was an average of both. EphA2 was detected via AP‐MS and BioID with WT EphA7, and with only BioID with KD EphA7.Significantly enriched (q < 0.01, calculated with Fisher exact test and Benjamini–Hochberg multiple‐testing correction) reactome pathways in EphA7 WT data. Log2 fold change values are shown for both WT (green) and KD (orange). The KD values used did not undergo filtering to avoid eliminating smaller effects.Counts of substrate proteins identified with the IVK method in the reactome pathways enriched in EphA7 WT HCI data.

EphA7 is one of the least well‐characterized members of the Ephrin receptor subfamily, with only 12 known interactors in IntAct. We therefore more closely analyzed the identified interactions and substrates of EphA7. Although EphA7 is not expressed in HEK293 cells according to the protein atlas (Uhlén *et al*, 2015), it was seen to be endogenously expressed in the data of CellMap (Dataset EV1B, Go *et al*, 2021). WT EphA7 was analyzed together with the KD mutant to gain insights into the functions of WT EphA7 and how these functions are impacted by the loss of kinase activity (Fig 7A). We divided the interactor proteins into the following groups: WT only, KD only, and shared proteins. In total, we identified 131 HCIs for the WT protein and 101 for the KD mutant. Of the 12 previously known interactors, we detected 3 in our experiments: EphA3 was only in WT, EphA2 was in WT and KD, and GNB1 was in KD only. Although EphA2 was detected in both, in the KD experiments, it was only seen by BioID, perhaps indicating loss of heterotypic complex formation with EphA2. The formation of heterotypic complexes is a well‐documented behavior of the Eph subfamily of receptors (Janes *et al*, 2011), and given the detection of EphA5 in KD AP‐MS data only, it seems unlikely that the ability to form these complexes is completely destroyed by the KD mutation.

In the shared group, three proteins (SHB, PTPN11, and NBEA) clearly associated more with WT EphA7 than with the KD mutant, and one (MYOB1) associated more with the mutant. PTPN11 is a phosphatase with known roles in EphA2 and WNT signaling (Miao *et al*, 2000; Noda *et al*, 2016). This, together with EphA2 detection in the WT AP‐MS data, also indicates potential cooperation by these two RTKs and the loss of this function when the activity of the kinase domain is compromised. Multiple proteasomal components (PSMB1, 4, and 7) and ubiquitinylation proteins (CUL7, KCMF1, and UBR4) were only detected in WT experiments. Their presence may mean that proteasomal degradation of EphA7 is the endpoint of the receptor, as it is for some other RTKs (Jeffers *et al*, 1997; Geetha & Wooten, 2008). Moreover, the absence of these proteins in the KD data may indicate that the process is dependent on RTK kinase activity.

We next determined how the differences in HCIs affected the most enriched reactome pathways in the EphA7 data. The Ephrin signaling and VEGFA‐VEGFR2 pathways were represented by nearly identical proportions of HCIs in both the KD and WT experiments. However, differences could be seen in other pathways, especially in planar cell polarity (PCP) protein localization and various signaling events. It is possible that the KD mutation does not affect the association with proteins related to many of the signaling pathways but does affect the association with specific participants in the signaling cascades, such as the aforementioned SHB and PTPN1.

To understand how EphA7 affects the pathways it is most strongly linked to in our AP‐MS and BioID data, we combined the data with substrates identified by the IVK method and found EphA7 substrates in most of the pathways were enriched in the HCI data. Of the pathways that differed most between the WT and KD experiments, degradation of beta‐catenin by the destruction complex, degradation of GLI by proteasomes, asymmetric localization of PCP proteins, Hedgehog “on” state, and the regulation of RAS by GAPs all had identified phosphosites in the IVK data (Fig 7B and C).

Taken together, data produced by our systematic approach to identify interactors and phosphorylation targets of EphA7 suggest that the KD mutation does not hinder the association with proteins in Ephrin signaling pathways but may affect specific receptor localization, as reflected by the reduced number of proteins identified in other signaling pathways. The IVK data can additionally be used to identify specific target candidates for EphA7 in the Ephrin and VEGFA signaling pathways. Furthermore, the HCIs identified for WT EphA7 suggest that proteasomal degradation may be the termination point receptor signaling, and their absence in the KD data suggests that the process is dependent upon the kinase activity of EphA7.

## Discussion

Here, we present the comprehensive interactome and phosphorylome of human RTKs. RTKs play key roles in initiating a complex web of signaling cascades. While many have been well studied (Fig 1B), detailed and systematic knowledge of the roles and actions of a large proportion of RTKs, such as many Ephrin receptors, is lacking. Although methods such as membrane yeast two‐hybrid and mammalian membrane two‐hybrid have been applied to study facets of RTK interactions, such as RTK‐phosphatase relationships (Yao *et al*, 2017) or individual RTKs (Aboualizadeh *et al*, 2021) with success, no systemic, global mapping of interactions has previously been published. In this study, we used three complementary approaches to understand RTK functions: AP‐MS to capture stable interactions and complex stoichiometries, BioID to capture transient interactions and molecular context, and IVK to identify RTK substrates. To date, this dataset is the most comprehensive resource of RTK interactions and substrates. The data introduced here provide information about protein complexes (AP‐MS), the surrounding molecular landscape (BioID), and signaling activity (IVK). Overall, these three approaches can be used to characterize and introduce additional context for well‐known receptors (Fig EV5), discover the functions of less well‐known receptors (Fig 7), and identify possible active roles for RTKs in signaling networks via substrate information (Fig 5). The data supplement the scarce information available for some RTKs, and for the whole kinase family, these data underscore the interactions within and across subfamilies. While the interconnectedness of RTK signaling networks is a well‐known feature of these receptors (Kholodenko *et al*, 2010; Paul & Hristova, 2019), the data presented in this study supply additional molecular context for the signaling networks and indicate probable avenues of information flow. The interactomics insights gained here highlight the role of RTKs as important intersections in an increasingly complicated landscape of cellular signaling networks.

Despite the comprehensiveness of the results presented here, our model does have several limitations inherent to large‐scale high‐throughput proteomic studies. The results might not capture all cell type and context‐dependent interactions. Our use of pervanadate to ensure the capture of active‐state interactions does alter the specific molecular landscape of the cells, and thus, the detected interactions do not necessarily reflect in vivo activation of RTKs. Furthermore, the isoforms expressed in various cell populations may differ from the isoforms used here. Indeed, not all RTKs are physiologically expressed in HEK293 cells (Dataset EV1B), nor does this cell line represent all common cell types. Therefore, especially interactors differentially expressed or especially exclusively expressed in more specialized cell lines may be left out. Likewise, it is possible that some interactions may be disturbed by the C‐terminal tag of the RTKs. Similarly, we cannot be certain that the RTKs localize to the correct plasma membrane subdomains, as systemic information about this detail of RTK behavior is not available. Future improvements on understanding RTK behavior as a part of a more complete model system might require both more information about the specific RTK membrane substructure localization and perhaps using 3D organoid cell culture techniques to better imitate the 3D tissue structure, within which RTKs physiologically function.

The BioID results presented describe RTK interactomes over the course of 24 h prior to activation. Although some important RTK interactions are identified only with pervanadate‐activated receptors (as opposed to untreated samples, Fig EV3), the majority of the identifications can be proteins the receptors encounter prior to activation. The experiment carried out with ultraID, which can biotinylated interactors in as little as 10 min, shows that the RTK activation by the addition of pervanadate enhances the identification of critical RTK interactors, such as PLCG1. While it would be possible to study only the interactions of activated receptors via, for example, turboID (Branon *et al*, 2018) or UltraID (preprint: Zhao *et al*, 2021), which allows efficient biotinylation of interacting proteins in minutes, currently there is no combination tag for AP‐MS and turboID comparable to the MAC‐tag. While we sought to validate many of the direct interactions via Co‐IP (Figs EV1E and EV2B), validation of BioID results was not within the scope of the project. The same applies for functional impacts of the identified interactions—although we can detect differences on a pathway activation level between WT and KD RTKs (Fig 6D), larger‐scale investigation of signaling effects of interactors and substrates was not considered. The IVK method has the caveat of using recombinant RTKs and giving each kinase access to more than their physiological molecular context. Although nuclear proteins are not specifically solubilized, substrates available in this method may cover, for example, membrane domains or structures from which RTKs are normally excluded.

In summary, the study describes the RTK molecular context and interactomics landscape, as seen from the perspective of AP‐MS and BioID methodology, and the phosphorylome as identified by *in vitro* kinase assays. The results are, to our knowledge, thus far the most comprehensive data resource in RTK interactomics and substrates. The combined knowledge of the multifaceted dataset presented may best be used as a potential pool for each RTK and be combined with additional application‐specific information, such as data on specific cancer types or drug applications, to generate testable hypotheses of molecular systems surrounding RTKs. The data may also be used to gain insight and context into known functions of well‐studied kinases, such as EGFR (Fig EV5), or to derive indications of possible roles for less well‐known RTKs, such as EphA7 (Fig 7). Furthermore, systemic insights can be gained by studying the connections within groups of receptors, of which we chose EphA5‐A8 as an example subgroup (Appendix Fig S2). The knowledge presented herein emphasizes common functions between RTKs and the landscape that they share with other signaling pathways. The three perspectives of the data presented here, stable interactions (AP‐MS), proximal and transient interactions (BioID), and kinase–substrate relationships (IVK), together form a comprehensive molecular environment that can serve as a foundation for a systemic view of RTK signaling pathways and networks.

## Materials and Methods

### Reagents and Tools table

**Table:** 

Reagent/Resource	Reference or Source	Identifier or Catalog number
**Experimental models**		
Human: HEK 293 cell line	ATCC	Cat# CRL‐1573
Human: HEK Flp‐In T‐REx 293 cell line	Thermo Fisher Scientific	Cat# R78007
**Recombinant DNA**		
ATF2 reporter vector	Qiagen	Cat# CCA‐901L
Gateway™ pDONR221™	Thermo Fisher Scientific	12536017
MAC‐GFP	Liu *et al* (2018)	Addgene, plasmid no. 139636
MAC‐tag‐C destination vector	Liu *et al* (2018)	Addgene, plasmid no. 108077
pOG44 Flp‐Recombinase expression vector	Thermo Fisher Scientific	Cat# V600520
Human ALK gateway entry clone	University of Helsinki genome biology unit	Orfeome collection 100061564
Human AXL gateway entry clone	Varjosalo *et al* (2008)	N/A
Human AXL KD gateway entry clone	Varjosalo *et al* (2008)	N/A
Human CSF1R gateway entry clone	Varjosalo *et al* (2008)	N/A
Human DDR1 gateway entry clone	Johannessen *et al* (2010)	Addgene, plasmid no 23910
Human DDR2 gateway entry clone	Varjosalo *et al* (2008)	N/A
Human DDR2 KD gateway entry clone	Varjosalo *et al* (2008)	N/A
Human EGFR gateway entry clone	Varjosalo *et al* (2008)	N/A
Human EphA1 gateway entry clone	Varjosalo *et al* (2008)	N/A
Human EphA10 gateway entry clone	Orfeome collection 5.1	ORF ID 14424
Human EphA2 gateway entry clone	Varjosalo *et al* (2008)	N/A
Human EphA3 gateway entry clone	Varjosalo *et al* (2008)	N/A
Human EphA3 KD gateway entry clone	Varjosalo *et al* (2008)	N/A
Human EphA4 gateway entry clone	Varjosalo *et al* (2008)	N/A
Human EphA5 gateway entry clone	Varjosalo *et al* (2008)	N/A
Human EphA5 KD gateway entry clone	Varjosalo *et al* (2008)	N/A
Human EphA6 gateway entry clone	University of Helsinki genome biology unit	Orfeome collection 100058877
Human EphA7 gateway entry clone	Varjosalo *et al* (2008)	N/A
Human EphA7 KD gateway entry clone	Varjosalo *et al* (2008)	N/A
Human EphA8 gateway entry clone	University of Helsinki genome biology unit	Orfeome collection 100014738
Human EphB2 gateway entry clone	Varjosalo *et al* (2008)	N/A
Human EphB2 KD gateway entry clone	Varjosalo *et al* (2008)	N/A
Human EphB4 gateway entry clone	Varjosalo *et al* (2008)	N/A
Human EphB4 KD gateway entry clone	Varjosalo *et al* (2008)	N/A
Human EphB6 gateway entry clone	Orfeome collection 5.1	ORF ID 52951
Human ErbB2 gateway entry clone	University of Helsinki genome biology unit	Orfeome collection 100058794
Human ErbB3 gateway entry clone	Varjosalo *et al* (2008)	N/A
Human FGFR1 gateway entry clone	University of Helsinki genome biology unit	Orfeome collection 100009459
Human FGFR2 gateway entry clone	Varjosalo *et al* (2008)	N/A
Human FGFR2 KD gateway entry clone	Varjosalo *et al* (2008)	N/A
Human FGFR3 gateway entry clone	University of Helsinki genome biology unit	Orfeome collection 100066410
Human FGFR4 gateway entry clone	University of Helsinki genome biology unit	Orfeome collection 100010808
Human FLT1 gateway entry clone	Varjosalo *et al* (2008)	N/A
Human FLT3 gateway entry clone	Varjosalo *et al* (2008)	N/A
Human FLT4 gateway entry clone	University of Helsinki genome biology unit	Orfeome collection 100068206
Human IGF1R gateway entry clone	University of Helsinki genome biology unit	Orfeome collection 100009391
Human INSR gateway entry clone	Varjosalo *et al* (2008)	N/A
Human INSRR gateway entry clone	University of Helsinki genome biology unit	Orfeome collection 100062381
Human KDR gateway entry clone	Orfeome collection 5.1	ORF ID 56932
Human LMR1 gateway entry clone	Varjosalo *et al* (2008)	N/A
Human LMR2 gateway entry clone	Johannessen *et al* (2010)	Addgene, plasmid no. 23914
Human LMR3 gateway entry clone	GeneScript	Synthesized plasmid
Human LTK gateway entry clone	GeneScript	Synthesized plasmid
Human MER gateway entry clone	Johannessen *et al* (2010)	Addgene, plasmid no. 23900
Human MER KD gateway entry clone	Varjosalo *et al* (2008)	N/A
Human MET gateway entry clone	Varjosalo *et al* (2008)	N/A
Human MUSK gateway entry clone	Orfeome collection 5.1	ORF ID 53052
Human NTRK1 gateway entry clone	Johannessen *et al* (2010)	Addgene, plasmid no. 23891
Human NTRK2 gateway entry clone	Johannessen *et al* (2010)	Addgene, plasmid no. 23883
Human NTRK3 gateway entry clone	Varjosalo *et al* (2008)	N/A
Human NTRK3 KD gateway entry clone	Varjosalo *et al* (2008)	N/A
Human PDGFRA gateway entry clone	Johannessen *et al* (2010)	Addgene, plasmid no. 23892
Human PDGFRB gateway entry clone	University of Helsinki genome biology unit	Orfeome collection 100011461
Human RET gateway entry clone	Johannessen *et al* (2010)	Addgene, plasmid no. 23906
Human RON gateway entry clone	Varjosalo *et al* (2008)	N/A
Human ROR1 gateway entry clone	Varjosalo *et al* (2008)	N/A
Human ROR1 KD gateway entry clone	Varjosalo *et al* (2008)	N/A
Human ROR2 gateway entry clone	Varjosalo *et al* (2008)	N/A
Human ROS1 gateway entry clone	University of Helsinki genome biology unit	Orfeome collection 100066413
Human RYK gateway entry clone	University of Helsinki genome biology unit	Orfeome collection 100015603
Human STYK1 gateway entry clone	University of Helsinki genome biology unit	Orfeome collection 100001903
Human TEK gateway entry clone	University of Helsinki genome biology unit	Orfeome collection 100011460
Human TYRO3 gateway entry clone	Varjosalo *et al* (2008)	N/A
**Antibodies**		
Alexa Fluor488‐conjugated secondary antibody	Thermo Fisher Scientific	Cat# A‐11001
Goat anti‐mouse IgG H&L (HRP)	Abcam	97023
Anti‐V5 antibody	Invitrogen	37‐7500
Mouse monoclonal anti‐HA tag	Thermo Fisher Scientific	Cat# 26183
**Chemicals, enzymes and other reagents**		
Amersham™ ECL™ Prime	Cytiva	RPN2232
Ammonium bicarbonate (AMBIC)	Sigma‐Aldrich	1066‐33‐7
Benzonase® Nuclease	Santa Cruz Biotechnology	sc‐202391
Bio‐Spin® Chromatography Columns	Bio‐Rad	732‐6008
Biotin	Thermo Fisher Scientific	29129
Bradford reagent	Bio‐Rad	500‐0006
DAPI	Sigma‐Aldrich	Cat# D9542
DMEM	Life Technologies	41965062
ECL Western Blotting Detection Reagent	GE Healthcare	RPN2209
Fetal bovine serum (FBS)	Gibco	10270‐106
Formic Acid, ≥ 95%	Sigma	64‐18‐6
FSBA	Sigma‐Aldrich	Cat# F9128
FuGENE 6 transfection reagent	Promega	Cat# E2691
Gateway™ LR Clonase™ Enzyme Mix	Life Technologies	11791043
HEPES	Sigma	7365‐45‐9
Hygromycin B	Thermo Fisher Scientific	Cat# 10687‐010
IGEPAL (electrophoresis reagent) CA630	Sigma	9002‐93‐1
Iodoacetamide (IAA)	Sigma‐Aldrich	64‐69‐7
Laemmli sample buffer	Bio‐Rad	1610737
Mouse monoclonal Anti‐HA−Agarose conjugated beads	Sigma‐Aldrich	Cat# A2095
Mowiol 4‐88	Sigma‐Aldrich	Cat# 81381
Penicillin–streptomycin	Life Technologies	15140130
Phenylmethanesulfonylfluoride (PMSF) > 98.5%	Sigma	329‐98‐6
Pierce™ BCA Protein assay	Thermo Fisher Scientific	23225
Protease Inhibitor cocktail	Sigma‐Aldrich	Cat# P8340
Pure nitrocellulose membrane 0.45 µm	Perkin‐Elmer	NBA085C001EA
Restore Plus Stripping buffer	Thermo Fisher	46430
SDS‐PAGE gel	Bio‐Rad	4561096
Sequencing grade trypsin	Promega	Cat# V5113
Skimmed milk powder	Valio	D1‐5824
Sodium chloride	Merck	7647‐14‐5
Sodium dodecyl sulfate	Sigma	151‐21‐3
Sodium fluoride	Sigma	7681‐49‐4
Strep‐Tactin® Sepharose® 50% (vol/vol) suspension	IBA life sciences	2‐1201‐010
Tetracycline hydrochloride	Sigma‐Aldrich	Cat# T‐3383
Tris(2‐carboxyethyl)phosphine (TCEP)	Sigma‐Aldrich	51805‐45‐9
Triton X‐100	Sigma	X100‐500
Trypsin‐EDTA	Gibco	25200‐56
TWEEN®20	Sigma‐Aldrich	P1379‐250ML
Water MS‐grade	Merck	7732‐18‐5
γ[18O4]‐ATP	Cambridge Isotope Laboratory	Cat# OLM‐7858‐PK
Recombinant human ALK	Thermo Fisher Scientific	Cat# PV3867
Recombinant human AXL	Thermo Fisher Scientific	Cat# PV3971
Recombinant human CSF1R	Thermo Fisher Scientific	Cat# PV3249
Recombinant human DDR1	Thermo Fisher Scientific	Cat# PV6047
Recombinant human DDR2	Thermo Fisher Scientific	Cat# PV4188
Recombinant human EGFR	Thermo Fisher Scientific	Cat# PV3872
Recombinant human EPHA1	Thermo Fisher Scientific	Cat# PV3841
Recombinant human EPHA2	Thermo Fisher Scientific	Cat# PV3688
Recombinant human EPHA3	Thermo Fisher Scientific	Cat# PV3359
Recombinant human EPHA4	Thermo Fisher Scientific	Cat# PV3651
Recombinant human EPHA6	Thermo Fisher Scientific	Cat# PV6339
Recombinant human EPHA7	Thermo Fisher Scientific	Cat# PV3689
Recombinant human EPHA8	Thermo Fisher Scientific	Cat# PV3844
Recombinant human EPHB1	Thermo Fisher Scientific	Cat# PV3786
Recombinant human EPHB2	Thermo Fisher Scientific	Cat# PV3625
Recombinant human EPHB3	Thermo Fisher Scientific	Cat# PV3658
Recombinant human EPHB4	Thermo Fisher Scientific	Cat# PV3251
Recombinant human ERBB2	Thermo Fisher Scientific	Cat# PV3366
Recombinant human ERBB4	Thermo Fisher Scientific	Cat# PV3626
Recombinant human FGFR1	Thermo Fisher Scientific	Cat# PV3146
Recombinant human FGFR2	Thermo Fisher Scientific	Cat# PV3368
Recombinant human FGFR3	Thermo Fisher Scientific	Cat# PV3145
Recombinant human FGFR4	Thermo Fisher Scientific	Cat# P3054
Recombinant human FLT1	Thermo Fisher Scientific	Cat# PV3666
Recombinant human FLT3	Thermo Fisher Scientific	Cat# PV3182
Recombinant human FLT4	Thermo Fisher Scientific	Cat# PV4129
Recombinant human IGF1R	Thermo Fisher Scientific	Cat# PV3250
Recombinant human INSR	Thermo Fisher Scientific	Cat# PV3781
Recombinant human INSRR	Thermo Fisher Scientific	Cat# PV4111
Recombinant human KDR	Thermo Fisher Scientific	Cat# PV3660
Recombinant human KIT	Thermo Fisher Scientific	Cat# PV3589
Recombinant human LTK	Thermo Fisher Scientific	Cat# PV4651
Recombinant human MERTK	Thermo Fisher Scientific	Cat# PV3627
Recombinant human MET	Thermo Fisher Scientific	Cat# PV3143
Recombinant human MUSK	Thermo Fisher Scientific	Cat# PV3834
Recombinant human NTRK1	Thermo Fisher Scientific	Cat# PV3144
Recombinant human NTRK2	Thermo Fisher Scientific	Cat# PV3616
Recombinant human NTRK3	Thermo Fisher Scientific	Cat# PV3617
Recombinant human PDGFRA	Thermo Fisher Scientific	Cat# PV3811
Recombinant human PDGFRB	Thermo Fisher Scientific	Cat# P3082
Recombinant human RON	Thermo Fisher Scientific	Cat# PV4314
Recombinant human ROR2	Thermo Fisher Scientific	Cat# PV3861
Recombinant human ROS1	Thermo Fisher Scientific	Cat# PV3814
Recombinant human TIE2	Thermo Fisher Scientific	Cat# PV3628
Recombinant human TYRO3	Thermo Fisher Scientific	Cat# PV3828
Recombinant Human EGF	R&D systems	Cat# 236‐EG‐200
Recombinant Human FGF basic	R&D systems	Cat# 3718‐FB‐025
Recombinant Human GDNF	R&D systems	Cat# 212‐GD‐010/CF
Recombinant Human HGF	R&D systems	Cat# 294‐HG‐005/CF
Recombinant Human IGF‐I	R&D systems	Cat# 291‐G1‐200
Recombinant Human NT‐3	R&D systems	Cat# 267‐N3‐005/CF
Recombinant Human PDGF‐BB	R&D systems	Cat# 220‐BB‐010
Recombinant Human VEGF	R&D systems	Cat# 293‐VE‐010/CF
**Software**		
CRAPome v1	Mellacheruvu *et al* (2013)	http://www.crapome.org/
Cytoscape version 3.7	Shannon *et al* (2003)	http://www.cytoscape.org/
ImageJ	MacBiophotonics	https://imagej.nih.gov/ij/
MaxQuant version 1.6.4.3	Cox and Mann (2008)	http://www.biochem.mpg.de/5111795/maxquant
Progenesis LC‐MS version 4.0	Nonlinear Dynamics	http://www.nonlinear.com/progenesis/qi/
Proteome Discoverer version 1.4	Thermo Fisher Scientific	https://www.Thermo Fisher.com/fi/en/home.html
SAINTexpress version 3.6	Choi *et al* (2011)	http://saint‐apms.sourceforge.net/Main.html
Xcalibur version 2.7.0	Thermo Fisher Scientific	https://www.Thermo Fisher.com/fi/en/home.html
Fragpipe version 17	Nesvizhskii Lab	https://fragpipe.nesvilab.org/
**Other**		
Amersham ECL prime western blotting detection reagent kit	GE Healthcare	Cat# RPN2232
BCA protein assay kit	Thermo Fisher Scientific	Cat# 23225
Cignal 45‐Pathway Reporter Array	Qiagen	Cat# 336841
Dual‐Luciferase reporter assay system	Promega	Cat# E1960
Gateway LR Clonase Enzyme Mix	Life Technologies	Cat # 11791043
Bio‐Spin Chromatography Columns	Bio‐Rad	Cat# 732‐6008
C18 reversed‐phase spin columns	Nest Group	Cat# SEM SS18V
C18 macrospin columns	Nest Group	Cat# SMM SS18V
Q Exactive™ Hybrid Quadrupole‐Orbitrap™ Mass Spectrometer	Thermo Fisher Scientific	
EASY‐nLC 1000	Thermo Fisher Scientific	
Bio‐Dot® Microfiltration System	Bio‐Rad	1703938
Evosep One	Evosep	EV‐1000
Hybrid trapped ion mobility quadrupole TOF mass spectrometer	Bruker	TimsTOF Pro
Electrospray ionization sprayer	Thermo Fisher	
Fluorescence microscope	Leica	Leica TCS SP8 STED

### Methods and Protocols

#### RTK constructs

RTK constructs were obtained from three sources: 7 were gifts from William Hahn & David Root (Addgene plasmid #23914, 23906, 23900, 23910, 23892, 23883, 23891) (Johannessen *et al*, 2010), 15 were from ORFeome collection (ORFeome and MGC Libraries; Genome Biology Unit supported by HiLIFE and the Faculty of Medicine, University of Helsinki, and Biocenter Finland), 29 from a collection published previously (Varjosalo *et al*, 2013a), and 2 were synthesized by Genscript. RTKs were cloned into MAC‐TAG‐C expression vectors (Liu *et al*, 2018) and pcDNA™‐DEST40 (Thermo Fisher Scientific) via gateway cloning.

### Generation of UltraID‐tag Gateway^®^ destination vectors

To generate Gateway compatible UltraID (Zhao *et al*, 2021) destination vectors, plasmids containing the tags (C‐terminal: StrepIII/HA/UltraID, N terminal: UltraID/HA/StrepIII) were synthesized by Genescript. These were digested with restriction enzymes and inserted into MAC‐tagged (Liu *et al*, 2020) vector in which the entire MAC‐tag was removed.

### Cell culture

For the generation of stable cell lines inducibly expressing the MAC‐tagged RTK baits, Flp‐In 293T‐REx cell lines (cultured in DMEM (4.5 g/l glucose, 2 mM L‐glutamine) supplemented with 50 mg/ml penicillin, 50 mg/ml streptomycin, and 10% fetal bovine serum (FBS)) were co‐transfected with the expression RTK vector, and pOG44 vector (Invitrogen) using FuGENE 6 transfection reagent. Cell lines were obtained directly from commercial sources; additionally, only low passage cells (passage number < 10) were used for experiments. Manufacturers are known to follow the authentication of cell line batches regularly, and certificates of authentication were provided with the cells and checked before their use. Cells were selected with 50 ml/ml streptomycin and 100 μg/ml hygromycin for two weeks, starting two days after transfection. Positive clones were pooled and amplified in 150 mm plates. Each cell line was expanded to 80% confluence in 20 × 150 mm plates. Ten of the plates were used for the AP‐MS approach and 10 for BioID. For AP‐MS, expression of the bait protein was induced with 1 μg/ml tetracycline 24 h prior to harvesting. With BioID, 50 μM biotin was added to the plates in addition at induction with tetracycline. Pervanadate treatment was performed at a concentration of 100 µM for 15 min prior to harvesting. Cells from 5 × 150 mm fully confluent plates (~ 5 × 10^7^ cells) were harvested on ice and pelleted as one biological sample; thus, each bait protein had two biological replicates for each of the two approaches. Samples were snap‐frozen and stored at −80°C. Tetracycline concentration of 1 μg/ml was used to produce expression levels corresponding to expression levels similar to endogenous (Glatter *et al*, 2009; Varjosalo *et al*, 2013a, 2013b; Yadav *et al*, 2017).

For ligand experiments, the cells were treated with the ligand of the expressed RTK (EGF 10 ng/ml; FGF 10 ng/ml; IGF 20 ng/ml; HGF 50 ng/ml; NT‐3 10 ng/ml; PDGF‐BB 10 ng/ml; GDNF 10 ng/ml; all from R&D systems (see the Reagents and Tools table for catalog numbers)). Ligand treatment was started at the time of tetracycline induction, 24 h before harvesting. Ligand experiments were carried out for 8 RTKs (EGFR, FGFR1, FGFR4, IGF1R, INSR, INSRR, PDGFRB, and RET).

For the NTRK3 ultraID experiment, the stable Flp‐In™ 293T‐REx cell line inducibly expressing the UltraID‐tagged NTRK3 (cultured in DMEM (4.5 g/l glucose, 2 mM L‐glutamine) supplemented with 10% FBS, 50 mg/ml penicillin, 50 mg/ml streptomycin) was co‐transfected with the expression vector and the pOG44 vector (Invitrogen) using the Fugene6 transfection reagent (Roche Applied Science). Two days after transfection, cells were selected in 50 mg/ml streptomycin and hygromycin (100 μg/ml) for 2 weeks, and then the positive clones were pooled and amplified. Stable cells expressing UltraID‐tag fused to the green fluorescent protein (GFP) were used as negative controls and processed in parallel with the NTRK3 line. Each stable cell line was expanded to 80% confluence in 150 mm cell culture plates. Five plates were used as one biological replicate and tetracycline was added at a concentration of 1 μg/ml for 24 h for bait protein expression. For biotin treatment, pervanadate was added for 15 min before harvesting, and 50 μM biotin was added for 10 mins before harvesting. Thus, each bait protein had three biological replicates in two different conditions. Samples were snap‐frozen and stored at −80°C.

### Affinity purification of RTK interactors

After harvesting, all samples were assigned a running identifier number for blinding instead of the gene/protein name of each bait. The name was then restored only once data analysis began. AP‐MS and BioID purifications were carried out as outlined in the MAC‐tag workflow publication (Liu *et al*, 2020). For AP‐MS, samples were lysed in 3 ml of ice‐cold lysis buffer 1 (0.5% IGEPAL, 50 mM Hepes (pH 8.0), 150 mM NaCl, 50 mM NaF, 1.5 mM NaVo_3_, 5 mM EDTA, with 0.5 mM PMSF, and protease inhibitors (Sigma‐Aldrich)). For BioID approach, cell pellets were thawed in 3 ml of ice‐cold lysis buffer 2 (0.5% IGEPAL, 50 mM Hepes (pH 8.0), 150 mM NaCl, 50 mM NaF, 1.5 mM NaVO_3_, 5 mM EDTA, 0.1% SDS, with 0.5 mM PMSF, and protease inhibitors (Sigma‐Aldrich)). Lysates were sonicated and treated with benzonase (Bio‐Rad).

Lysates were centrifuged at 16,000 *g* for 15 min, after which the supernatant was centrifuged for an additional 10 min to obtain cleared lysate. This was then loaded consecutively on spin columns (Bio‐Rad) containing 200 μl Step‐Tactin beads (IBA, GmbH) prewashed with lysis buffer 1. The beads were washed thrice with 1 ml of lysis buffer 1, and 4 × 1 ml of wash buffer (50 mM Tris–HCl, pH 8.0, 150 mM NaCl, 50 mM NaF, 5 mM EDTA). After the final wash, beads were resuspended in 2 × 300 μl elution buffer (50 mM Tris–HCl, pH 8.0, 150 mM NaCl, 50 mM NaF, 5 mM EDTA, 0.5 mM biotin) for 5 min, and eluates were collected into 2 ml tubes. Cysteine bonds were then reduced with 5 mM Tris(2‐carboxyethyl) phosphine (TCEP) for 30 min at 37°C, followed by alkylation with 10 mM iodoacetamide for 20 min in the dark. Proteins were then digested to peptides with sequencing grade modified trypsin (Promega V5113), at 37°C overnight.

The following day quenching was done with 10% TFA, and the samples were desalted with C18 reversed‐phase spin columns. These columns were first washed 3 × 100 μl of 100% acetonitrile (ACN), and equilibrated with 3 × 100 μl of buffer A (0.1% TFA, 1% ACN). This was followed by 4 × 100 μl of wash buffer (0.1% TFA, 5% ACN). Peptide samples were then loaded 300 μl at a time, followed by 4 × 100 μl washes with the wash buffer. Elution was done with 3 × 100 μl of elution buffer (0.1% TFA, 50% ACN). The eluted peptide sample was then dried in a vacuum centrifuge and reconstituted to a final volume of 30 μl in buffer A.

### *In* *vitro* kinase assay

HEK 293 cells were cultivated in DMEM (GE Healthcare), supplemented with 10% FBS and antibiotics (penicillin, 50 μg/ml and streptomycin, 100 μg/ml). Cells on a plate were washed with PBS, dislodged with PBS and EDTA, and collected with centrifugation at 1,400 *g* for 5 min before lysis. Cell lysate was prepared by lysing the pelleted cells with buffer containing 50 mM Tris–HCL, pH 7.5, 150 mM NaCl, 5 mM EDTA, 1% NP‐40 (Invitrogen, Thermo Fisher Scientific), and protease inhibitors cocktail (Sigma) on ice. The cell debris was cleared by centrifugation at 16,000 *g* for 10 min. The protein contents were measured using a BCA protein assay kit (Pierce, Thermo Scientific) and the cell fractions were stored at −80°C.

Cell fractions were thawed on ice and endogenous kinases were inhibited with 5′‐[p‐(fluorosulfonyl)benzoyl]adenosine (FSBA; Sigma‐Aldrich) in DMSO at a final concentration of 1 mM FSBA and 10% DMSO in Tris–HCL, pH 7.5 for 1 h at 30°C. Excess FSBA reagent was removed by ultracentrifugation with 15 ml 10 K MWCO Amicon^®^ Ultra‐4 centrifugal filter units (Merck) at 3,500 *g* at RT. Proteins were washed 4× the initial volume with a kinase assay buffer (50 mM Tris–HCl, pH 7.5, 10 mM MgCl2, 1 mM DTT), adjusted to 2 mg/ml, and stored on ice. For kinase reaction, 200 μg (100 μl) of FSBA‐treated cell lysate was incubated with 1 μg of kinase (see the reagents and tools table for catalog numbers) and 1 mM γ[^18^O_4_]‐ATP (Cambridge Isotope Laboratory) in 30°C for 1 h. For negative control experiments, 200 μg of FSBA‐treated cell lysate was incubated with 1 mM γ[^18^O_4_]‐ATP in the absence of added kinase. Reactions were halted with 100 μl of 8 M urea.

Prior to digestion, the protein samples were reduced with 5 mM Tris(2‐carboxyethyl) phosphine (TCEP; Sigma‐Aldrich) for 20 min at 37°C, and then alkylated with 10 mM iodoacetamide (IAA; Sigma‐Aldrich) for 20 min at room temperature in the dark. 600 μl of ammonium bicarbonate (AMBIC; Sigma‐Aldrich) was added to dilute urea before trypsin digestion. Sequencing Grade Modified Trypsin (Promega) was then used to get a 1:100 enzyme:substrate ratio and the samples were incubated overnight at 37°C. After digestion, the samples were desalted with C18 macrospin columns (Nest Group).

The macrospin columns were first conditioned by centrifuging 200 μl of 100% ACN through at 55 *g*, followed by 200 μl of water. Column was then equilibrated twice with 200 μl of buffer A (0.1% TFA, 1% ACN). Sample was then added 100 μl at a time, and washed twice with 200 μl of buffer A. Finally, the sample was released with 3 × 200 μl of elution buffer (80% ACN, 0.1% TFA).

Phosphopeptide enrichment was performed using immobilized metal ion affinity chromatography with titanium (IV) ion (Ti4+‐IMAC). The IMAC material was prepared by following the steps of the protocol published previously (Zhou *et al*, 2013). For enrichment of phosphopeptides, the Ti4+‐IMAC beads were loaded onto GELoader tips (Thermo Fisher Scientific). The material was then conditioned with 50 μl of conditioning buffer (50% CH_3_CN, 6% TFA) by centrifuging at 150 g until all of the buffer had gone through. The protein digests were dissolved in a loading buffer (80% CH_3_CN, 6% trifluoroacetic acid (TFA)) and added into the spin tips and centrifuged at 150 g until all had gone through. The columns were then washed with 50 μl of wash buffer 1 (50% CH_3_CN, 0.5% TFA, 200 mM NaCl), followed by 50 μl of wash buffer 2 (50% CH_3_CN, 0.1% TFA), and finally the bound phosphopeptides were eluted with 10% ammonia, followed by a second elution with elution buffer (80% CH_3_CN, 2% FA). Samples were then dried in a vacuum centrifuge and reconstituted to a final volume of 15 μl in 0.1% TFA and 1% CH_3_CN.

### Liquid chromatography‐mass spectrometry (LC‐MS)

The LC‐MS/MS analysis was performed on Q‐Exactive or Orbitrap Elite mass spectrometers using Xcalibur version 3.0.63 with an EASY‐nLC 1000 system attached via electrospray ionization sprayer (Thermo Fisher Scientific). For each sample, two biological replicates were used. Peptides were eluted and separated with C‐18‐packed precolumn and an analytical column, using a 60‐min buffer gradient from 5 to 35% buffer B, followed by 5‐min gradient from 35 to 80% buffer B, and a 10‐min gradient from 80 to 100% buffer B at a flow rate of 300 nl/min (Buffer A: 0.1% formic acid in 2% acetonitrile and 98% HPLC‐grade water; buffer B: 0.1% formic acid in 98% acetonitrile and 2% HPLC‐grade water). Four microliters of peptide samples were loaded for each analysis from an enclosed, cooled autosampler. Data‐dependent FTMS acquisition was in positive‐ion mode for 80 min, and a full scan from 200 to 2,000 m/z with a resolution of 70,000 was performed, followed by top 10 CID‐MS2 ion trap scans with a resolution of 17,500. Dynamic exclusion was set to 30 s.

The Acquired MS2 spectral data files (Thermo RAW) were searched with Proteome Discoverer 1.4 (Thermo Scientific) using SEQUEST search engine against human protein database extracted from UniProtKB (https://uniprot.org) on March 26, 2019. For the searches, trypsin was set as the digestion enzyme with a maximum of two missed cleavages permitted. Precursor mass tolerance was set to ± 15 ppm, and fragment mass tolerance to 0.05 Da. Carbamidomethylation of cysteine was defined as a static modification, and oxidation of methionine and biotinylation of lysine and N‐termini as variable modifications.

The ultraID samples were analyzed using the Evosep One liquid chromatography system coupled to a hybrid trapped ion mobility quadrupole TOF mass spectrometer (Bruker timsTOF Pro) via a CaptiveSpray nano‐electrospray ion source. An 8 cm × 150 µm column with 1.5 µm C18 beads (EV1109, Evosep) was used for peptide separation with the 60 samples per day methods (buffer A: 0.1% formic acid in water; buffer B: 0.1% formic acid in acetonitrile). The MS analysis was performed in the positive‐ion mode using data‐dependent acquisition (DDA) in PASEF mode with 10 PASEF scans per topN acquisition cycle. Raw data (.d) acquired in PASEF (Meier *et al*, 2018) mode were processed with MSFragger (Yu *et al*, 2020) against the human protein database extracted from UniProtKB. Both instrument and label‐free quantification parameters were left to default settings.

For the kinase assays, LC‐MS/MS analysis was performed as before, except peptide separation gradient was a 120‐min linear gradient. The IVK raw data files were processed with MaxQuant version 1.6.0.16 (Cox & Mann, 2008). MS spectra were searched against the human component of the UniProtKB database (release 2017_12 with 20192 entries) using the Andromeda search engine (Cox *et al*, 2011). Carbamidomethylation (+ 57.021 Da) of cysteine residues was used as static modification. Heavy phosphorylation of serine/threonine/tyrosine (+ 85.966 Da) and oxidation (+ 15.994 Da) of methionine were used as dynamic modifications. Precursor mass tolerance and fragment mass tolerance were set to < 20 ppm and 0.1 Da, respectively. A maximum of two missed cleavages was allowed. The results were filtered to a maximum false discovery rate (FDR) of 0.05. Processed data were analyzed manually and filtered based on localization probability with a cutoff at 0.75. Any phosphotyrosine sites that were identified in control experiments without added kinase were also discarded.

### Data filtering steps

Significance Analysis of INTeractome (SAINT) express version 3.6.0 (Choi *et al*, 2011) and Contaminant Repository for Affinity Purification (CRAPome, http://www.crapome.org) (Mellacheruvu *et al*, 2013) were used as statistical tools for identification of specific high‐confidence interactions from AP‐MS and BioID data. 70 control runs with MAC‐Tagged GFP were used as controls for SAINT analysis. Identifications with a SAINT‐assigned Bayesian FDR ≥ 0.05 were dropped, as well as any proteins that were detected in ≥ 20% of CRAPome experiments, unless the spectral count fold change was over 3 when compared to CRAPome average. The remaining HCIs were then used for further analysis. For the IVK method, any phosphosites with < 75% localization probability as assigned by MaxQuant were discarded, as were sites that were detected in any control sample.

### Databases

Known interactors were mapped from BioGRID (only experimentally detected interactions) (Oughtred *et al*, 2021), Bioplex (interactions with probability over 0.95) (Huttlin *et al*, 2021), human cellmap (Go *et al*, 2021), IntAct (only experimentally validated physical interactions) (Orchard *et al*, 2014), PINA2 (Cowley *et al*, 2012), and STRING (only with a STRING score > 0.9) databases (Szklarczyk *et al*, 2019). Number of citations per RTK were taken from gene2pubmed.gz file provided by NCBI at ftp://ftp.ncbi.nlm.nih.gov/gene/DATA/gene2pubmed.gz (May 2020). Domain annotations were mapped from PFam (El‐Gebali *et al*, 2019). Reactome annotations from Uniprot to the lowest pathway level mapping file available at reactome (Fabregat *et al*, 2018). Gene ontology and CORUM (Giurgiu *et al*, 2019) annotations were taken from UniProt. GOCC annotations for CORUM complexes were taken from the CORUM database (Giurgiu *et al*, 2019). Known phosphosites, and kinases if available, were mapped from human protein reference database (Peri *et al*, 2003), PhosphoSitePlus (Hornbeck *et al*, 2015), phospho.ELM. (Dinkel *et al*, 2011), and a dataset from Sugiyama *et al* (2019).

We checked expression status of our HCIs and our bait proteins against the Human Protein Atlas database version 20.1. (Uhlen *et al* 2017) using RNA HPA cell line gene data that details the expression levels per gene in 69 different cell lines and against the RNA consensus tissue gene data that summarizes expression per gene in 62 tissues (downloaded 8.7.2021). In both cases, “not expressed” was judged to be a missing value or < 1 normalized expression (NX) value.

### Bioinformatic analyses

Enrichment values were calculated with an in‐house python script using all identified proteins before any filtering steps were applied as the background set. Prey–prey cross‐correlation was calculated with in‐house python script using scipy (Virtanen *et al*, 2020), and prey–prey associations from the correlation matrix were filtered based on q‐value (< 0.01), calculated with scipy using FDR correction (Benjamini & Hochberg, 1995), and correlation value (> 0.7). Kinase domain sequence‐based clustering was done with clustal Omega (Madeira *et al*, 2019) using default settings and kinase domain sequences extracted from UniProt. Clustering of phosphotyrosine sites and AP‐MS and BioID data was performed in R with the seqinr and dendextend libraries. Clustering for heatmaps was performed in python using seaborn. Network figures were drawn with cytoscape 3.7 (Kohl *et al*, 2011). Fold change values for KD RTKs versus WT were calculated with an in‐house python script using the WT kinase interactome as the background set. Random networks were generated by replacing HCIs in the RTK interactome with random proteins drawn from the background set of all identified proteins before any filtering steps were applied.

### Immunofluorescence confocal microscopy

The specific RTK expressing HEK293 cells were grown on glass coverslips. After 24 h, cells were washed with PBS prior to fixation in 4% (wt/vol) paraformaldehyde (PFA) in PBS for 15 min at room temperature. Cells were then washed with PBS and permeabilized by 4 min of incubation in 0.1% (wt/vol) Triton X‐100 in PBS. Bait proteins were detected with the anti‐HA antibody (Thermo Fisher Scientific, Cat. No. 26183, dilution 1:1,000 dilution), followed by Alexa Fluor488‐conjugated secondary antibody (Thermo Fisher Scientific, A‐11001, 1:1,000 dilution). The nucleus was stained with DAPI (Sigma, Cat. No. D9542). Finally, coverslips were dried before mounting in Mowiol 4‐88(Sigma, Cat. No. 81381). Prepared slides were analyzed using a confocal microscope (Leica TCS SP8 STED, Leica) with HC PL APO 93×/1.30 motCORR glycerol objective. Images were processed using ImageJ software (MacBiophotonics).

### Signal pathway analysis and luciferase assay

Cignal 45‐Pathway Reporter Array (Qiagen, Cat. No. 336841) was used to monitor the corresponding signaling pathway activity. Briefly, 30 μl Opti‐MEM containing dilute Attractene Transfection Reagent (Qiagen, Cat. No. 301005) was added to each well of the Cignal Finder Array plate coated with pre‐formulated, transfection‐ready reporter construct and test gene of interest construct, incubating at room temperature for 20 min. Subsequently, was added to DNA construct mixtures, 100 μl of HEK293 cell suspension containing 4 × 10^4^ cells in DMEM medium with 10% FBS was added to each well. After 24 h of transfection, the medium was changed to complete growth medium and further incubated for 24 h, followed by Dual‐Luciferase Reporter Assay System that was performed according to the manufacturer’s protocol (Promega, Cat. No. E1960).

### Co‐immunoprecipitation

To validate the RTK–RTK interactions, HEK293 cells were co‐transfected using Fugene 6 transfection reagent (Promega) with MAC‐tag (600 ng) and V5‐tag (600 ng) bait and prey constructs on 6‐well cell culture plates with 500,000 cells per well. For validation of other RTK–protein interactions (Dataset EV1C), Strep‐HA‐tagged prey (500 ng) and V5‐tagged RTK (500 ng) constructs were transfected. 24 h (RTK–RTK) or 48 h (RTK–protein) post‐transfection cells were rinsed with ice‐cold 1X PBS and lysed with 1 ml HENN lysis buffer per well (50 mM HEPES pH 8.0 + 5 mM EDTA + 150 mM NaCl + 50 mM NaF + 0.5% IGEPAL + 1 mM DTT + 1 mM PMSF + 1.5 mM Na_3_VO_4_ + 1X Protease inhibitor cocktail (Sigma‐Aldrich)). Cell lysates were vortexed briefly and centrifuged (16,000 *g*, 20 min, 4°C) to remove cellular debris. 20 µl of Strep‐Tactin^®^ Sepharose^®^ resin (IBA Lifesciences GmbH) was washed in a microcentrifuge tube twice with 200 µl HENN lysis buffer (4,000 *g*, 1 min, 4°C). The clear lysate was collected and added to the washed Strep‐Tactin^®^ Sepharose^®^ resin and incubated on a rotating wheel (60 min, 4°C). After incubation, the samples were centrifuged (4,000 *g*, 1 min, 4°C), and the supernatant was discarded. The pellet was washed three times with 1 ml HENN lysis buffer (4,000 *g*, 30 s, 4°C). After the last wash, 60 µl of 2X Laemmli sample buffer was added directly to the beads and boiled at 95°C for 5 min. Samples were later used for immunodetection using western blot (RTK–RTK interactions) or dot blot (RTK–prey interactions). For western blotting, immunoprecipitated proteins were detected with monoclonal mouse anti‐V5 (Invitrogen) or mouse anti‐HA.11 (BioLegend) primary antibodies and polyclonal goat anti‐mouse HRP‐conjugated (GE Healthcare) secondary antibody. Signals were visualized by chemiluminescence using Amersham™ ECL™ Prime (Cytiva) for 5 min prior to imaging using iBright Imaging Systems (Thermo Fisher Scientific).

### Dot blot

The Bio‐Dot^®^ Microfiltration System (Bio‐Rad, 1703938) was assembled according to the manufacturer’s instructions. Ten microliters of the Co‐IP sample were spotted onto the nitrocellulose membrane in the center of the well and drained under vacuum pressure. The membrane were then blocked with 5% fat‐free milk in TBS‐T (0.05% Tween‐20 in TBS) for 60 min at RT and followed by incubation with primary antibody in TBS‐T (mouse anti‐V5 with a 1:5,000 dilution) overnight at 4°C. The membrane was washed three times with TBS‐T and incubated with secondary antibody conjugated with HRP (goat anti‐mouse IgG conjugated with horseradish peroxidase with a 1:2,000 dilution) for 60 min at RT. Amersham™ ECL™ Prime (Cytiva) solution was added to the membrane for imaging the blot using iBright Imaging Systems (Thermo Fisher). The same membrane was then stripped by incubating with Restore Plus Stripping buffer (Thermo Fisher) for 15 min and was re‐blocked with 5% fat‐free milk in TBS‐T for 60 min at RT. The membrane was then incubated with the other primary antibody in TBS‐T (Rabbit anti‐HA with a 1:2,000 dilution) overnight at 4°C for different detections.

## Author contributions

**Kari Salokas:** Conceptualization; Resources; Data curation; Software; Formal analysis; Validation; Investigation; Visualization; Methodology; Writing – original draft; Writing – review & editing. **Xiaonan Liu:** Validation; Investigation; Methodology; Writing – review & editing. **Tiina Ohman:** Investigation; Methodology; Writing – original draft. **Iftekhar Chowdhury:** Validation; Investigation. **Lisa Gawriyski:** Software. **Salla Keskitalo:** Validation; Investigation; Writing – review & editing. **Markku Varjosalo:** Conceptualization; Resources; Data curation; Formal analysis; Supervision; Funding acquisition; Validation; Investigation; Visualization; Methodology; Writing – original draft; Project administration; Writing – review & editing.

In addition to the CRediT author contributions listed above, the contributions in detail are:

KS and MV designed the study. KS generated cell lines and performed AP‐MS and BioID analyses. KS analyzed the AP‐MS, BioID, and ultraID data. TÖ performed the IVK experiments. KS and TÖ analyzed the IVK data. XL performed the luciferase analysis, ultraID experiment, and IF imaging. IC performed co‐IP experiments. LG analyzed expression data from the protein atlas. SK performed blotting experiments. KS and MV prepared the figures. KS, TÖ, and MV wrote the manuscript.

## Disclosure and competing interests statement

The authors declare that they have no conflict of interest.

## Supporting information



Appendix

Expanded View Figures PDF

Dataset EV1

Dataset EV2

Dataset EV3

Dataset EV4

Dataset EV5

Dataset EV6

Dataset EV7

Dataset EV8

Source Data for Expanded View

## Data Availability

Collected mass spectrometry data are available at MassIVE with dataset ID MSV000087816 (https://massive.ucsd.edu/ProteoSAFe/dataset.jsp?task=b45c797348cc484baff3e8100e4373e8).
