# Supplementary material for: Physical and functional interactome atlas of human receptor tyrosine kinases
Source: EMBO Rep. 2022 Apr 5;23(6):e54041. doi: 10.15252/embr.202154041 (PMC9171411; doi:10.15252/embr.202154041)
Supplement: Supplementary file 1 — Appendix [file EMBR-23-e54041-s007.pdf]

Table of contents:

Appendix figure S1 Enriched Reactome pathways and cross-correlation network functions - p. 2 - 3

Appendix figure S2 Analysis of EphA5-A8 interactome generated in this study – p. 4 - 5

Appendix figure S3 Overview of in-vitro kinase assay workflow and data – p. 6 - 7

Appendix figure S1 Enriched Reactome pathways and cross-correlation network functions

A. Heatmap of RTK HCIs in enriched (q<0.05, calculated with Fisher-exact test and Benjamini-Hochberg multiple-testing correction) GO biological process terms (top) compared to number of proteins from the combined database of known interactions (bottom). Values shown are log2 counts of proteins with the corresponding GOBP annotation. Clustering was done based on RTK HCIs, and order copied for the known interactor heatmap. Each row represents one RTK, and they are in the same order in both top and bottom heatmaps.

B. Average number of known protein-protein interaction pairs in random networks of the same topology, as the cross-correlation network (top) and protein pairs in the same lowest level Reactome networks (bottom). Value for the cross-correlation network is indicated by the line on the bottom end.

C. Enriched (q<0.05, calculated with Fisher-exact test and Benjamini-Hochberg multiple-testing correction) GOBP terms in the four biggest clusters identified in the cross-correlation network.

D. Identified CORUM protein complexes in the cross-correlation network. Identified protein subunits from complexes with at least two identified components are shown. Complexes with <60% of subunits identified were also filtered out. With proteins shared by multiple complexes, color coding is used to differentiate what omplex each protein participate in.

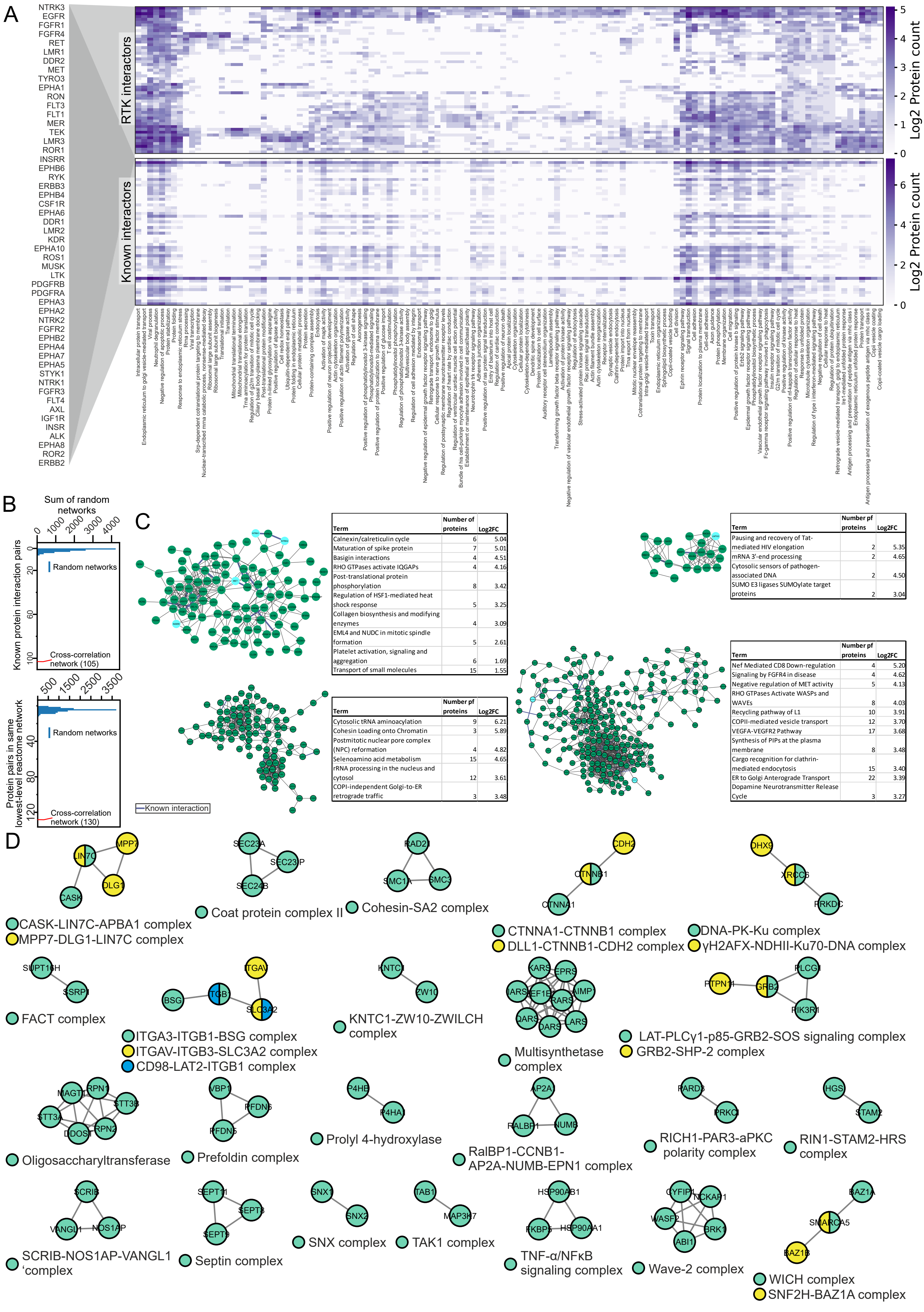

Term	Number of proteins	Log2FC
Calnexin/calreticulin cycle	6	5.04
Maturation of spike protein	7	5.01
Basigin interactions	4	4.51
RHO GTPases activate IQGAPs	4	4.16
Post-translational protein phosphorylation	8	3.42
Regulation of HSF1-mediated heat shock response	5	3.25
Collagen biosynthesis and modifying enzymes	4	3.09
EML4 and NUDC in mitotic spindle formation	5	2.61
Platelet activation, signaling and aggregation	6	1.69
Transport of small molecules	15	1.55

Term	Number pf proteins	Log2FC
Pausing and recovery of Tat-mediated HIV elongation	2	5.35
mRNA 3'-end processing	2	4.65
Cytosolic sensors of pathogen-associated DNA	2	4.50
SUMO E3 ligases SUMOylate target proteins	2	3.04

Term	Number pf proteins	Log2FC
Cytosolic tRNA aminoacylation	9	6.21
Cohesin Loading onto Chromatin	3	5.89
Postmitotic nuclear pore complex (NPC) reformation	4	4.82
Selenoamino acid metabolism	15	4.65
rRNA processing in the nucleus and cytosol	12	3.61
COPI-independent Golgi-to-ER retrograde traffic	3	3.48

Term	Number pf proteins	Log2FC
Nef Mediated CD8 Down-regulation	4	5.20
Signaling by FGFR4 in disease	4	4.62
Negative regulation of MET activity	5	4.13
RHO GTPases Activate WASPs and WAVEs	8	4.03
Recycling pathway of L1	10	3.91
COPII-mediated vesicle transport	12	3.70
VEGFA-VEGFR2 Pathway	17	3.68
Synthesis of PIPs at the plasma membrane	8	3.48
Cargo recognition for clathrin-mediated endocytosis	15	3.40
ER to Golgi Anterograde Transport	22	3.39
Dopamine Neurotransmitter Release Cycle	3	3.27

Appendix figure S2 Analysis of EphA5-A8 interactome generated in this study

A. Shared HCIs between EphA5, A6, A7, and A8. Green lines depict interactions seen in BioID data, blue for AP-MS, and burgundy for both. Grey dashed lines are known HCI-HCI interactions from IntAct.

B. Enriched ($q<0.05$, calculated with Fisher-exact test and Benjamini-Hochberg multiple-testing correction) Reactome pathways and Corum complexes in the EphA5-A8 data. Values are log2 fold change for Reactome pathways and number of identified complex subunits for CORUM data.

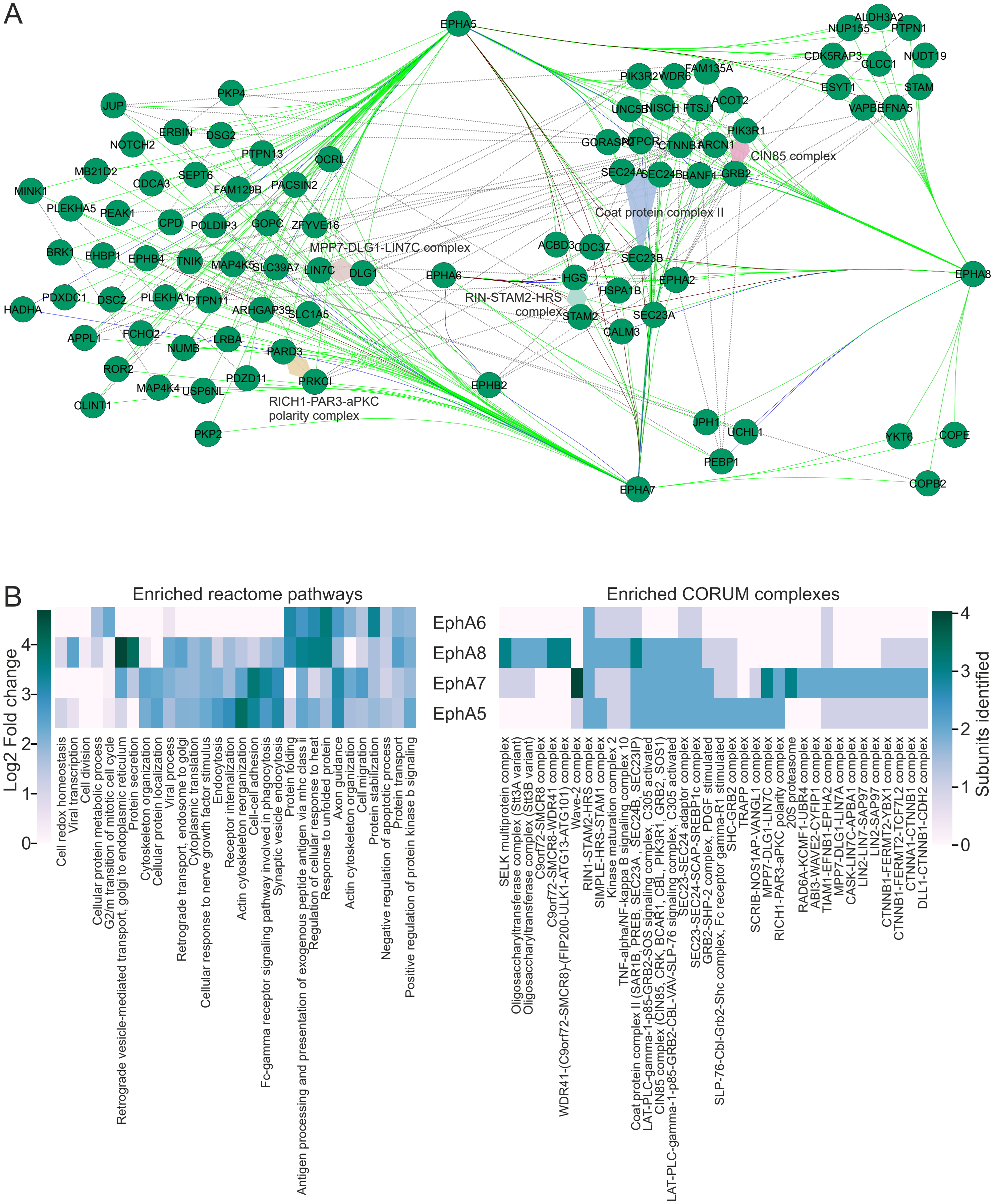

A
EPHA5
EPHA6
EPHA7
EPHA8
EPHA2
EPHB2
EPHB4
MPP7-DLG1-LIN7C complex
RICH1-PAR3-aPKC
polarity complex
CIN85 complex
Coat protein complex II
RIN-STAM2-HRS
complex
JUP
PKP4
NOTCH2
ERBIN
DSG2
PTPN13
OCRL
MB21D2
SEPT6
CDCA3
FAM129B
PACSIN2
MINK1
PLEKHA5
PEAK1
CPD
POLDIP3
GOPC
ZFYVE16
BRK1
EHBP1
TNIK
MAP4K5
SLC39A7
LIN7C
DLG1
HADHA
PDXDC1
DSC2
PLEKHA1
PTPN11
ARHGAP39
SLC1A5
APPL1
FCHO2
NUMB
LRBA
PARD3
ROR2
MAP4K4
USP6NL
PDZD11
PRKCI
CLINT1
PKP2
PIK3R2
WDR6
FAM135A
UNC5B
NISCH
FTSJ1
ACOT2
GORASP2
PTPCR
CTNNB1
ARCN1
PIK3R1
SEC24A
SEC24B
BANF1
GRB2
ACBD3
CDC37
SEC23B
HGS
HSPA1B
STAM2
SEC23A
CALM3
NUP155
ALDH3A2
PTPN1
CDK5RAP3
CLCC1
NUDT19
ESYT1
STAM
VAPB
EFNA5
JPH1
UCHL1
PEBP1
YKT6
COPE
COPB2
B
Enriched reactome pathways
Enriched CORUM complexes
Log2 Fold change
Subunits identified
EphA6
EphA8
EphA7
EphA5
Cell redox homeostasis
Viral transcription
Cell division
Cellular protein metabolic process
G2/m transition of mitotic cell cycle
Retrograde vesicle-mediated transport, golgi to endoplasmic reticulum
Protein secretion
Cytoskeleton organization
Cellular protein localization
Viral process
Retrograde transport, endosome to golgi
Cytoplasmic translation
Cellular response to nerve growth factor stimulus
Endocytosis
Receptor internalization
Actin cytoskeleton reorganization
Cell-cell adhesion
Fc-gamma receptor signaling pathway involved in phagocytosis
Synaptic vesicle endocytosis
Protein folding
Antigen processing and presentation of exogenous peptide antigen via mhc class ii
Regulation of cellular response to heat
Response to unfolded protein
Axon guidance
Actin cytoskeleton organization
Cell migration
Protein stabilization
Negative regulation of apoptotic process
Protein transport
Positive regulation of protein kinase b signaling
SELK multiprotein complex
Oligosaccharyltransferase complex (Stt3A variant)
Oligosaccharyltransferase complex (Stt3B variant)
C9orf72-SMCR8 complex
C9orf72-SMCR8-WDR41 complex
WDR41-(C9orf72-SMCR8)-(FIP200-ULK1-ATG13-ATG101) complex
Wave-2 complex
RIN1-STAM2-HRS complex
SIMPLE-HRS-STAM1 complex
Kinase maturation complex 2
TNF-alpha/NF-kappa B signaling complex 10
Coat protein complex II (SAR1B, PREB, SEC23A , SEC24B, SEC23IP)
LAT-PLC-gamma-1-p85-GRB2-SOS signaling complex, C305 activated
CIN85 complex (CIN85, CRK, BCAR1, CBL, PIK3R1, GRB2, SOS1)
LAT-PLC-gamma-1-p85-GRB2-CBL-VAV-SLP-76 signaling complex, C305 activated
SEC23-SEC24 adaptor complex
SEC23-SEC24-SCAP-SREBP1c complex
GRB2-SHP-2 complex, PDGF stimulated
SLP-76-Cbl-Grb2-Shc complex, Fc receptor gamma-R1 stimulated
SHC-GRB2 complex
TRAPP complex
SCRIB-NOS1AP-VANGL1 complex
MPP7-DLG1-LIN7C complex
RICH1-PAR3-aPKC polarity complex
20S proteasome
RAD6A-KCMF1-UBR4 complex
ABI3-WAVE2-CYFIP1 complex
TIAM1-EFNB1-EPHA2 complex
MPP7-DLG1-LIN7A complex
CASK-LIN7C-APBA1 complex
LIN2-LIN7-SAP97 complex
LIN2-SAP97 complex
CTNNB1-FERMT2-YBX1 complex
CTNNB1-FERMT2-TCF7L2 complex
CTNNA1-CTNNB1 complex
DLL1-CDH2 complex

Appendix figure S3 Overview of in-vitro kinase assay workflow and data

A. In-vitro kinase assay (IVK) Workflow used in this study. After silencing endogenous cellular kinases from the cell lysate with FSBA, the RTK of interest was added with heavy ATP, after which tyrosine-phosphopeptides with $^{18}O_4$-ATP were enriched and identified with a mass spectrometer.

B. Phosphotyrosine site counts divided into groups based on how many baits they were identified with. Bar for sites identified with only one bait was split apart from the rest to show more detail. Inset on the right side shows the total number of phosphorylated tyrosine sites identified in the control experiments with no added recombinant kinase after filters were applied.

C. Comparison of AP-MS and BioID based RTK dendrograms.

D. Number of substrate proteins detected in IVK in each Reactome signaling pathway group. Numbers reflect the number of unique substrate proteins annotated with a reactome pathway of each general pathway group.

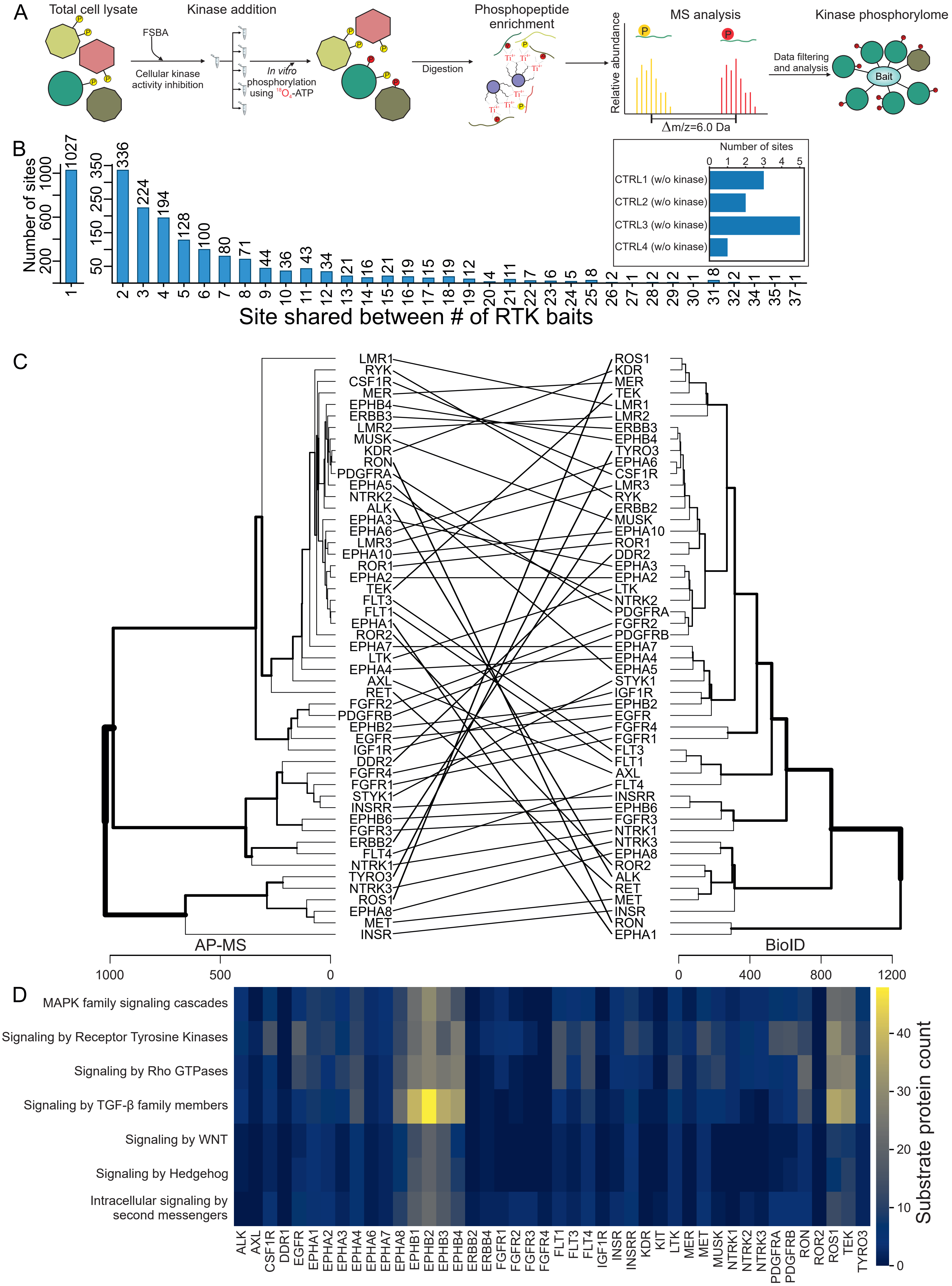
A
Total cell lysate
Kinase addition
FSBA
Cellular kinase activity inhibition
In vitro phosphorylation using $^{18}O_4$-ATP
Digestion
Phosphopeptide enrichment
MS analysis
Relative abundance
Δm/z=6.0 Da
Data filtering and analysis
Kinase phosphorylome
Bait
B
Number of sites
Site shared between # of RTK baits
1027
336
224
194
128
100
80
71
44
36
43
34
21
16
21
19
15
19
12
4
11
7
6
5
8
2
1
2
2
1
8
2
1
1
1
Number of sites
CTRL1 (w/o kinase)
CTRL2 (w/o kinase)
CTRL3 (w/o kinase)
CTRL4 (w/o kinase)
C
AP-MS
BioID
LMR1
RYK
CSF1R
MER
EPHB4
ERBB3
LMR2
MUSK
KDR
RON
PDGFRA
EPHA5
NTRK2
ALK
EPHA3
EPHA6
LMR3
EPHA10
ROR1
EPHA2
TEK
FLT3
FLT1
EPHA1
ROR2
EPHA7
LTK
EPHA4
AXL
RET
FGFR2
PDGFRB
EPHB2
EGFR
IGF1R
DDR2
FGFR4
FGFR1
STYK1
INSRR
EPHB6
FGFR3
ERBB2
FLT4
NTRK1
TYRO3
NTRK3
ROS1
EPHA8
MET
INSR
D
MAPK family signaling cascades
Signaling by Receptor Tyrosine Kinases
Signaling by Rho GTPases
Signaling by TGF-β family members
Signaling by WNT
Signaling by Hedgehog
Intracellular signaling by second messengers
Substrate protein count
ALK
AXL
CSF1R
DDR1
EGFR
EPHA1
EPHA2
EPHA3
EPHA4
EPHA6
EPHA7
EPHA8
EPHB1
EPHB2
EPHB3
EPHB4
ERBB2
ERBB4
FGFR1
FGFR2
FGFR3
FGFR4
FLT1
FLT3
FLT4
IGF1R
INSR
INSRR
KDR
KIT
LTK
MER
MET
MUSK
NTRK1
NTRK2
NTRK3
PDGFRA
PDGFRB
RON
ROR2
ROS1
TEK
TYRO3
