# Supplementary material for: Physical and functional interactome atlas of human receptor tyrosine kinases
Source: EMBO Rep. 2022 Apr 5;23(6):e54041. doi: 10.15252/embr.202154041 (PMC9171411; doi:10.15252/embr.202154041)
Supplement: Supplementary file 2 — Expanded View Figures PDF [file EMBR-23-e54041-s004.pdf]

Expanded View Figures

Figure EV1. Detailed workflows used in this study and overall data assessment.

A Workflows used in this study for affinity purification coupled with mass spectrometry (AP-MS) and BioID approaches. AP-MS enables the capture of protein complexes and stoichiometries, whereas the complementary BioID method enables capture of direct, transient/proximal interactions.
B Network topologies of RTK subfamilies. Blue nodes indicate the bait protein used in the experiment and green nodes the detected HCI proteins. Interactions detected in AP-MS only are marked in blue, BioID only in green, and interactions detected with both approaches are shown in burgundy.
C Proportion of HCIs shared between RTK subfamilies. Values are calculated based on the number of shared HCIs and the number of HCIs in each subfamily.
D Anti-phosphotyrosine blot for 8 RTKs, showing bait RTK phosphorylation.
E Co-IP validation of RTK–RTK interactions detected via AP-MS method in the study. Spectral count values for the interactions are shown underneath.

Source data are available online for this figure.

© 2022 The Authors

© 2022 The Authors

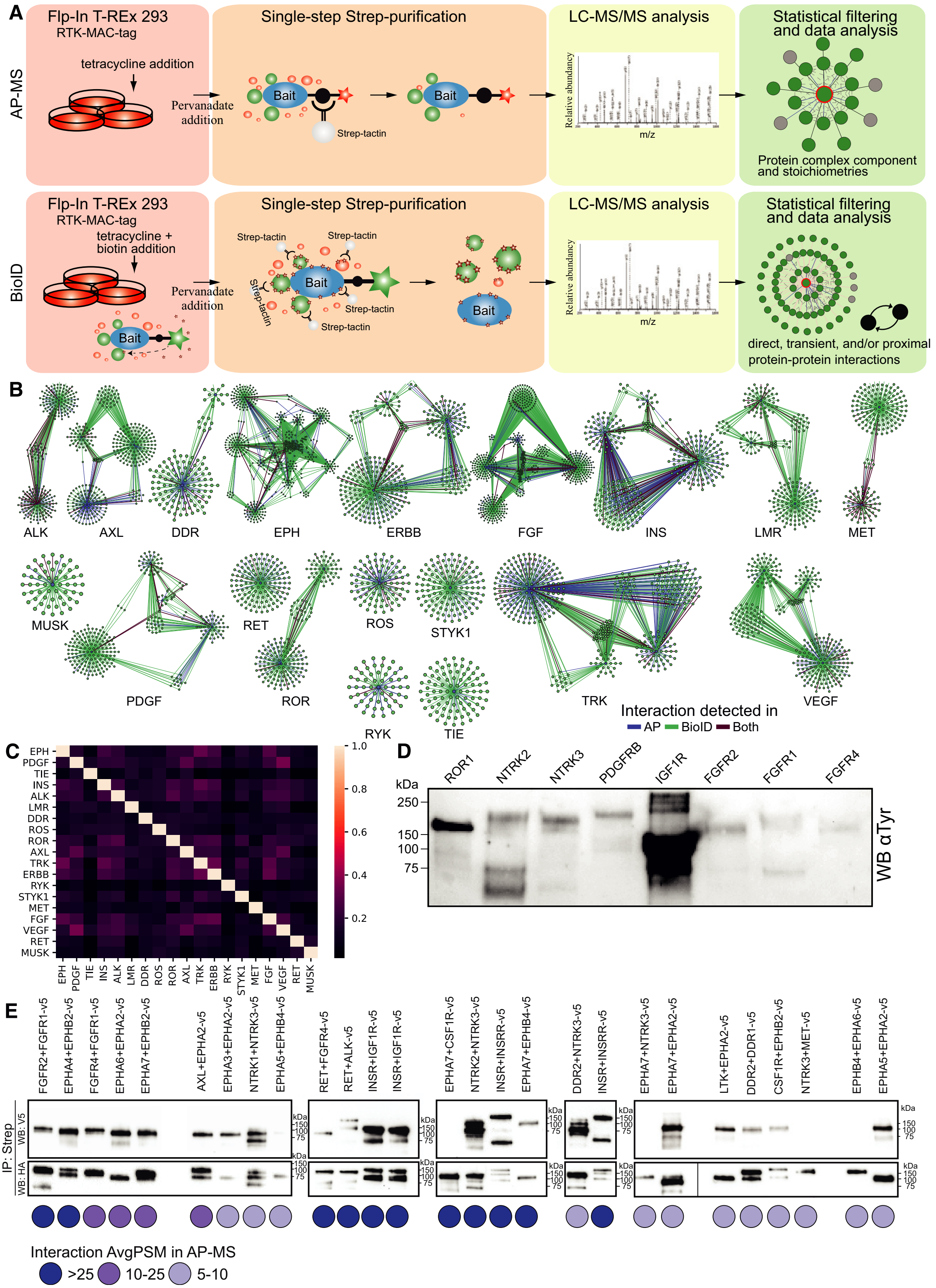

Figure EV1.

 © 2022 The Authors

Figure EV2. RTK localization and AP-MS validation via CO-IP.

A Immunofluorescence microscopy images of each bait RTK. Images are divided based on the RTK subfamily. Green: Anti-HA, Blue: DAPI. Scale bar 10 μm.

B Verification of several AP-MS high-confidence interactions with Co-IP and dot blot. 83 interactions were tested, of which 69 were detected via Co-IP. The prey proteins were tagged with Strep-HA, and bait proteins with V5 and coexpressed in HEK293 cells. For negative controls, Strep-HA-tagged GFP and V5-tagged RTKs were used. The Strep-HA tagged proteins were immunoprecipitated with Strep-Tactin sepharose. The immunoprecipitated protein complexes were then dot blotted with anti-V5 and anti-HA antibodies.

© 2022 The Authors

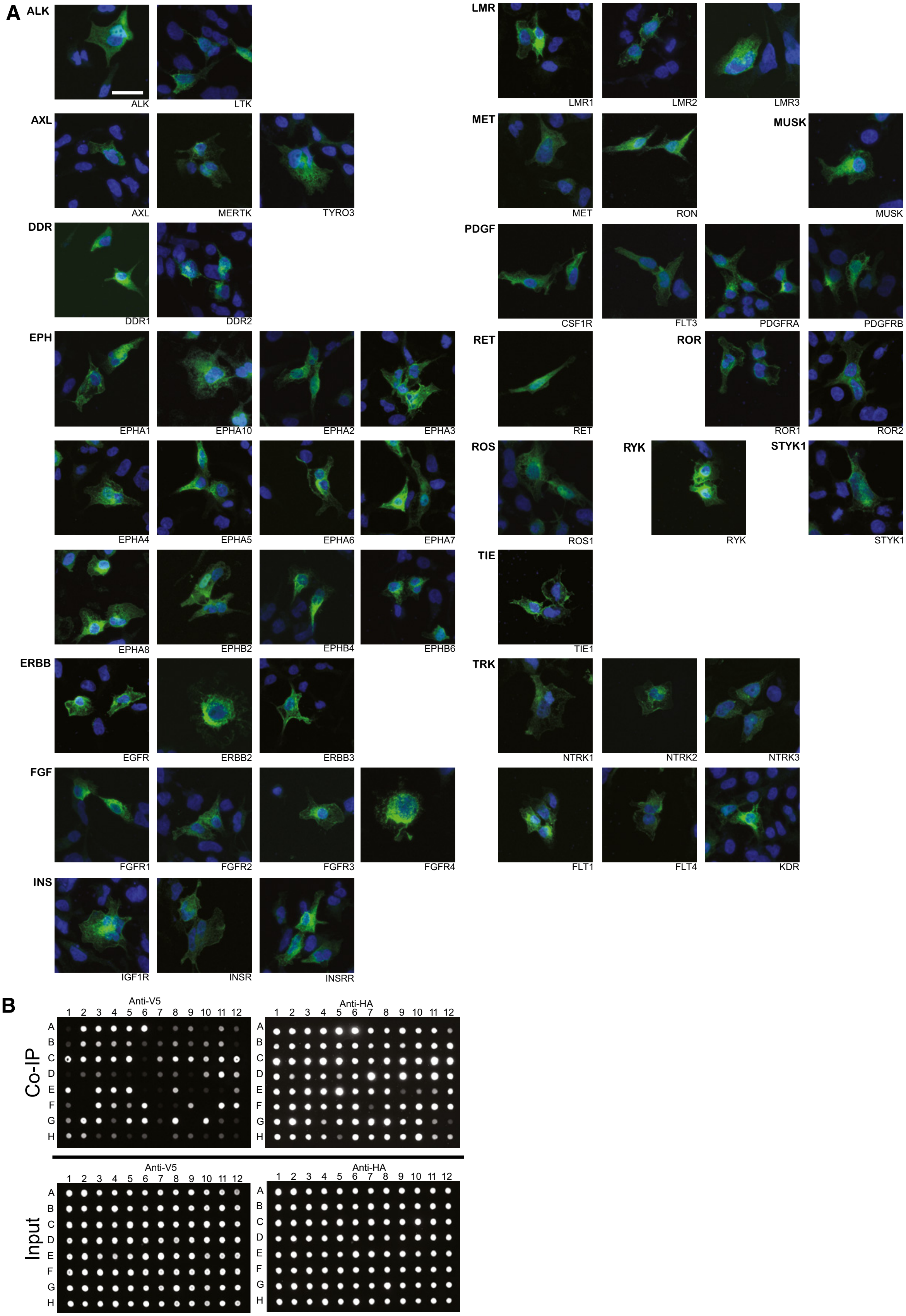

Figure EV2.

 © 2022 The Authors

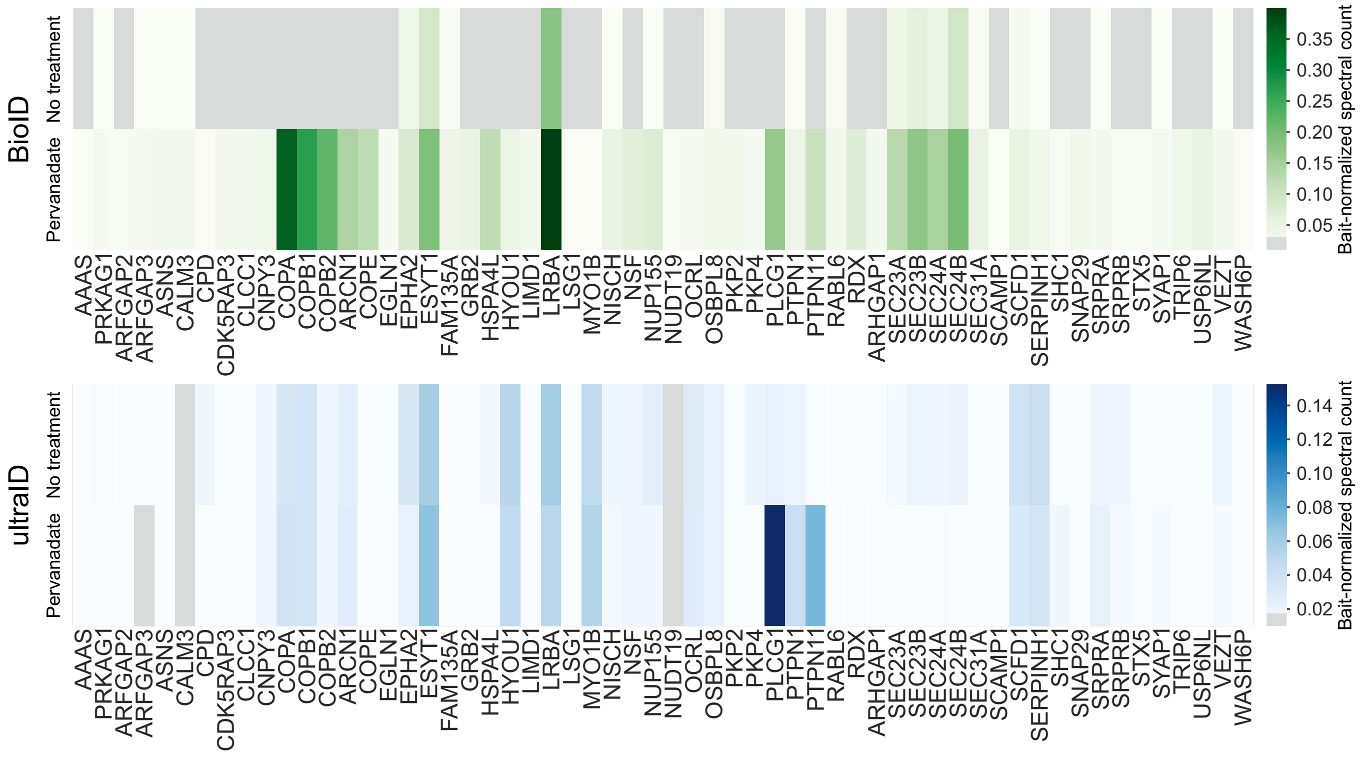

Figure EV3. 15-min pervanadate treatment compared to no treatment with NTRK3 BioID and ultraID experiments.

HCI proteins of pervanadate-treated NTRK3 detected by proximity labeling approaches (BioID or UltraID) are shown to highlight what interactions are treatment-dependent. With ultraID (preprint: Zhao *et al*, 2021), 10-min biotinylation time was used for identifying interactions at the time of activation. For visualization purposes, only proteins with significant changes (bait-normalized average spectral count difference > 2 fold, or exclusively identified in pervanadate treatment) were selected to be presented here.

© 2022 The Authors

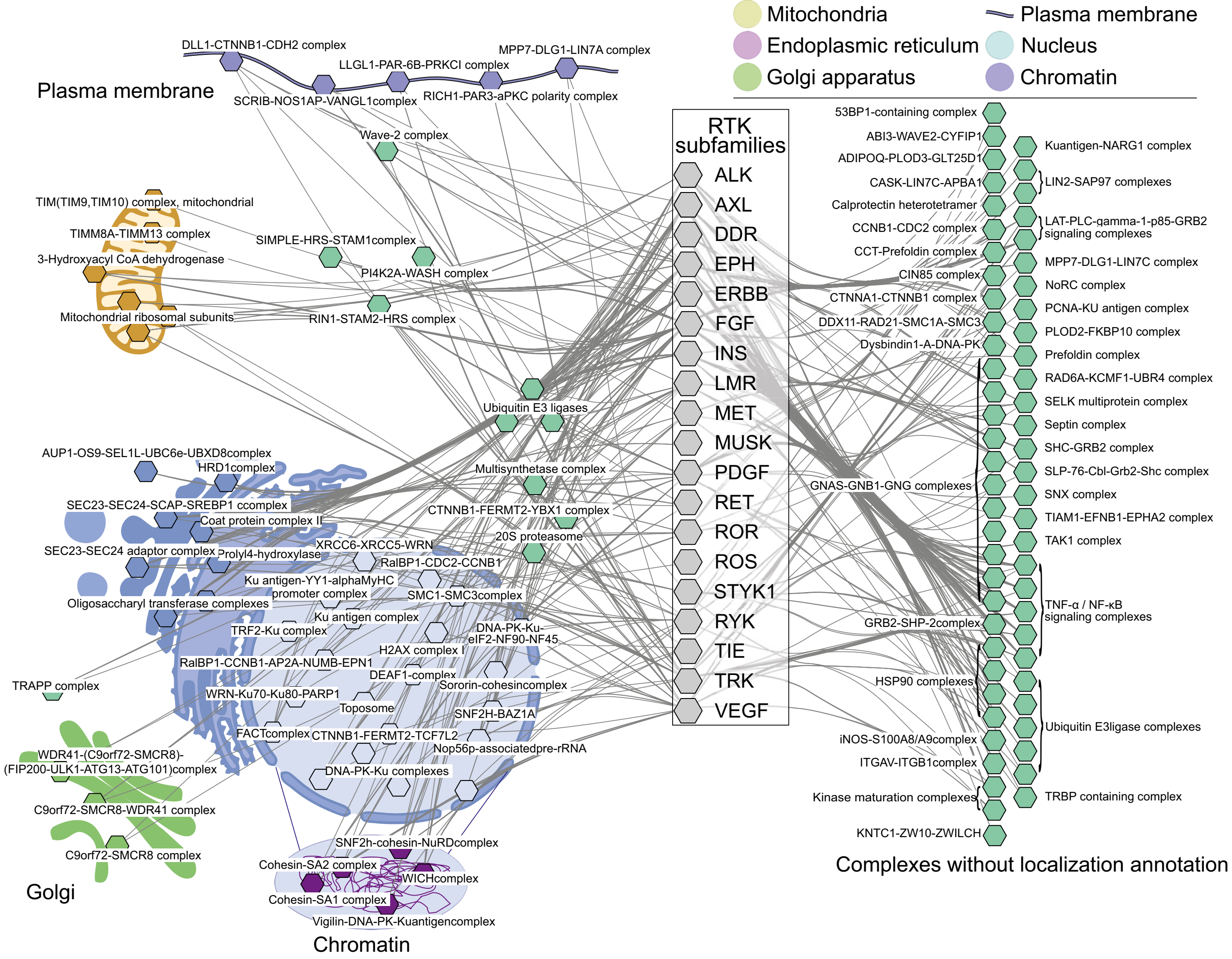

Figure EV4. Enriched complexes in the RTK interactome data.

Significantly ($q < 0.05$, calculated with Fisher exact test followed by Benjamini–Hochberg multiple-testing correction) enriched CORUM complexes in the interactomes of the RTK subfamilies. The cellular localization was assigned to each complex with available GO cellular component in CORUM. Connections from subfamilies to complexes denote significant enrichment of the complex with one or more members of the subfamily. On the right side, complexes without localization information are grouped based on their protein composition.

Figure EV5. Analysis of EGFR interactome generated in this study.

- A EGFR HCIs identified in the study. Previously known interactors are denoted in brown, and novel in green.
- B Most common significantly ($q < 0.05$, calculated with Fisher exact test and Benjamini–Hochberg multiple-testing correction) enriched GO Biological process annotation terms in the EGFR HCIs. The top chart shows unique protein counts, while the lower chart depicts log2 fold change over expected value calculated from the background set.
- C Known interactions between previously known HCIs and novel HCIs.
- D Co-IP validation of a subgroup of EGFR (left) and NTRK3 (right) interactors.

Source data are available online for this figure.

 © 2022 The Authors

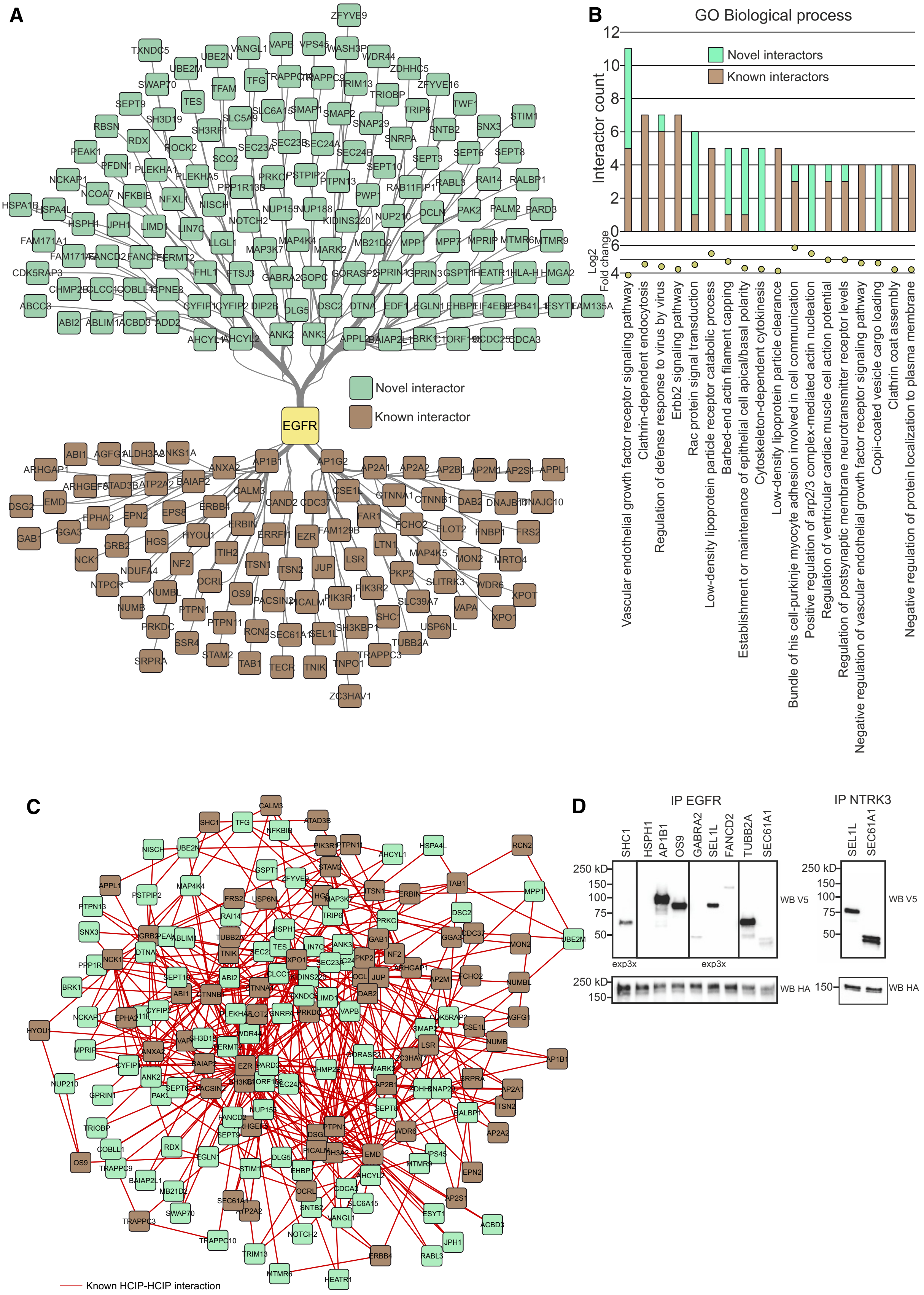
A
Novel interactor
Known interactor
EGFR
B
GO Biological process
Novel interactors
Known interactors
Interactor count
Log2 Fold change
Vascular endothelial growth factor receptor signaling pathway
Clathrin-dependent endocytosis
Regulation of defense response to virus by virus
Erbb2 signaling pathway
Rac protein signal transduction
Low-density lipoprotein particle receptor catabolic process
Barbed-end actin filament capping
Establishment or maintenance of epithelial cell apical/basal polarity
Cytoskeleton-dependent cytokinesis
Low-density lipoprotein particle clearance
Bundle of his cell-purkinje myocyte adhesion involved in cell communication
Positive regulation of arp2/3 complex-mediated actin nucleation
Regulation of ventricular cardiac muscle cell action potential
Regulation of postsynaptic membrane neurotransmitter receptor levels
Negative regulation of vascular endothelial growth factor receptor signaling pathway
Copii-coated vesicle cargo loading
Clathrin coat assembly
Negative regulation of protein localization to plasma membrane
C
Known HCIP-HCIP interaction
D
IP EGFR
IP NTRK3
SHC1
HSPH1
AP1B1
OS9
GABRA2
SEL1L
FANCD2
TUBB2A
SEC61A1
250 kD
150
100
75
50
WB V5
WB HA
exp3x

Figure EV5.
