# Supplementary figures and images for: Physical and functional interactome atlas of human receptor tyrosine kinases
Source: EMBO Rep. 2022 Apr 5;23(6):e54041. doi: 10.15252/embr.202154041 (PMC9171411; doi:10.15252/embr.202154041)

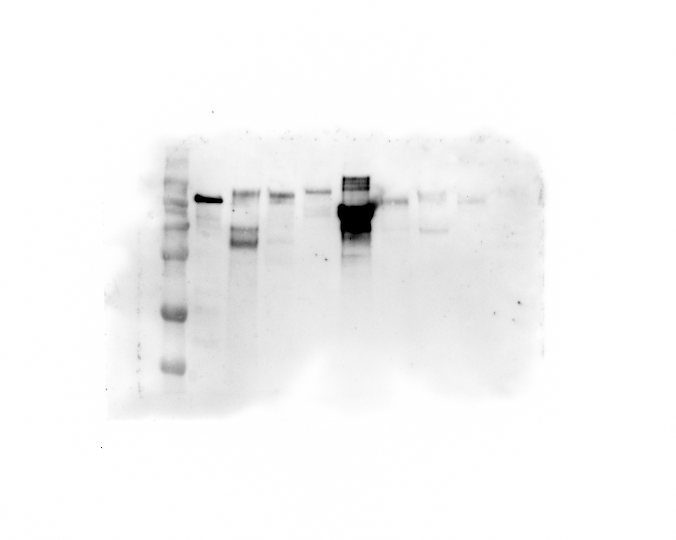

Supplement: Supplementary file 11 — Source Data for Expanded View [file EMBR-23-e54041-s006.zip › Source data for Expanded View Figure EV1/Source data for EV1D.tif]

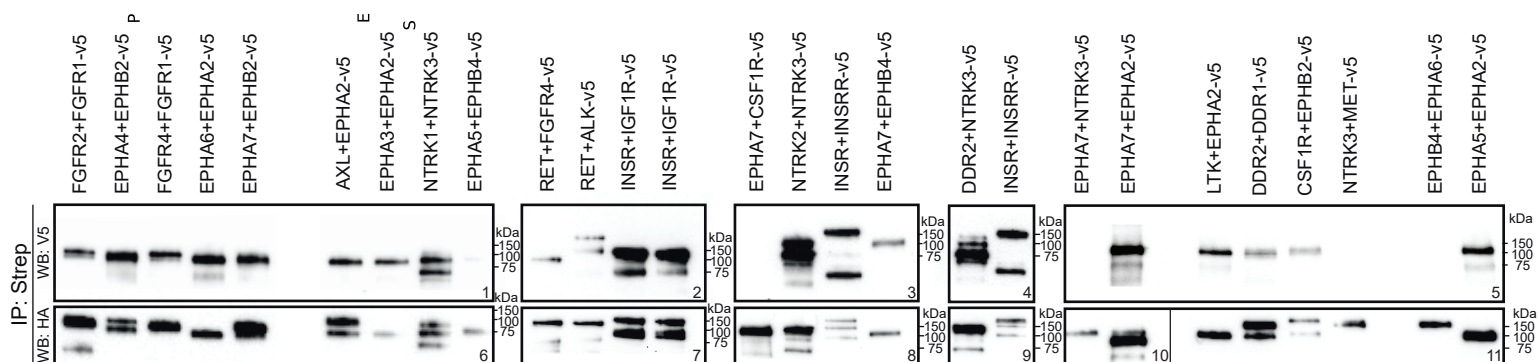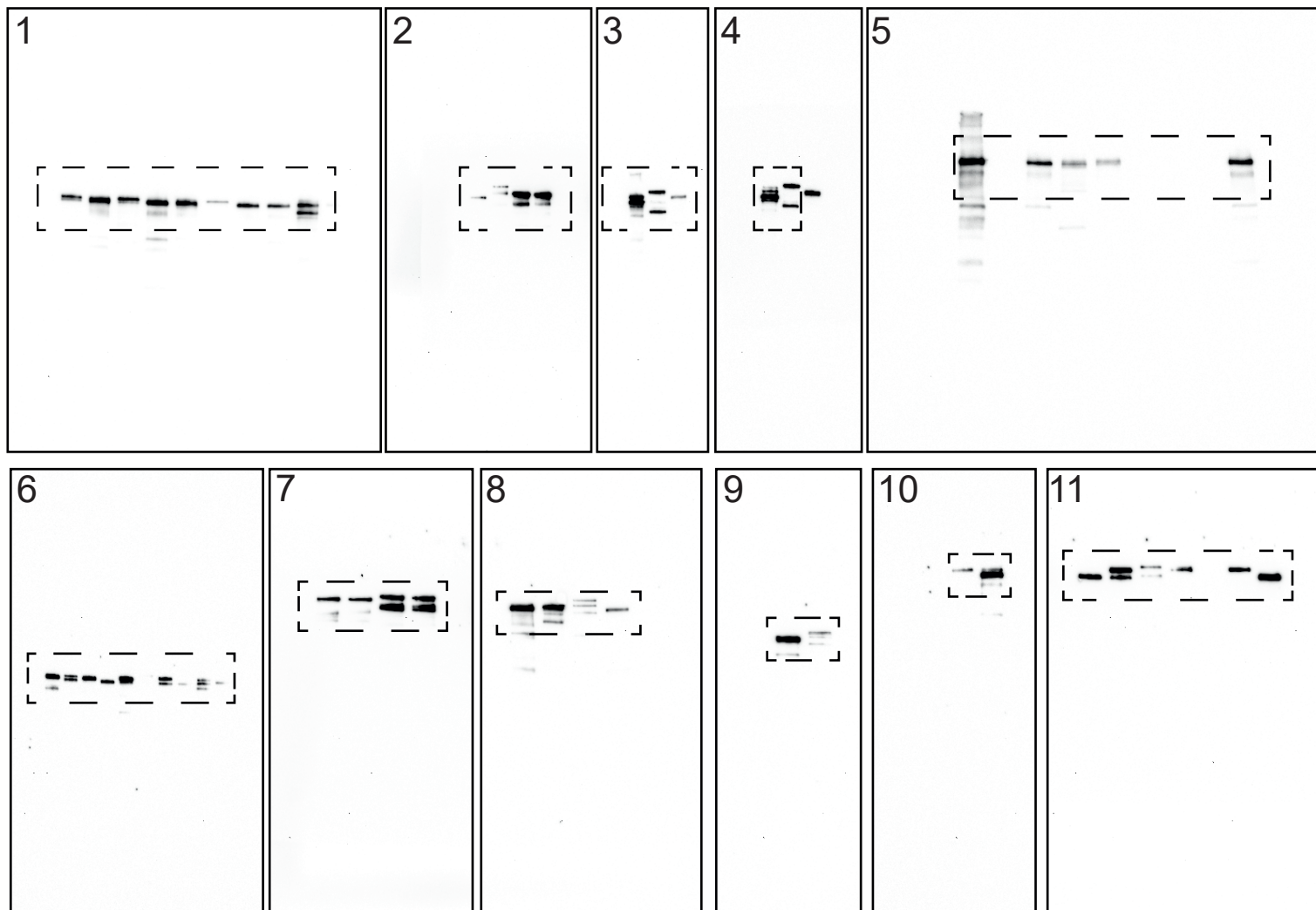

Supplement: Supplementary file 11 — Source Data for Expanded View [file EMBR-23-e54041-s006.zip › Source data for Expanded View Figure EV1/Source data for EV1E.pdf]

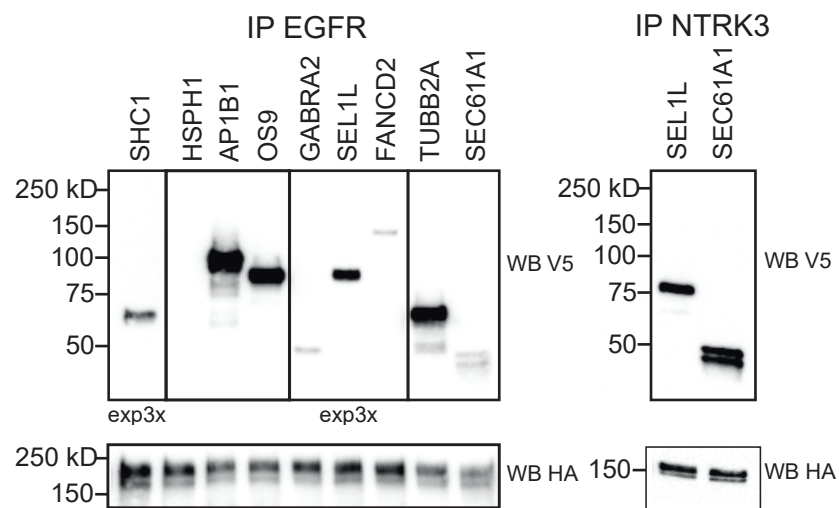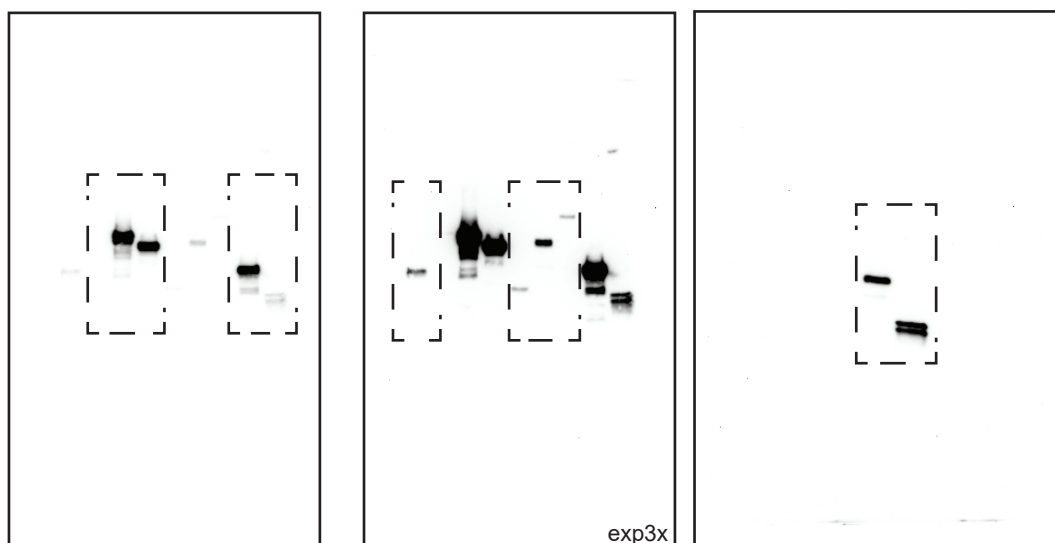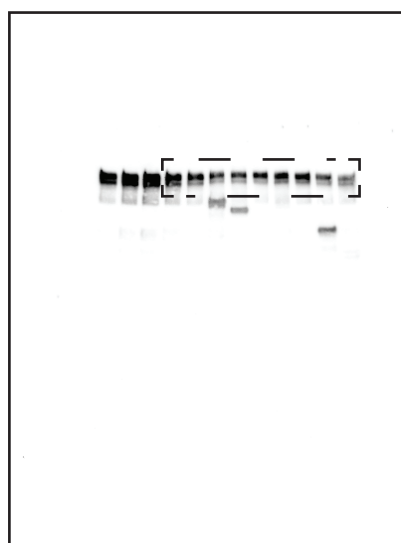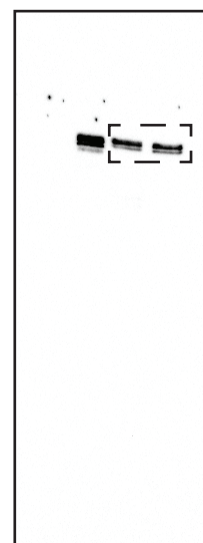

Supplement: Supplementary file 11 — Source Data for Expanded View [file EMBR-23-e54041-s006.zip › Source data for Expanded View Figure EV5/Source data for EV5D.pdf]
