# Supplementary material for: Physical and functional interactome atlas of human receptor tyrosine kinases
Source: EMBO Rep. 2022 Apr 5;23(6):e54041. doi: 10.15252/embr.202154041 (PMC9171411; doi:10.15252/embr.202154041)
Supplement: Supplementary file 12 [file EMBR-23-e54041-s011.pdf]

EMBO reports

# Physical and functional interactome atlas of human receptor tyrosine kinases

Kari Salokas, Xiaonan Liu, Tiina Öhman, Iftekhar Chowdhury, Lisa Gawriyski, Salla Keskitalo, and Markku Varjosalo
**DOI: 10.15252/embr.202154041**

*Corresponding author(s): Markku Varjosalo (markku.varjosalo@helsinki.fi)*



## Review Timeline:

| | |
|---|---|
| Submission Date: | 22nd Sep 21 |
| Editorial Decision: | 28th Oct 21 |
| Revision Received: | 28th Jan 22 |
| Editorial Decision: | 28th Feb 22 |
| Revision Received: | 3rd Mar 22 |
| Accepted: | 9th Mar 22 |

*Editor: Martina Rembold*


## Transaction Report:

(Note: With the exception of the correction of typographical or spelling errors that could be a source of ambiguity, letters and reports are not edited. Depending on transfer agreements, referee reports obtained elsewhere may or may not be included in this compilation. Referee reports are anonymous unless the Referee chooses to sign their reports.)

**1st Editorial Decision** **28th Oct 2021**

Dear Dr. Varjosalo

Thank you for the submission of your research manuscript to our journal. We have now received the full set of referee reports that is copied below.

As you will see, the referees acknowledge that the findings are potentially interesting. Referee 2, an expert in proteomics, has only minor concerns regarding data presentation and description. Referee 1, an expert in kinase-related research, raises concerns regarding the missing validation of at least some potential RTK interactors and their physiological relevance. I agree that the validation of some interactors would significantly strengthen the manuscript but this is not mandatory, since your manuscript will be published in our Resource section. Nevertheless, all concerns from referee 1 regarding potential artefacts induced by the ectopic expression of RTKs in HEK293-derivative cells and by the use of pervanadate need to be addressed and discussed.

Taken together, we would like to invite you to revise your manuscript with the understanding that the referee concerns (as detailed above and in their reports) must be fully addressed and their suggestions taken on board. Please address all referee concerns in a complete point-by-point response. Acceptance of the manuscript will depend on a positive outcome of a second round of review. It is EMBO reports policy to allow a single round of revision only and acceptance or rejection of the manuscript will therefore depend on the completeness of your responses included in the next, final version of the manuscript.

Revised manuscripts should be submitted within three months of a request for revision; they will otherwise be treated as new submissions. Please contact us if a 3-months time frame is not sufficient for the revisions so that we can discuss the revisions further.

***IMPORTANT NOTE: we perform an initial quality control of all revised manuscripts before re-review. Your manuscript will FAIL this control and the handling will be DELAYED if the following APPLIES:

1) A data availability section is missing.
2) Your manuscript contains error bars based on n=2. Please use scatter blots showing the individual datapoints in these cases. The use of statistical tests needs to be justified.

When submitting your revised manuscript, please carefully review the instructions that follow below. Failure to include requested items will delay the evaluation of your revision.***

When submitting your revised manuscript, we will require:

1) a .docx formatted version of the manuscript text (including legends for main figures, EV figures and tables). Please make sure that the changes are highlighted to be clearly visible.

2) individual production quality figure files as .eps, .tif, .jpg (one file per figure).
Please download our Figure Preparation Guidelines (figure preparation pdf) from our Author Guidelines pages https://www.embopress.org/page/journal/14693178/authorguide for more info on how to prepare your figures.

3) a .docx formatted letter INCLUDING the reviewers' reports and your detailed point-by-point responses to their comments. As part of the EMBO Press transparent editorial process, the point-by-point response is part of the Review Process File (RPF), which will be published alongside your paper.

4) a complete author checklist, which you can download from our author guidelines (). Please insert information in the checklist that is also reflected in the manuscript. The completed author checklist will also be part of the RPF.

5) Please note that all corresponding authors are required to supply an ORCID ID for their name upon submission of a revised manuscript (). Please find instructions on how to link your ORCID ID to your account in our manuscript tracking system in our Author guidelines
()

6) We replaced Supplementary Information with Expanded View (EV) Figures and Tables that are collapsible/expandable online. A maximum of 5 EV Figures can be typeset. EV Figures should be cited as 'Figure EV1, Figure EV2" etc... in the text and their respective legends should be included in the main text after the legends of regular figures.

- For the figures that you do NOT wish to display as Expanded View figures, they should be bundled together with their legends in a single PDF file called *Appendix*, which should start with a short Table of Content. Appendix figures should be referred to in

the main text as: "Appendix Figure S1, Appendix Figure S2" etc. See detailed instructions regarding expanded view here:

- Additional Tables/Datasets should be labeled and referred to as Table EV1, Dataset EV1, etc. Legends have to be provided in a separate tab in case of .xls files. Alternatively, the legend can be supplied as a separate text file (README) and zipped together with the Table/Dataset file.

- Please provide Supplementary table S1-S8 as Dataset EV1-8.

7) Please list the accession number and database for the mass-spectrometry data in a formal "Data Availability " section (placed after Materials & Method) that follows the model below (see also < https://www.embopress.org/page/journal/14693178/authorguide#dataavailability>). Please note that the Data Availability Section is restricted to new primary data that are part of this study.

# Data availability

The datasets (and computer code) produced in this study are available in the following databases:

- RNA-Seq data: Gene Expression Omnibus GSE46843 (https://www.ncbi.nlm.nih.gov/geo/query/acc.cgi?acc=GSE46843)
- [data type]: [name of the resource] [accession number/identifier/doi] ([URL or identifiers.org/DATABASE:ACCESSION])

*** Note - All links should resolve to a page where the data can be accessed. ***

8) Figure legends and data quantification:
The following points must be specified in each figure legend:
- the name of the statistical test used to generate error bars and P values,
- the number (n) of independent experiments (please specify technical or biological replicates) underlying each data point,
- the nature of the bars and error bars (s.d., s.e.m.)
- If the data are obtained from n {less than or equal to} 2, use scatter blots showing the individual data points.
Discussion of statistical methodology can be reported in the materials and methods section, but figure legends should contain a basic description of n, P and the test applied.
See also the guidelines for figure legend preparation:
https://www.embopress.org/page/journal/14693178/authorguide#figureformat
- Please also include scale bars in all microscopy images.

9) We would also encourage you to include the source data for figure panels that show essential data. Numerical data should be provided as individual .xls or .csv files (including a tab describing the data). For blots or microscopy, uncropped images should be submitted (using a zip archive if multiple images need to be supplied for one panel). Additional information on source data and instruction on how to label the files are available .

10) Our journal encourages inclusion of *data citations in the reference list* to directly cite datasets that were re-used and obtained from public databases. Data citations in the article text are distinct from normal bibliographical citations and should directly link to the database records from which the data can be accessed. In the main text, data citations are formatted as follows: "Data ref: Smith et al, 2001" or "Data ref: NCBI Sequence Read Archive PRJNA342805, 2017". In the Reference list, data citations must be labeled with "[DATASET]". A data reference must provide the database name, accession number/identifiers and a resolvable link to the landing page from which the data can be accessed at the end of the reference. Further instructions are available at .

11) As part of the EMBO publication's Transparent Editorial Process, EMBO reports publishes online a Review Process File to accompany accepted manuscripts. This File will be published in conjunction with your paper and will include the referee reports, your point-by-point response and all pertinent correspondence relating to the manuscript.

You are able to opt out of this by letting the editorial office know (emboreports@embo.org). If you do opt out, the Review Process File link will point to the following statement: "No Review Process File is available with this article, as the authors have chosen not to make the review process public in this case."

We would also welcome the submission of cover suggestions, or motifs to be used by our Graphics Illustrator in designing a cover.

I look forward to seeing a revised form of your manuscript when it is ready. Please use this link to submit your revision:
https://embor.msubmit.net/cgi-bin/main.plex

Please let me know if you have questions or comments regarding the revision.

Yours sincerely,

Martina Rembold, PhD
Senior Editor
EMBO reports

****************************

Referee #1:

Here the authors set out to systematically define protein interactors for the majority of the human RTKs (52/58), using a combination of AP-MS and BioID-MS to identify directly and indirectly interacting or neighboring proteins associated with 52 RTKs that were inducibly expressed in stable cell lines containing a single copy of the transgene, using pervanadate treatment as a surrogate method of activating the expressed RTKs. They identified >6,000 RTK interactions with 1145 identified by AP-MS, 4497 with BioID, and 408 by both methods, with 15 RTKs having >150 interactors. Many of their high confidence interactors (HCIs) had been reported before in one of the public interactome databases, but there were also a significant number of novel interactors. Seventy seven RTK-RTK interactions were identified, with 33 being between receptors in the same subfamily, as might be predicted based on prior findings. Interestingly, EPHA2 was found to interact with 30 RTKs. By CORUM protein complex analysis, they identified 13 PM/ER localized complexes that interact with RTKs, and also 26 nuclear complexes with strictly nuclear components that interact with one of more RTKs, whose identification was unexplained. They also identified 4 HSP90 chaperone complexes, including HSP90/CDC37, a known kinase chaperone complex. In addition, they identified COPII vesicle coatamer subunits, which have not previously been found as RTK interactors, but would be expected based on the endocytic mode of RTK internalization. Screening of the HCIs for modular domains involved in signal transduction, including kinase catalytic domains, combined with GO term analysis revealed components of many of the known RTK-driven signaling pathways. They also used pairwise interactions to assemble protein clusters, and deduced that RTKs have a more direct role in the pathways defined by AP-MS clusters than those defined by BioID, as one might also expect. To identify potential RTK substrates from among the identified HCIs, they used 45 recombinant RTKs to phosphorylate FSBA-treated, 1% NP40 lysates of HEK 293 cells in the presence of18Oγ-ATP, followed by TiO2 phosphopeptide enrichment and MS screening for 18O phosphate-labeled peptides. This led to the identification of ~2,000 pTyr sites, a significant number of which had not been identified before. EPHB1 was found to phosphorylate a massive 982 substrate sites. They examined the EGFR interactome in detail, finding 96 known interactors and 137 novel interactors. They also assessed the differences in the interactomes of 11 wild-type and kinase-dead RTK mutant pairs, including AXL and EPHA7, and found significant differences, with WT-specific interactors being deduced to be ligand-dependent interactions. Finally, as an example of an understudied RTK, the EPHA7 interactome and its substrates were examined in more detail.

The system-wide RTK interactome data that have been amassed will undoubtedly be of value to the signaling community. Many novel RTK interactions were identified, but apart from the RTK-RTK interactions, a weakness is that there was little additional validation of any individual novel RTK interactors, nor any investigations into whether any novel interactions are functionally relevant in signaling responses downstream of the RTK in question. Moreover, as indicated below, there some caveats about the approaches used that raise some concerns about the extent to which the novel interacts are truly physiological, including the fact that many of the RTKs that were expressed in Flp-In-293 T-REX cells are not expressed endogenously in the parental HEK 293 epithelial cells (or in any epithelial cells), and are therefore in a "foreign" environment, and that pervanadate-mediated RTK activation, while expedient, is not truly physiological. It would strengthen the paper if some validation of one or two of the novel RTK interactors to demonstrate their physiological relevance had been had included, although this is a resource style paper, which typically do not have much in the way of validation. Nevertheless, technically this is a tour de force, and its publication will allow other people in the field to pick and choose interesting interactors for their RTK of interest for further functional analysis.

With regard to what fraction of the identified RTK interactors are physiologically relevant, the following are some caveats to the approaches used that were not adequately discussed.

Points: 1. The authors need to make it clear at the start of the Results what parental cell line was used for exogenous RTK expression. This is important, because in order to evaluate whether the observed RTK interactions are likely to be physiological, one needs to know which of the exogenously expressed RTKs are normally expressed endogenously in this cell line. As indicated in the Methods, they generated stable RTK-expressing cell lines using Flp-In-293 T-REX cells, which derived from HEK293 cells, a transformed human embryonic kidney cell of epithelial origin. This means that the neural, endothelial and mesenchmyal cell-specific RTKs expressed here were not in a native environment. The authors did analyze the Human Protein Atlas for expression of RTK proteins in HEK 293 cells (Table S1B), but only 36 RTKs are listed in the table, and 13 of these RTKs are expressed at negligible levels. It would be helpful if the authors generated a list of all 52 RTKs they tested, indicated which RTK isoforms were expressed in the Flp-In-293 T-REX cell line (many RTKs have important alternatively spliced isoforms), and which of the 52 RTKs are expressed at the protein (and possibly RNA level) in HEK 293 cells. While they did find that many of the unique interactors were expressed in many different types of cell line, key cell specific interactors will likely have been missed. Moreover, the heterotypically expressed RTKs may not have been correctly localized to plasma membrane subdomains due to lack of required localization partners, and may even have accumulated in internal membrane compartments.

2. Rather than using ligands to activate the expressed RTKs, the authors treated 24-h TET-induced cells with pervanadate for 15 min prior to harvesting for AP-MS and BioID-MS analysis. Given that ligands for all 52 RTKs are not readily available, this is an expedient approach, but how physiological the cell's response is to pervanadate activation is questionable. The authors did carry out a pTyr proteomic analysis to check which RTK autophosphorylation sites were phosphorylated in pervanadate-treated cells (Table S2), but apparently only compared them with known sites of autophosphorylation, e.g. in PhosphoSItePlus, and did not look for novel sites of RTK Tyr phosphorylation. Obviously, some (many) of the pervanadate-induced sites are those also induced by RTK ligand binding, but pervanadate treatment could also induce aberrant Tyr phosphorylation (e.g. RTK dimers are not formed). This is an important issue because, at least in the case of the EGFR, phosphates on the key Tyr residues turn over several times a minute in EGF-treated cells. PTP activity blockade abrogates this process, and this allows accumulation of pTyr at greater than physiological levels on physiological sites, but may also permit phosphate to accumulate on Tyr residues that are not normally autophosphorylated to a significant extent, which could induce non-physiological RTK-protein interactions. In their in vitro kinase analysis, the authors also identified a number of novel pTyr substrate sites (Figure 5D), which might also be "artifacts" of PTP inhibition (or lysate admixture). The authors did carry out subsequent tests for eight RTKs, EGFR, FGFR1, FGFR4, IGF1R, INSR, INSRR, PDGFRB, and RET, activated by treatment with EGF, FGF, IGF, HGF, NT-3; PDGF-BB, and GDNF, respectively, finding on average about 80% of the interactors observed with pervanadate treatment, but this does leave a significant number of potentially non-physiological interactors.

3. They authors identified a number of RTK-RTK interactions by both AP-MS and BioID-MS approaches (Figure 2A), and validated 27 of them by co-IP. However, since they did not use ligand treatment, which is usually required to induce or stabilize RTK dimerization prior to activation, this number may be a significant underestimate (or overestimate, if all these interactions relied on pervanadate activation, which might not be expected to generate physiological RTK dimers.) Also, pervanadate treatment and inhibition of PTPs will lead to activation of non-receptor tyrosine kinases, which could result in phosphorylation of RTKs themselves and as well as other proteins leading to indirect association with RTKs.

4. Apart from the relatively non-physiological nature of pervanadate stimulation, the AP-MS protocol is reasonable. However, there are some questions about the BioID-MS protocol. Apparently, biotin was added at the time of RTK induction 24 h prior to BioID-MS analysis, with pervanadate being added for the last 15 min prior to harvesting. This means that most of the biotinylated proteins identified will have been proteins biotinylated because of their close proximity to the RTK in question when it was in the unactivated state. Indeed, the 15 min period when the RTK was active seems unlikely to have been long enough to accumulate many ligand-dependent interactor proteins that will have been biotinylated - it would have been better to use TurboID for this purpose. Some further discussion of this issue is called for.

Point 1: Figure 1A: The authors need to tell us which RTKs they did not test, and why. In addition, five RTKs (ERBB3, PTK7/CCK4, EPHB6, EPHA10 and STYK1/SuRTK106) are designated as pseudokinases, and lack significant kinase activity; ROR1, ROR2, and RYK may also lack kinase activity. The authors need to indicate this, and discuss how this might affect their interpretations, possibly in the context where they compare the interactomes of WT and kinase-dead RTK mutants in Figure 6. In this connection, they should note that LMR1-3, while lying on the TK branch of the kinome, are no longer classified as TKs, but rather Ser/Thr kinases (recombinant LMR "RTKs" were not tested in vitro to determine whether any LMR-mediated substrate pTyr sites could be identified, and no LMR pTyr autophosphorylation sites were reported).

2. It would help the uninitiated reader if the authors described the structure or the RTK-MACTAG-C expression vector in the text at the start of the Results.

3. Page 5/Table S3: It is possible that some of the observed RTK-nuclear complex interactions identified by AP-MS occurred post lysis (obviously this is not an issue with BioID-MS).

4. Figure 5: The use of 18Oγ-ATP for the in vitro kinase activity assay is clever, but, even if one disregards the cell specificity issue, there are several reasons to query how representative and physiological the catalogue of identified RTK "substrates" is. First, it seems unlikely that all of the relevant substrate proteins will have been released from cellular membranes into a 16K xg supernatant by a 1% NP40 lysis protocol. Second, TiO2 enrichment is not as efficient as the use of a pTyr mAb or super-SH2 domain phosphopeptide pull down for identification of pTyr-containing peptides. Third, the process of cellular lysis and the use of a high concentration of active recombinant RTK will bring together the RTK in question with many proteins that it will never normally encounter in the cell, i.e. the normal milieu for RTK-mediated phosphorylation is the submembraneous compartment. Thus, while a number of authentic pTyr sites were identified, it is unclear how many of the other pTyr sites in their list of ~2,000 are likely to be physiologically relevant. In this connection, based on the one RTK I searched for at Life Technologies, it appears that the recombinant proteins used in these experiments were GST-fusions of the RTK cytoplasmic domain rather than full length EGFR protein, and for this reason the nature and catalogue numbers of the recombinant RTKs used needs to be indicated in the Methods.

5. Figure 7/pages 10 and11: This section is devoted to the analysis of the EPHA7 interactome, which was characterized because EPHA7 is an understudied RTK. However, the authors should point out as a caveat that EPHA7 is not expressed endogenously in HEK 293 cells.

6. The authors could mention the use of the MaMTH split Ub system as another method for identifying functional interactors with RTKs, e.g. Yao et al. PMID: 28065597; Aboualizadeh et al. PMID: 34606829.

Referee #2:

In this draft manuscript, Salokas et al describe a tour de force characterisation of the interactome of receptor tyrosine kinases (RTK). They expressed all RTKs in Hek293 cells and performed both affinity pulldowns and Bio-ID. Moreover, for a large number of RTKs they also performed in vitro phosphorylation.

The manuscript serves as an excellent resource for anyone interested in RTKs. The wealth of the data will be of great interest to a lot of people and the authors should be lauded for their hard work. However, the huge amount of data also makes reading of the manuscript very difficult as actually very little biology is explained. It may have been better if the authors had split up some of the data and had tried to explain more of the biological findings in detail. In many figures it is not clear to me to which RTKs the shown complexes are actually binding.
The authors are aware and mention the deficits of their work (use of HEK293 cells which may not express all interactors; expression artefacts). They may want to add that tags on the C-terminus may also affect binding partners. This is in no way a criticism as it is more a technical limitation, however, it should be discussed.

Minor points:
1. The figure legends are too short and do often not explain the whole data shown making it very difficult to understand the figures. For example, Figure 1F or figure 6D. Please also show individual data points in figure 6D. Figure 3C: what are the log2 values to what are the normalised?
2. I understand the difficulty of showing such complex data, but some of the figures will not provide much of insight as they are just too complex. A good example for this is Figure 2B.
3. Please put all supplementary figures into one file with legends beneath the figures.
4. Molecular weight markers are missing in Figure S1E

Referee #1:

Here the authors set out to systematically define protein interactors for the majority of the human RTKs (52/58), using a combination of AP-MS and BioID-MS to identify directly and indirectly interacting or neighboring proteins associated with 52 RTKs that were inducibly expressed in stable cell lines containing a single copy of the transgene, using pervanadate treatment as a surrogate method of activating the expressed RTKs. They identified >6,000 RTK interactions with 1145 identified by AP-MS, 4497 with BioID, and 408 by both methods, with 15 RTKs having >150 interactors. Many of their high confidence interactors (HCIs) had been reported before in one of the public interactome databases, but there were also a significant number of novel interactors. Seventy seven RTK-RTK interactions were identified, with 33 being between receptors in the same subfamily, as might be predicted based on prior findings. Interestingly, EPHA2 was found to interact with 30 RTKs. By CORUM protein complex analysis, they identified 13 PM/ER localized complexes that interact with RTKs, and also 26 nuclear complexes with strictly nuclear components that interact with one of more RTKs, whose identification was unexplained. They also identified 4 HSP90 chaperone complexes, including HSP90/CDC37, a known kinase chaperone complex. In addition, they identified COPII vesicle coatamer subunits, which have not previously been found as RTK interactors, but would be expected based on the endocytic mode of RTK internalization. Screening of the HCIs for modular domains involved in signal transduction, including kinase catalytic domains, combined with GO term analysis revealed components of many of the known RTK-driven signaling pathways. They also used pairwise interactions to assemble protein clusters, and deduced that RTKs have a more direct role in the pathways defined by AP-MS clusters than those defined by BioID, as one might also expect. To identify potential RTK substrates from among the identified HCIs, they used 45 recombinant RTKs to phosphorylate FSBA-treated, 1% NP40 lysates of HEK 293 cells in the presence of18Oγ-ATP, followed by TiO2 phosphopeptide enrichment and MS screening for 18O phosphate-labeled peptides. This led to the identification of ~2,000 pTyr sites, a significant number of which had not been identified before. EPHB1 was found to phosphorylate a massive 982 substrate sites. They examined the EGFR interactome in detail, finding 96 known interactors and 137 novel interactors. They also assessed the differences in the interactomes of 11 wild-type and kinase-dead RTK mutant pairs, including AXL and EPHA7, and found significant differences, with WT-specific interactors being deduced to be ligand-dependent interactions. Finally, as an example of an understudied RTK, the EPHA7 interactome and its substrates were examined in more detail.

The system-wide RTK interactome data that have been amassed will undoubtedly be of value to the signaling community. Many novel RTK interactions were identified, but apart from the RTK-RTK interactions, a weakness is that there was little additional validation of any individual novel RTK interactors, nor any investigations into whether any novel interactions are functionally relevant in signaling responses downstream of the RTK in question. Moreover, as indicated below, there some caveats about the approaches used that raise some concerns about the extent to which the novel interacts are truly physiological, including the fact that many of the RTKs that were expressed in Flp-In-293 T-REX cells are not expressed endogenously in the parental HEK 293 epithelial cells (or in any epithelial cells), and are therefore in a "foreign" environment, and that pervanadate-mediated RTK activation, while expedient, is not truly physiological. It would strengthen the paper if some validation of one or two of the novel RTK interactors to demonstrate their physiological relevance had been had included, although this is a resource style paper, which typically do not have much in the way of validation. Nevertheless, technically this is a tour de force, and its publication will allow other people in the field to pick and choose interesting interactors for their RTK of interest for further functional analysis.

*We thank the reviewer for very positive feedback and comments. We also agree with him/her with the notion "The system-wide RTK interactome data that have been amassed will undoubtedly be of value to the signaling community" and that "this is a tour de force, and its publication will allow other people in the field to pick and choose interesting interactors for their RTK of interest for further functional analysis".*

*We've now added more in-depth discussion about RTK expression in cell lines, and the rationale behind choosing HEK293 Flp-In T-REx for the experiments in the beginning of the results section (Result section*

*“Defining the RTK interaction landscape”, second paragraph): “HEK293 cell line is one of the cell lines expressing the highest number of RTKs, according to the human protein atlas project (Thul and Lindskog, 2018). When we investigated, which cell lines had any detectable expression of the RTKs included in this study, only three cell lines had more RTKs expressed than HEK293: SCLC-21H, NTERA-2, and U-2 OS with 50, 48, and 46 RTKs expressed, respectively, compared to the 43 of HEK293. After filtering the values with normalized transcript expression value (nTPM) of 1, 34 RTKs passed the filter in HEK293 cells, while U-2 OS had 36 RTKs, and SCLC-21H and NTERA-2 had 31 and 34, respectively. We next investigated the data of the human cell map project for protein-level RTK expression detection. The project utilized the HEK293 Flp-In T-Rex cell line to generate a proximity biotinylation map of the human cell(Go et al., 2021). While the project makes no attempt to characterize the expression levels of any protein family, in their data, we found 26 RTKs as preys (signifying endogenous expression), of which three (INSRR, TEK, EphA1) were not detected in the protein atlas HEK293 data at all, and further three (RON, RET, DDR2) were detected with nTPM values smaller than 1. Taken together, the expression data from the protein atlas and protein-level evidence from the human cell map, HEK293 is one of the more RTK-rich cell lines available, and perhaps the richest, if only considering cell lines for which inducible, isogenic expression systems are available.”*

*We’ve also performed 83 Co-IP experiments to validate AP-MS interactors (Dataset EV1C), and of these 69 were positive. The 14 negative ones may also represent protein complexes, i.e. interactions mediated by a third protein, as the AP-MS approach does not attempt to identify whether the interactions detected are direct, or whether they represent a protein complex.*

With regard to what fraction of the identified RTK interactors are physiologically relevant, the following are some caveats to the approaches used that were not adequately discussed.
Points: 1. The authors need to make it clear at the start of the Results what parental cell line was used for exogenous RTK expression. This is important, because in order to evaluate whether the observed RTK interactions are likely to be physiological, one needs to know which of the exogenously expressed RTKs are normally expressed endogenously in this cell line. As indicated in the Methods, they generated stable RTK-expressing cell lines using Flp-In-293 T-REX cells, which derived from HEK293 cells, a transformed human embryonic kidney cell of epithelial origin. This means that the neural, endothelial and mesenchmyal cell-specific RTKs expressed here were not in a native environment. The authors did analyze the Human Protein Atlas for expression of RTK proteins in HEK 293 cells (Table S1B), but only 36 RTKs are listed in the table, and 13 of these RTKs are expressed at negligible levels. It would be helpful if the authors generated a list of all 52 RTKs they tested, indicated which RTK isoforms were expressed in the Flp-In-293 T-REX cell line (many RTKs have important alternatively spliced isoforms), and which of the 52 RTKs are expressed at the protein (and possibly RNA level) in HEK 293 cells. While they did find that many of the unique interactors were expressed in many different types of cell line, key cell specific interactors will likely have been missed. Moreover, the heterotypically expressed RTKs may not have been correctly localized to plasma membrane subdomains due to lack of required localization partners, and may even have accumulated in internal membrane compartments.

*We have now added information about the cell line to the beginning of the results section (see previous comment). Furthermore, we have added a characterization of RTK expression in HEK293 cell lines, reanalyzed the ProteinAtlas cell line RNA data, and compared RTK expression to other cell lines in the beginning of the results section. We used data from ProteinAtlas to compare cell lines, and further identified expressed RTKs from a large-scale BioID interactomes of non-RTK baits from the cellular biotinylation map project from Gingras lab (PMID: 34079125). As a result we identify HEK293 as one of the most “RTK-rich” cell lines available, especially if only considering cell lines for which isogenic inducible and adjustable expression systems such as Flp-In T-Rex are available. We agree that some cell type-specific interactions might be missed, which is always the possibility in any biological research. We have now added this possible limitation to the discussion. Regarding the possibility that some of the RTKs might not have been localized correctly to specific plasma membrane subdomains, this exists. We do show that the RTKs localize correctly to plasma membrane, however, the resolution of a standard confocal microscopy does not really allow*

*analysis of specific membrane subdomains. In principle, the MS microscopy approach used by us could work, however we failed to find existing systematic information about the RTKs localization to specific plasma membrane subdomains, and could not really assess this. We have now added a sentence about this lack of knowledge about the RTK localization to a specific plasma membrane subdomains to the discussion: "Similarly, we cannot be certain that the RTKs localize to the correct plasma membrane subdomains, as systemic information about this detail of RTK behavior is not available."*

2. Rather than using ligands to activate the expressed RTKs, the authors treated 24-h TET-induced cells with pervanadate for 15 min prior to harvesting for AP-MS and BioID-MS analysis. Given that ligands for all 52 RTKs are not readily available, this is an expedient approach, but how physiological the cell's response is to pervanadate activation is questionable. The authors did carry out a pTyr proteomic analysis to check which RTK autophosphorylation sites were phosphorylated in pervanadate-treated cells (Table S2), but apparently only compared them with known sites of autophosphorylation, e.g. in PhosphoSItePlus, and did not look for novel sites of RTK Tyr phosphorylation. Obviously, some (many) of the pervanadate-induced sites are those also induced by RTK ligand binding, but pervanadate treatment could also induce aberrant Tyr phosphorylation (e.g. RTK dimers are not formed). This is an important issue because, at least in the case of the EGFR, phosphates on the key Tyr residues turn over several times a minute in EGF-treated cells. PTP activity blockade abrogates this process, and this allows accumulation of pTyr at greater than physiological levels on physiological sites, but may also permit phosphate to accumulate on Tyr residues that are not normally autophosphorylated to a significant extent, which could induce non-physiological RTK-protein interactions. In their in vitro kinase analysis, the authors also identified a number of novel pTyr substrate sites (Figure 5D), which might also be "artifacts" of PTP inhibition (or lysate admixture). The authors did carry out subsequent tests for eight RTKs, EGFR, FGFR1, FGFR4, IGF1R, INSR, INSRR, PDGFRB, and RET, activated by treatment with EGF, FGF, IGF, HGF, NT-3; PDGF-BB, and GDNF, respectively, finding on average about 80% of the interactors observed with pervanadate treatment, but this does leave a significant number of potentially non-physiological interactors.

*The 15 minute pervanadate activation time in BioID experiments is an important point. When the experiments were started, rapid methods such as turboID (PMID: 31748691) or the even better ultraID (preprint, DOI: 10.1101/2021.06.16.448656) were not yet available, and in the case of turboID, were not available as combination tags that would allow us to perform AP-MS from the same cell line (in an effort to reduce both the variation between the conditions in AP-MS and BioID experiments). Initially we were not convinced that the 15 minute pervanadate activation would be needed for the BioID experiments, as it is for the AP-MS. As the biotinylation for the BioID was already 24 hours, it is expected that the majority of the RTKs will become activated and signal correctly during this time - and during this period biotinylate the interacting and proximal proteins. However, we performed a pilot experiment with NTRK3 and could see additional pervanadate induction specific and biologically relevant interactions appearing. Therefore, to comprehensively see the majority of RTK interactions, we decided to use the pervanadate induction also for the BioID experiments. However, as partly initiated by the reviewer, we generated an ultraID version of the MAC-tag and performed the comparison between our used BioID protocol and the ultraID protocol. In this experiment we compared untreated and pervanadate-activated NTRK3 BioID results with that of the ultraID. This data suggests that the our used BioID approach compares with the new ultraID.*

*In general, unfortunately ligands for many RTKs are either unknown, unavailable, or not feasible to use in a systematic and a gene-family large analysis such as ours. We have now added the NTRK3 BioID and ultraID comparison (Result section "Defining the RTK interaction landscape", twelfth paragraph), discussed the ligand- or pervanadate-only interactors in more detail (Result section "Defining the RTK interaction landscape", eleventh paragraph), and have also added a new dataset (Dataset EV1D) to identify which interactors the pervanadate treatment might miss compared to specific ligand. In the results, we detected the patterns that while both ligand and pervanadate treatment identifies some treatment-specific interactors, both groups had a highly similar number of previously known interactions. In particular with RTK-RTK interactions, the only pervanadate-only RTK-RTK interactions we identified (FGFR1-FGFR2, and EGFR-EphA2) were previously documented. Currently, the differences between pervanadate and ligand treatment*

*likely stem from the treatment itself, but possibly also from batch effect bias of high-throughput experiments, such as slight variations in cell culture conditions and, in particular with interactions identified with low spectral count values, from variations in affinity purification, desalting, and mass spectrometry steps.*

*Therefore, while it is impossible to obtain even half complete specific ligand-induced RTK interactome with any experimental strategy, we feel confident that the interactions we identify in this study using pervanadate activation and after stringent filtering represent high-confidence interactions, and can be expected to occur in similar biological conditions.*

3. They authors identified a number of RTK-RTK interactions by both AP-MS and BioID-MS approaches (Figure 2A), and validated 27 of them by co-IP. However, since they did not use ligand treatment, which is usually required to induce or stabilize RTK dimerization prior to activation, this number may be a significant underestimate (or overestimate, if all these interactions relied on pervanadate activation, which might not be expected to generate physiological RTK dimers.) Also, pervanadate treatment and inhibition of PTPs will lead to activation of non-receptor tyrosine kinases, which could result in phosphorylation of RTKs themselves and as well as other proteins leading to indirect association with RTKs.

*Based on ligand-treatment and pervanadate comparison data (Figure 1F, Dataset EV1D), pervanadate treatment does not cause unphysiological RTK-RTK interactions (discussed in the first paragraph of "Kinase-kinase interactions between RTKs" -section). However, it is possible that not all interactions between RTKs and RTKs, or RTKs and other proteins, are induced to occur with pervanadate or can be detected by the analysis approach we were applied. It is also possible that not all RTK interactions can occur in cell culture conditions and would require an organoid-type cell culture experimental setup. We have now added a brief discussion about the future directions of interactome analyses toward organoids: "Future improvements on understanding RTK behavior as a part of a more complete model system might require both more information about the specific RTK membrane substructure localization, and perhaps using 3D organoid cell culture techniques to better imitate the 3D tissue structure, within which RTKs physiologically function."*

4. Apart from the relatively non-physiological nature of pervanadate stimulation, the AP-MS protocol is reasonable. However, there are some questions about the BioID-MS protocol. Apparently, biotin was added at the time of RTK induction 24 h prior to BioID-MS analysis, with pervanadate being added for the last 15 min prior to harvesting. This means that most of the biotinylated proteins identified will have been proteins biotinylated because of their close proximity to the RTK in question when it was in the unactivated state. Indeed, the 15 min period when the RTK was active seems unlikely to have been long enough to accumulate many ligand-dependent interactor proteins that will have been biotinylated - it would have been better to use TurboID for this purpose. Some further discussion of this issue is called for.

*The reviewer raises a good point about the BioID data representing proteins over the 24h lifetime of each RTK, rather than only at the moment of activation. This actually works to our benefit, as we are interested in the complete RTK interactomes. As the biotinylation time for the BirA* (R118G, BioID) was already 24 hours, it is expected that the majority of the RTKs will become activated and signal correctly during this time - and during this period biotinylate the interacting and proximal proteins. However, a pilot experiment taking NTRK3 as example, the addition of pervanadate facilitated the identification of certain specific and biologically relevant interactions (e.g. PLCG1 and GRB2). Therefore, to comprehensively map the majority of RTK interactions, we decided to use the pervanadate induction also for the BioID approach. Furthermore, an ultraID version of the MAC-tag which contains Strep, HA and UltraID tags was generated and applied to compare the BioID protocol (24 h biotinylation) with the ultraID protocol (10 min biotinylation). Multiple comparisons were conducted between untreated and pervanadate-activated NTRK3 tagged with either BioID or ultraID versions of MAC-tag. This data suggests that these two different versions of MAC-tag perform similar labeling effects as majority interactions (over 90 %) were identified by both BioID and UltraID.The new MAC-tag versions with the ultraID are made available via Addgene, and we have added some text about the advantages of this new MAC-tag to the discussion. Of note is that the MAC-tag-ultra is still in development, and might require further optimization in both experimental*

*conditions and data analysis. We are currently working on a larger own study to validate this new version of the MAC-tag.*

Point 1: Figure 1A: The authors need to tell us which RTKs they did not test, and why. In addition, five RTKs (ERBB3, PTK7/CCK4, EPHB6, EPHA10 and STYK1/SuRTK106) are designated as pseudokinases, and lack significant kinase activity; ROR1, ROR2, and RYK may also lack kinase activity. The authors need to indicate this, and discuss how this might affect their interpretations, possibly in the context where they compare the interactomes of WT and kinase-dead RTK mutants in Figure 6. In this connection, they should note that LMR1-3, while lying on the TK branch of the kinome, are no longer classified as TKs, but rather Ser/Thr kinases (recombinant LMR "RTKs" were not tested in vitro to determine whether any LMR-mediated substrate pTyr sites could be identified, and no LMR pTyr autophosphorylation sites were reported).

*We thank the Review for pointing this out. We have now added discussion for the rationale for including both pseudokinases and the LMR family in the analysis in the fourth paragraph of the results section: "Although the data includes five pseudokinases (ERBB3, EphB6, EphA10, STYK1) and others, which are suspected to be pseudokinases (ROR1, ROR2, RYK), they were included in the study for data completeness. For the same reason, we have included the LMR family in the analysis. Although LMRs have historically been associated with the RTK family (Butti et al., 2018; Lemmon and Schlessinger, 2010), they have recently been removed from the receptor family (Trenker and Jura, 2020; Wendler et al., 2021), and classified as serine/threonine kinases."*

*Unfortunately, UniProt does not contain information about any autophosphorylated sites in any of the three LMR subfamily members, and thus we cannot conclude whether we identify possibly autocatalyzed sites or not.*

2. It would help the uninitiated reader if the authors described the structure or the RTK-MACTAG-C expression vector in the text at the start of the Results.

*A very good suggestion! We added a description of what the MAC-tag consists of in the third paragraph of the results for clarity. :"For all RTKs, a C-terminal MAC-tag was used, in order to ensure tagging of intracellular interactors in BioID experiments. The tag consists of two Strep-Tag II sequences, followed by HA, and finally the BirA* enzyme with linker sequences in between."*

3. Page 5/Table S3: It is possible that some of the observed RTK-nuclear complex interactions identified by AP-MS occurred post lysis (obviously this is not an issue with BioID-MS).

*This is a good point. As the AP-MS purification were performed in short time period at 4 °C (PMID: 32778839), this is quite unlikely. However, we cannot exclude the possibility, although relatively unlikely, source of these interactions: "While it is possible that some nuclear interactions detected via AP-MS could stem from binding post-lysis in ice-cold conditions (though unlikely), and some interactions detected via either method can be proteins encountered only during mitosis after nuclear breakdown, the data may also offer some additional context for possible connections between RTKs and nuclear signaling pathways."*

4. Figure 5: The use of 18Oγ-ATP for the in vitro kinase activity assay is clever, but, even if one disregards the cell specificity issue, there are several reasons to query how representative and physiological the catalogue of identified RTK "substrates" is. First, it seems unlikely that all of the relevant substrate proteins will have been released from cellular membranes into a 16K xg supernatant by a 1% NP40 lysis protocol. Second, TiO2 enrichment is not as efficient as the use of a pTyr mAb or super-SH2 domain phosphopeptide pull down for identification of pTyr-containing peptides. Third, the process of cellular lysis and the use of a high concentration of active recombinant RTK will bring together the RTK in question with many proteins that it will never normally encounter in the cell, i.e. the normal milieu for RTK-mediated phosphorylation is the submembraneous compartment. Thus, while a number of authentic pTyr sites were identified, it is unclear how many of the other pTyr sites in their list of ~2,000 are likely to be physiologically relevant. In

this connection, based on the one RTK I searched for at Life Technologies, it appears that the recombinant proteins used in these experiments were GST-fusions of the RTK cytoplasmic domain rather than full length EGFR protein, and for this reason the nature and catalogue numbers of the recombinant RTKs used needs to be indicated in the Methods.

*Phosphopeptide enrichment was performed with Titanium (IV) ion ($Ti^{4+}$-IMAC), and not $TiO_2$, for which the material was prepared according to the protocol published by Zhou et al., 2013 (PMID: 23391890). We have now also added a reagents and tools table, which contains all catalog numbers for the used recombinant proteins. As the reviewer pointed out, the recombinant kinases do not include the extracellular parts, and we have now added text in the beginning of the IVK section of the results. We have also added remarks about four of the analyzed RTKs, which were missing a small part of their kinase domain: "Another important consideration is that the recombinant kinases available do not include the extracellular domains of RTKs. In total, forty-five recombinant RTKs were used for experiments that included all RTK subfamilies. Of these, four kinases were missing one or more amino acids from the end of their kinase domain: NTRK3 was missing 14, FGFR1 was missing 36, DDR1 29, and EphB1 was missing one. "*

5. Figure 7/pages 10 and11: This section is devoted to the analysis of the EPHA7 interactome, which was characterized because EPHA7 is an understudied RTK. However, the authors should point out as a caveat that EPHA7 is not expressed endogenously in HEK 293 cells.

*We have now added text about this to the beginning of the corresponding section: "Although EphA7 is not expressed in HEK293 cells according to the protein atlas (Uhlén et al., 2015), it was seen to be endogenously expressed in the data of CellMap(Dataset EV1B, Go et al., 2021)."*

6. The authors could mention the use of the MaMTH split Ub system as another method for identifying functional interactors with RTKs, e.g. Yao et al. PMID: 28065597; Aboualizadeh et al. PMID: 34606829.

*We thank the reviewer for the comments. We have now added a sentence about these two papers from the Stagljar lab to discussion: "Although methods such as membrane yeast two-hybrid and mammalian membrane two-hybrid have been applied to study facets of RTK interactions, such as RTK-phosphatase relationships (Yao et al., 2017) or individual RTKs (Aboualizadeh et al., 2021) with success, no systemic, global mapping of interactions has previously been published."*

Referee #2:

In this draft manuscript, Salokas et al describe a tour de force characterisation of the interactome of receptor tyrosine kinases (RTK). They expressed all RTKs in Hek293 cells and performed both affinity pulldowns and Bio-ID. Moreover, for a large number of RTKs they also performed in vitro phosphorylation.

The manuscript serves as an excellent resource for anyone interested in RTKs. The wealth of the data will be of great interest to a lot of people and the authors should be lauded for their hard work. However, the huge amount of data also makes reading of the manuscript very difficult as actually very little biology is explained. It may have been better if the authors had split up some of the data and had tried to explain more of the biological findings in detail. In many figures it is not clear to me to which RTKs the shown complexes are actually binding.
The authors are aware and mention the deficits of their work (use of HEK293 cells which may not express all interactors; expression artefacts). They may want to add that tags on the C-terminus may also affect binding partners. This is in no way a criticism as it is more a technical limitation, however, it should be discussed.

*We thank the reviewer for very positive feedback and comments. Especially for the comments that "The manuscript serves as an excellent resource for anyone interested in RTKs. The wealth of the data will be of great interest to a lot of people and the authors should be lauded for their hard work."*

*The C-terminal tagging approach was chosen due to extracellular location of the RTK N-termini at the cell membrane. We have now added text about technical advantages and limitations of using tag-approach in interaction proteomics: "While it is possible, that the C-terminal tag might affect protein binding with some partners, this likely only affects the AP-MS experiments, and moving the tag to the N-terminus would pose major problems to the BioID experiments, because the N-terminus of RTKs is extracellular."*

Minor points:
1. The figure legends are too short and do often not explain the whole data shown making it very difficult to understand the figures. For example, Figure 1F or figure 6D. Please also show individual data points in figure 6D. Figure 3C: what are the log2 values to what are the normalised?

*Individual data points are now visible in figure 6D, and all figure legends have been expanded with additional information. These were kept short due to space constraints. Figure 3C legend has been clarified in regard to the log2 fold change values, which were obtained using the human UniProt database as reference, and similar details have been added to other figure legends, where appropriate.*

2. I understand the difficulty of showing such complex data, but some of the figures will not provide much of insight as they are just too complex. A good example for this is Figure 2B.

*We do agree with the occasional difficulty showing complex data, and agree that the Figure 2 is somewhat busy and the current form of 2B was too small and condensed. We have therefore decided to sinplify the Figure 2 and move the Figure 2B as its own expanded view figure EV4.*

3. Please put all supplementary figures into one file with legends beneath the figures.

*We have now divided the supplementary figures into five expanded view (EV) figures, and put the remaining figures into the appendix with legends, as suggested.*

4. Molecular weight markers are missing in Figure S1E

*The figure has now been renamed figure EV1, and the molecular weight markers have been added to the panel in question (EV1E).*

**1st Revision - Editorial Decision** **28th Feb 2022**

Dear Dr. Varjosalo

Thank you for the submission of your revised manuscript to EMBO reports. We have now received the report from the referee who was asked to assess it (copied below).

As you will see, referee 1 is very positive about the study and requests only minor changes to clarify text, figures and methods. Please address the remaining concerns in the manuscript and in a point-by-point response. Please also add molecular weight markers to Figure EV1E, as requested by referee 2 in the first round of review.

From the editorial side, there are also a few things that we need before we can proceed with the official acceptance of your study.

- We would like to publish your study in the Resource section of EMBO Reports and I have therefore changed the article type from "Article" to "Resource".

- Please add up to five keywords.

- Funding: The information listed in the manuscript does not match with the funding info supplied in our online submission system. Please double-check and correct this information. Please also note that the funding information should be included in the Acknowledgements section.

- Appendix: Please add a/the title and page numbers to the table of content.

- I noticed that you mention a Reagents and Tools table in the Author Checklist but have not supplied such a table. Please either update the information in the Checklist or provide the table in case it is missing.

- Data availability section: Please add links that resolve to the datasets on MissIVE. Please remove the reviewer tokens and please remove the sentence "Plasmids are available from the corresponding author upon reasonable request" since this section is only meant to provide information on publicly deposited datasets.

- Please order the manuscript sections following the Discussion as follows:
Materials and Methods
Data availability
Acknowledgements
Author contributions
Disclosure and competing interests statement
References
Figure legends
Expanded View Figure legends

- During our routine image analysis that we perform on all manuscripts prior to publication, we noted the blots shown have rather high contrast settings (Fig. EV1D, EV1E, EV5D). Please apply as little contrast/brightness modification as possible and please also supply the unmodified source data for these blots upon resubmission.

- I attach to this email a related manuscript file with comments by our data editors (plus some minor changes to the abstract). Please address all comments and upload a revised file with tracked changes with your final manuscript submission.

- Finally, EMBO reports papers are accompanied online by A) a short (1-2 sentences) summary of the findings and their significance, B) 2-3 bullet points highlighting key results and C) a synopsis image that is 550x200-600 pixels large (width x height) in .png format. You can either show a model or key data in the synopsis image. Please note that the size is rather small and that text needs to be readable at the final size. Please send us this information along with the revised manuscript.

We look forward to seeing a final version of your manuscript as soon as possible.

With kind regards,

Martina Rembold, PhD
Senior Editor
EMBO reports

************************

Referee #1:

The authors have addressed the reviewers' concerns, in part through addition of some further co-IP validation data in Figure EV2B (although these were transient overexpression experiments), and also inclusion of some new RTK localization IF data added in Figure EV2A. Overall this is improved, although since HEK293 cells are transformed by AdV5 E1A/E1B and of embryonic origin, and a significant number of the RTKs they express may not be physiologically relevant. Also, it seems unlikely that most of these RTKs are functionally activated when HEK293 cells are grown in culture, as suggested, since this would require expression of the cognate ligands for autocrine stimulation. Nonetheless, as a whole this RTK interactome dataset should be useful to the RTK community.

1. Figure EVF2A: This new experiment needs a more detailed description. It is really hard to tell from these single cell images (how representative are they?), what fraction of the expressed RTK was at the plasma membrane; in some cases, there is what seems to be plasma membrane localization, but in other it appears that most of the RTK protein was intracellular, possibly in vesicular structures, which is likely to allow nonphysiological interactions. What one would really like to see is the comparative localizations of the tagged RTK and the endogenous RTK (for an RTK for which a good anti-RTK IF antibody is available), but this would be beyond the scope of the paper. Theis issue deserves a more extensive discussion than it gets on page 4.

2. Figure EVF2B: The legend needs to describe what was done, and in particular which anti-tag antibody was used for IP and which for blotting the dotted samples - this is not even clear in the new section of the methods describing the dot blotting procedure.

3. Page 5/Figure EV3: There is no description of or citation for UltraID in the text legend or methods (there is some information in the rebuttal, but this needs to be in the paper), which makes it impossible to understand what comparison was carried out for NTRK3 and what it means! This needs to be remedied.

*Dear Dr. Varjosalo*

*Thank you for the submission of your revised manuscript to EMBO reports. We have now received the report from the referee who was asked to assess it (copied below).*

*As you will see, referee 1 is very positive about the study and requests only minor changes to clarify text, figures and methods. Please address the remaining concerns in the manuscript and in a point-by-point response. Please also add molecular weight markers to Figure EV1E, as requested by referee 2 in the first round of review.*

We apologize for the missing molecular weight markers in the previous submission, and have now added them to the current version.

*From the editorial side, there are also a few things that we need before we can proceed with the official acceptance of your study.*

*- We would like to publish your study in the Resource section of EMBO Reports and I have therefore changed the article type from "Article" to "Resource".*

OK

*- Please add up to five keywords.*

We have now added five keywords

*- Funding: The information listed in the manuscript does not match with the funding info supplied in our online submission system. Please double-check and correct this information. Please also note that the funding information should be included in the Acknowledgements section.*

We have checked the funding entries and corrected any mistakes both online and acknowledgements section.

*- Appendix: Please add a/the title and page numbers to the table of content.*

We've now added titles of each of the appendix figures to the table of contents, in addition to the page number that was already present.

*- I noticed that you mention a Reagents and Tools table in the Author Checklist but have not supplied such a table. Please either update the information in the Checklist or provide the table in case it is missing.*

We apologize for the missing file, it has now been added to the submission as a reagents and tools table.

*- Data availability section: Please add links that resolve to the datasets on MissIVE. Please remove the reviewer tokens and please remove the sentence "Plasmids are available from the corresponding author upon reasonable request" since this section is only meant to provide information on publicly deposited datasets.*

We removed the sentence as suggested, and have removed the reviewer credentials from the MassIVE dataset. We've made the dataset public, and have updated the data availability section with the corresponding MassIVE ID and link.

*- Please order the manuscript sections following the Discussion as follows:*
*Materials and Methods*
*Data availability*
*Acknowledgements*
*Author contributions*
*Disclosure and competing interests statement*
*References*
*Figure legends*
*Expanded View Figure legends*

We've now reorganized the manuscript along these instructions.

*- During our routine image analysis that we perform on all manuscripts prior to publication, we noted the blots shown have rather high contrast settings (Fig. EV1D, EV1E, EV5D). Please apply as little contrast/brightness modification as possible and please also supply the unmodified source data for these blots upon resubmission.*

We have included the original blots in the added source data files for these three figure panels (Fig. EV1D, EV1E, and EV5D).

*- I attach to this email a related manuscript file with comments by our data editors (plus some minor changes to the abstract). Please address all comments and upload a revised file with tracked changes with your final manuscript submission.*

We addressed all of the comments and modifications in the attached manuscript file and the submitted the manuscript with tracked changes.

*- Finally, EMBO reports papers are accompanied online by A) a short (1-2 sentences) summary of the findings and their significance, B) 2-3 bullet points highlighting key results and C) a synopsis image that is 550x200-600 pixels large (width x height) in .png format. You can either show a model or key data in the synopsis image. Please note that the size is rather small and that text needs to be readable at the final size. Please send us this information along with the revised manuscript.*

We have added a short summary, bullet points and a synopsis image to the submission.

*We look forward to seeing a final version of your manuscript as soon as possible.*

*With kind regards,*

*Martina Rembold, PhD*
*Senior Editor*
*EMBO reports*

************************

Referee #1:

The authors have addressed the reviewers' concerns, in part through addition of some further co-IP validation data in Figure EV2B (although these were transient overexpression experiments), and also inclusion of some new RTK localization IF data added in Figure EV2A. Overall this is improved, although since HEK293 cells are transformed by AdV5 E1A/E1B and of embryonic origin, and a significant number of the RTKs they express may not be physiologically relevant. Also, it seems unlikely that most of these RTKs are functionally activated when HEK293 cells are grown in culture, as suggested, since this would require expression of the cognate ligands for autocrine stimulation. Nonetheless, as a whole this RTK interactome dataset should be useful to the RTK community.

*1. Figure EVF2A: This new experiment needs a more detailed description. It is really hard to tell from these single cell images (how representative are they?), what fraction of the expressed RTK was at the plasma membrane; in some cases, there is what seems to be plasma membrane localization, but in other it appears that most of the RTK protein was intracellular, possibly in vesicular structures, which is likely to allow nonphysiological interactions. What one would really like to see is the comparative localizations of the tagged RTK and the endogenous RTK (for an RTK for which a good anti-RTK IF antibody is available), but this would be beyond the scope of the paper. Theis issue deserves a more extensive discussion than it gets on page 4.*

This experiment was actually already in the initial submission, however, we have now expanded the discussion on page 4 relating to these microscopy experiments, image analysis and data interpretation. We have also included short discussion and possible shortcomings of microscopy analyses.

*2. Figure EVF2B: The legend needs to describe what was done, and in particular which anti-tag antibody was used for IP and which for blotting the dotted samples - this is not even clear in the new section of the methods describing the dot blotting procedure.*

We have now expanded the Extended View Figure 2B legend and the methods section in the article, relating to the Figure EVF2B to better describe the experiments and for example the antibodies used.

*3. Page 5/Figure EV3: There is no description of or citation for UltraID in the text legend or methods (there is some information in the rebuttal, but this needs to be in the paper), which makes it impossible to understand what comparison was carried out for NTRK3 and what it means! This needs to be remedied.*

The reference and citation to "*Zhao, X., Bitsch, S., Kubitz, L., Schmitt, K., Deweid, L., Roehrig, A., Barazzone, E.C., Valerius, O., Kolmar, H., and Béthune, J. (2021). ultraID: a compact and efficient enzyme for proximity-dependent biotinylation in living cells. bioRxiv 10.1101/2021.1106.1116.448656 [PREPRINT]*" was already in the revised version of the manuscript. We apologize that this was missed by the reviewer. We have now expanded the description of the UltraID, cite the preprint now on multiple sections of the manuscript, and figure legend. We believe this criticism has now been remedied.

Markku Varjosalo
University of Helsinki
Institute of Biotechnology
Biocenter 3, P.O.Box 65 (Viikinkaari 1)
Helsinki 00014 UH
Finland

Dear Dr. Varjosalo,

I am very pleased to accept your manuscript for publication in the next available issue of EMBO reports. Thank you for your contribution to our journal.

At the end of this email I include important information about how to proceed. Please ensure that you take the time to read the information and complete and return the necessary forms to allow us to publish your manuscript as quickly as possible.

As part of the EMBO publication's Transparent Editorial Process, EMBO reports publishes online a Review Process File to accompany accepted manuscripts. As you are aware, this File will be published in conjunction with your paper and will include the referee reports, your point-by-point response and all pertinent correspondence relating to the manuscript.

If you do NOT want this File to be published, please inform the editorial office within 2 days, if you have not done so already, otherwise the File will be published by default [contact: emboreports@embo.org]. If you do opt out, the Review Process File link will point to the following statement: "No Review Process File is available with this article, as the authors have chosen not to make the review process public in this case." Please note that the author checklist will still be published even if you opt out of the transparent process.

Thank you again for your contribution to EMBO reports and congratulations on a successful publication. Please consider us again in the future for your most exciting work.

Yours sincerely,

Martina Rembold, PhD
Senior Editor
EMBO reports

***********************************************************************************

THINGS TO DO NOW:

Please note that you will be contacted by Wiley Author Services to complete licensing and payment information. The required 'Page Charges Authorization Form' is available here: https://www.embopress.org/pb-assets/embo-site/er_apc.pdf

You will receive proofs by e-mail approximately 2-3 weeks after all relevant files have been sent to our Production Office; you should return your corrections within 2 days of receiving the proofs.

Please inform us if there is likely to be any difficulty in reaching you at the above address at that time. Failure to meet our deadlines may result in a delay of publication, or publication without your corrections.

All further communications concerning your paper should quote reference number EMBOR-2021-54041V3 and be addressed to emboreports@wiley.com.

Should you be planning a Press Release on your article, please get in contact with emboreports@wiley.com as early as possible, in order to coordinate publication and release dates.

## EMBO Press Author Checklist

| | |
|---|---|
| Corresponding Author Name: Markku Varjosalo | |
| Journal Submitted to: EMBO reports | |
| Manuscript Number: EMBOR-2021-54041V2 | |

**USEFUL LINKS FOR COMPLETING THIS FORM**
The EMBO Journal - Author Guidelines
EMBO Reports - Author Guidelines
Molecular Systems Biology - Author Guidelines
EMBO Molecular Medicine - Author Guidelines

**Reporting Checklist for Life Science Articles (updated January 2022)**
This checklist is adapted from Materials Design Analysis Reporting (MDAR) Checklist for Authors. MDAR establishes a minimum set of requirements in transparent reporting in the life sciences (see Statement of Task: 10.31222/osf.io/9sm4x). Please follow the journal's guidelines in preparing your manuscript.
**Please note that a copy of this checklist will be published alongside your article.**

**Abridged guidelines for figures**
**1. Data**
The data shown in figures should satisfy the following conditions:
- the data were obtained and processed according to the field's best practice and are presented to reflect the results of the experiments in an accurate and unbiased manner.
- ideally, figure panels should include only measurements that are directly comparable to each other and obtained with the same assay.
- plots include clearly labeled error bars for independent experiments and sample sizes. Unless justified, error bars should not be shown for technical replicates.
- if n<5, the individual data points from each experiment should be plotted. Any statistical test employed should be justified.
- Source Data should be included to report the data underlying figures according to the guidelines set out in the authorship guidelines on Data Presentation.

**2. Captions**
Each figure caption should contain the following information, for each panel where they are relevant:
- a specification of the experimental system investigated (eg cell line, species name).
- the assay(s) and method(s) used to carry out the reported observations and measurements.
- an explicit mention of the biological and chemical entity(ies) that are being measured.
- an explicit mention of the biological and chemical entity(ies) that are altered/varied/perturbed in a controlled manner.
- the exact sample size (n) for each experimental group/condition, given as a number, not a range;
- a description of the sample collection allowing the reader to understand whether the samples represent technical or biological replicates (including how many animals, litters, cultures, etc.).
- a statement of how many times the experiment shown was independently replicated in the laboratory.
- definitions of statistical methods and measures:
  - common tests, such as t-test (please specify whether paired vs. unpaired), simple χ2 tests, Wilcoxon and Mann-Whitney tests, can be unambiguously identified by name only, but more complex techniques should be described in the methods section;
  - are tests one-sided or two-sided?
  - are there adjustments for multiple comparisons?
  - exact statistical test results, e.g., P values = x but not P values < x;
  - definition of 'center values' as median or average;
  - definition of error bars as s.d. or s.e.m.

**Please complete ALL of the questions below.**
**Select "Not Applicable" only when the requested information is not relevant for your study.**

**Materials**

| Newly Created Materials | Information included in the manuscript? | In which section is the information available? (Reagents and Tools Table, Materials and Methods, Figures, Data Availability Section) |
|---|---|---|
| New materials and reagents need to be available; do any restrictions apply? | Not Applicable | |

| Antibodies | Information included in the manuscript? | In which section is the information available? (Reagents and Tools Table, Materials and Methods, Figures, Data Availability Section) |
|---|---|---|
| For **antibodies** provide the following information:<br>- Commercial antibodies: RRID (if possible) or supplier name, catalogue number and or/clone number<br>- Non-commercial: RRID or citation | Yes | Reagents and Tools table, Materials and methods |

| DNA and RNA sequences | Information included in the manuscript? | In which section is the information available? (Reagents and Tools Table, Materials and Methods, Figures, Data Availability Section) |
|---|---|---|
| **Short novel DNA or RNA including primers, probes**: provide the sequences. | Not Applicable | |

| Cell materials | Information included in the manuscript? | In which section is the information available? (Reagents and Tools Table, Materials and Methods, Figures, Data Availability Section) |
|---|---|---|
| **Cell lines:** Provide species information, strain. Provide accession number in repository **OR** supplier name, catalog number, clone number, and/**OR** RRID. | Yes | Reagents and tools table, Materials and methods, Results |
| **Primary cultures:** Provide species, strain, sex of origin, genetic modification status. | Not Applicable | |
| Report if the cell lines were recently **authenticated** (e.g., by STR profiling) and tested for mycoplasma contamination. | Yes | Methods section |

| Experimental animals | Information included in the manuscript? | In which section is the information available? (Reagents and Tools Table, Materials and Methods, Figures, Data Availability Section) |
|---|---|---|
| **Laboratory animals or Model organisms:** Provide species, strain, sex, age, genetic modification status. Provide accession number in repository **OR** supplier name, catalog number, clone number, **OR** RRID. | Not Applicable | |
| **Animal observed in or captured from the field:** Provide species, sex, and age where possible. | Not Applicable | |
| Please detail **housing and husbandry conditions**. | Not Applicable | |

| Plants and microbes | Information included in the manuscript? | In which section is the information available? (Reagents and Tools Table, Materials and Methods, Figures, Data Availability Section) |
|---|---|---|
| **Plants:** provide species and strain, ecotype and cultivar where relevant, unique accession number if available, and source (including location for collected wild specimens). | Not Applicable | |
| **Microbes:** provide species and strain, unique accession number if available, and source. | Not Applicable | |

| Human research participants | Information included in the manuscript? | In which section is the information available? (Reagents and Tools Table, Materials and Methods, Figures, Data Availability Section) |
|---|---|---|
| If collected and within the bounds of privacy constraints report on age, sex and gender or ethnicity for all study participants. | Not Applicable | |

| Core facilities | Information included in the manuscript? | In which section is the information available? (Reagents and Tools Table, Materials and Methods, Figures, Data Availability Section) |
|---|---|---|
| If your work benefited from core facilities, was their service mentioned in the acknowledgments section? | Not Applicable | |

**Design**

| Study protocol | Information included in the manuscript? | In which section is the information available? (Reagents and Tools Table, Materials and Methods, Figures, Data Availability Section) |
|---|---|---|
| If study protocol has been **pre-registered, provide DOI in the manuscript**. For clinical trials, provide the trial registration number **OR** cite DOI. | Not Applicable | |
| Report the **clinical trial registration number** (at ClinicalTrials.gov or equivalent), where applicable. | Not Applicable | |

| Laboratory protocol | Information included in the manuscript? | In which section is the information available? (Reagents and Tools Table, Materials and Methods, Figures, Data Availability Section) |
|---|---|---|
| Provide DOI OR other citation details if **external detailed step-by-step protocols** are available. | Yes | Materials and methods |

| Experimental study design and statistics | Information included in the manuscript? | In which section is the information available? (Reagents and Tools Table, Materials and Methods, Figures, Data Availability Section) |
|---|---|---|
| Include a statement about **sample size** estimate even if no statistical methods were used. | Yes | Materials and methods, results, figure legends |
| Were any steps taken to minimize the effects of subjective bias when allocating animals/samples to treatment (e.g. **randomization procedure**)? If yes, have they been described? | Not Applicable | |
| Include a statement about **blinding** even if no blinding was done. | Yes | Methods section |
| Describe **inclusion/exclusion criteria** if samples or animals were excluded from the analysis. Were the criteria pre-established?<br><br>If sample or data points were omitted from analysis, report if this was due to attrition or intentional exclusion and provide justification. | Not Applicable | |
| For every figure, are **statistical tests** justified as appropriate? Do the data meet the assumptions of the tests (e.g., normal distribution)? Describe any methods used to assess it. Is there an estimate of variation within each group of data? Is the variance similar between the groups that are being statistically compared? | Yes | Materials and methods, figure legends |

| Sample definition and in-laboratory replication | Information included in the manuscript? | In which section is the information available? (Reagents and Tools Table, Materials and Methods, Figures, Data Availability Section) |
|---|---|---|
| In the figure legends: state number of times the experiment was **replicated** in laboratory. | Yes | Figure legends |
| In the figure legends: define whether data describe **technical or biological replicates**. | Yes | Figure legends |

## Ethics

| Ethics | Information included in the manuscript? | In which section is the information available? (Reagents and Tools Table, Materials and Methods, Figures, Data Availability Section) |
|---|---|---|
| Studies involving **human participants**: State details of **authority granting ethics approval** (IRB or equivalent committee(s), provide reference number for approval. | Not Applicable | |
| Studies involving **human participants**: Include a statement confirming that **informed consent** was obtained from all subjects and that the experiments conformed to the principles set out in the WMA Declaration of Helsinki and the Department of Health and Human Services Belmont Report. | Not Applicable | |
| Studies involving **human participants:** For publication of **patient photos**, include a statement confirming that consent to publish was obtained. | Not Applicable | |
| Studies involving experimental **animals**: State details of **authority granting ethics approva**l (IRB or equivalent committee(s), provide reference number for approval. Include a statement of compliance with ethical regulations. | Not Applicable | |
| Studies involving **specimen and field samples:** State if relevant **permits** obtained, provide details of authority approving study; if none were required, explain why. | Not Applicable | |

| Dual Use Research of Concern (DURC) | Information included in the manuscript? | In which section is the information available? (Reagents and Tools Table, Materials and Methods, Figures, Data Availability Section) |
|---|---|---|
| Could your study fall under dual use research restrictions? Please check biosecurity documents and list of **select agents and toxins** (CDC): https://www.selectagents.gov/sat/list.htm | Not Applicable | |
| If you used a select agent, is the security level of the lab appropriate and reported in the manuscript? | Not Applicable | |
| If a study is subject to dual use research of concern regulations, is the name of the **authority granting approval and reference number** for the regulatory approval provided in the manuscript? | Not Applicable | |

## Reporting

The MDAR framework recommends adoption of discipline-specific guidelines, established and endorsed through community initiatives. Journals have their own policy about requiring specific guidelines and recommendations to complement MDAR.

| Adherence to community standards | Information included in the manuscript? | In which section is the information available? (Reagents and Tools Table, Materials and Methods, Figures, Data Availability Section) |
|---|---|---|
| State if relevant guidelines or checklists (e.g., **ICMJE, MIBBI, ARRIVE, PRISMA**) have been followed or provided. | Not Applicable | |
| For **tumor marker prognostic studies,** we recommend that you follow the **REMARK** reporting guidelines (see link list at top right). See author guidelines, under 'Reporting Guidelines'. Please confirm you have followed these guidelines. | Not Applicable | |
| For **phase II and III randomized controlled trials**, please refer to the **CONSORT** flow diagram (see link list at top right) and submit the CONSORT checklist (see link list at top right) with your submission. See author guidelines, under 'Reporting Guidelines'. Please confirm you have submitted this list. | Not Applicable | |

## Data Availability

| Data availability | Information included in the manuscript? | In which section is the information available? (Reagents and Tools Table, Materials and Methods, Figures, Data Availability Section) |
|---|---|---|
| Have **primary datasets** been deposited according to the journal's guidelines (see 'Data Deposition' section) and the respective accession numbers provided in the Data Availability Section? | Yes | Data availability section |
| Were **human clinical and genomic datasets** deposited in a public access-controlled repository in accordance to ethical obligations to the patients and to the applicable consent agreement? | Not Applicable | |
| Are **computational models** that are central and integral to a study available without restrictions in a machine-readable form? Were the relevant accession numbers or links provided? | Not Applicable | |
| If publicly available data were reused, provide the respective **data citations in the reference list**. | Yes | Databases -section in the methods, and corresponding references |
